# The early metabolomic response of adipose tissue during acute cold exposure in mice

**DOI:** 10.1038/s41598-017-03108-x

**Published:** 2017-06-14

**Authors:** Xiyuan Lu, Ashley Solmonson, Alessia Lodi, Sara M. Nowinski, Enrique Sentandreu, Christopher L. Riley, Edward M. Mills, Stefano Tiziani

**Affiliations:** 1 Department of Nutritional Sciences & Dell Pediatric Research Institute, The University of Texas at Austin, 1400 Barbara Jordan Blvd., Austin, TX 78723 USA; 2 Division of Pharmacology and Toxicology, College of Pharmacy, The University of Texas, Austin, Texas 78712 USA; 3 Institute for Cellular and Molecular Biology, The University of Texas at Austin, Austin, Texas 78712 USA

## Abstract

To maintain core body temperature in cold conditions, mammals activate a complex multi-organ metabolic response for heat production. White adipose tissue (WAT) primarily functions as an energy reservoir, while brown adipose tissue (BAT) is activated during cold exposure to generate heat from nutrients. Both BAT and WAT undergo specific metabolic changes during acute cold exposure. Here, we use an untargeted metabolomics approach to characterize the initial metabolic response to cold exposure in multiple adipose tissue depots in mice. Results demonstrate dramatically distinct metabolic responses during cold exposure in BAT and WAT. Amino acids, nucleotide pathways, and metabolites involved in redox regulation were greatly affected 4 hours post-exposure in BAT, while no polar metabolites were observed to significantly change in WAT depots up to 6 hours post exposure. Lipid metabolism was activated early (2 hours) in both BAT and the subcutaneous WAT depots, with the most striking change being observed in the modulation of diglyceride and monoglyceride levels in BAT. Overall, these data provide a timeline of global thermogenic metabolism in adipose depots during acute cold exposure. We have highlighted differences in visceral and subcutaneous WAT thermogenic metabolism and demonstrate the distinct metabolism of BAT during cold exposure.

## Introduction

Regulation of body temperature is an essential element of mammalian life which requires activated metabolism in brown and white adipose tissues. The increased number of mitochondria and the expression of uncoupling protein 1 (UCP1) in brown adipose tissue (BAT) support its specialized thermogenic function and distinct metabolic activity^1, 2^. Upon activation by lipid binding, BAT UCP1 promotes uncoupled respiration by dissipating the mitochondrial proton gradient to generate heat^3^. The recent discovery of a population of white adipocytes (beige adipocytes^4, 5^) with the capacity to induce UCP1 and uncoupled respiration has accelerated mechanistic studies to understand the gene regulatory networks that promote thermogenic metabolism^6^. The increased energy expenditure induced by UCP1 is sufficient to combat obesity^7^, encouraging deeper examination of the metabolic events that occur concomitantly with thermogenesis. Beige adipocytes are more abundant in subcutaneous fat compared with visceral fat after cold exposure^8^, and comprehensive studies have demonstrated distinct transcriptional responses in brown, subcutaneous (SWAT) and visceral white (VWAT) adipose tissues during activated thermogenesis^9^. Recent reports have suggested that mitochondrial reactive oxygen species (ROS) play a critical role in regulating thermogenesis and UCP1 activity^10^. Cold exposure is associated with increased mitochondrial superoxide and oxidation of lipids and proteins in BAT^10^. Furthermore, acute cold exposure (3 hours at 4 °C) leads to increased oxidation and depletion of reduced glutathione (GSH) in BAT accompanied by increased protein thiol oxidation, which has been proposed as a vital signaling mechanism required for UCP1-induced thermogenic metabolism^10^. Mice exposed to 4 °C for 20 hours show a similar depletion in GSH level in WAT with no difference observed in BAT at that time point^11^. Additionally, systemic depletion of mitochondrial ROS prior to cold exposure is sufficient to induce hypothermia after one hour cold exposure^10^. Collectively, this evidence suggests there are metabolic events that occur within the initial hours of cold exposure, which are critical for thermogenesis and include mitochondrial ROS signaling, glutathione oxidation and a depletion of GSH occurring temporally in various tissues during cold exposure.

Here, we characterize the metabolome^12–14^, a global set of small molecules, within adipose tissue depots at thermoneutrality (30 °C) and during a time course (2–6 hours) of acute cold exposure by combining high-resolution magnetic resonance spectroscopy (MRS) and ultra-high pressure liquid chromatography-mass spectrometry (UHPLC-MS) analytical platforms^13, 15–17^. Our analysis of adipose tissue metabolism supports the findings that BAT thermogenesis is associated with glutathione depletion after acute cold exposure. Interestingly, the cold-induced, global metabolic response in WAT, from both subcutaneous and visceral origins, occurred later and/or slower than in BAT: no significant changes were observed in polar metabolite levels in SWAT and VWAT at any time point during the 6 hours of cold exposure. Lipid metabolites in SWAT had a transitory response to cold exposure, with significant modulation of several lipid metabolites after 2–4 hours of cold exposure, followed by a return to thermoneutral levels at 6 hours. The study design and technologies applied have generated a data set of acute cold-induced metabolism in multiple tissues simultaneously that may guide further investigations of thermogenic regulation and support the development of anti-obesity therapeutics that target thermogenic mechanisms^4, 5, 18^.

## Results

### Multivariate statistical analysis reveals metabolic effects on adipose tissues during early acute cold-exposure

To determine the variation in metabolic profiles of different adipose tissues and investigate the metabolic modulations induced in each of these tissues by acute cold exposure, population-based studies were performed on four groups of 6 mice (male to female ratio = 3:3) each exposed to 4 °C, for 0 (thermoneutral control), 2, 4 or 6 hours. SWAT (50.9 ± 15.4 mg), VWAT (56.0 ± 15.6 mg) and BAT (37.1 ± 8.5 mg) were collected, immediately frozen in liquid nitrogen, and maintained at −80 °C until sample preparation and metabolomics data acquisition.

Comprehensive metabolic profiles were acquired from tissue extracts. The aqueous fraction, obtained from a dual phase extraction, was analyzed using a combination of MRS and UHPLC-MS, while the apolar fraction was analyzed using UHPLC-MS. A total of 3676 and 1977 features were detected for the polar and lipid fractions, respectively. In order to visualize and help to identify the most prominent metabolic differences between the adipose tissues, principal component analysis (PCA) was performed on all the detected features.

**Figure 1:**
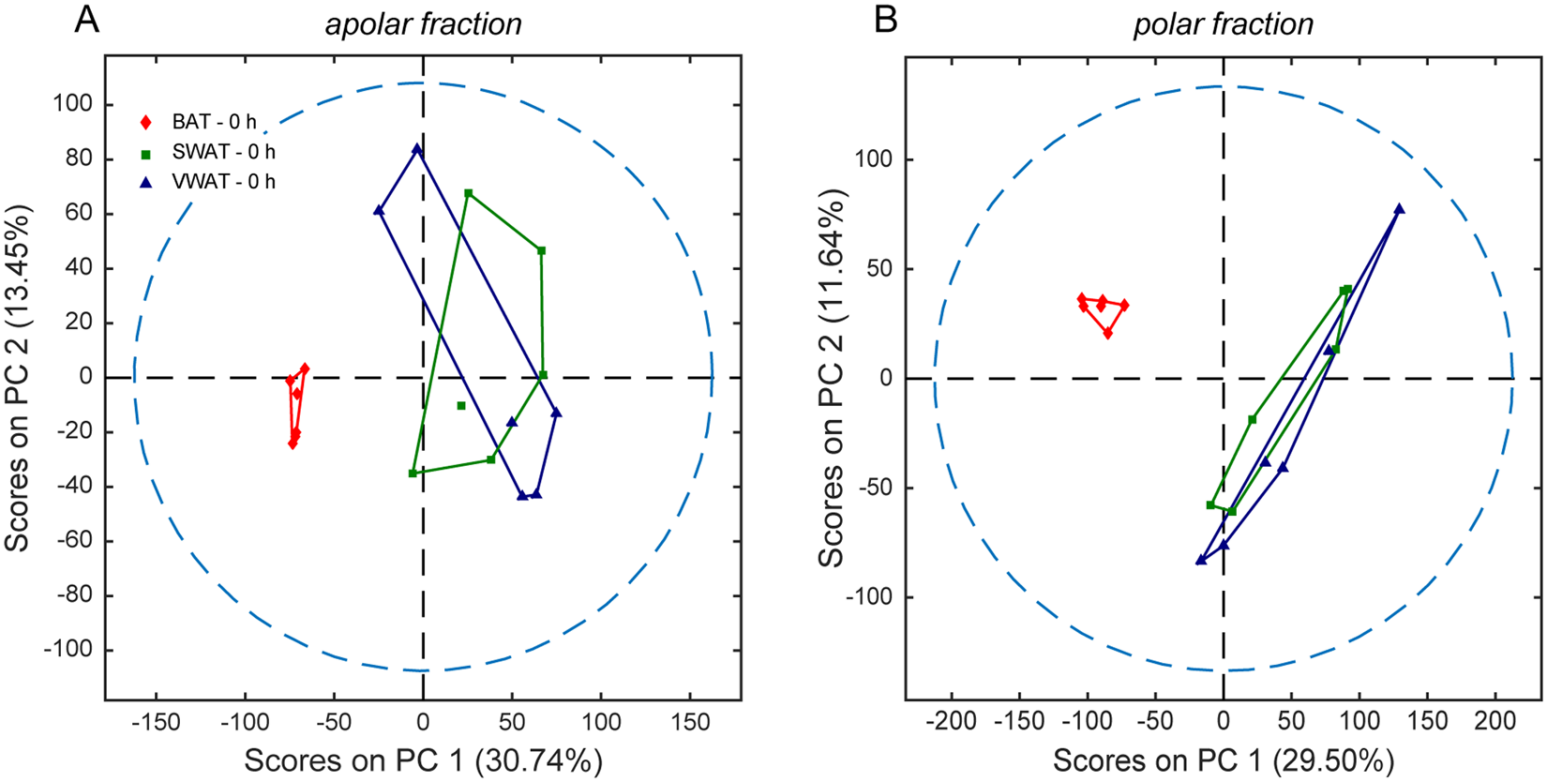
Metabolic differences in adipose tissues from different depots at thermoneutrality. An untargeted multilevel PCA was performed on combined HPLC-MS and MRS data acquired on BAT, SWAT and VWAT sample extracts at thermoneutrality. Score plots obtained from PCA performed on apolar (**A**) and polar (**B**) spectra of BAT, SWAT, and VWAT samples (6 replicates per tissue type) are shown.

**Figure 2:**
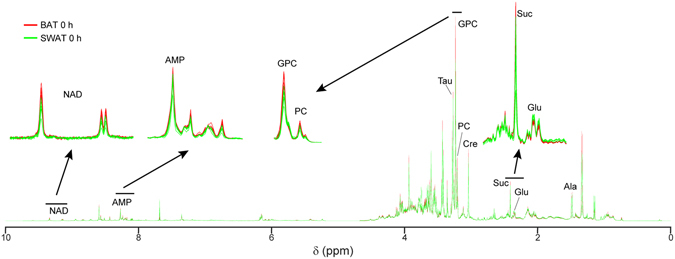
MRS metabolic profiles of BAT and SWAT samples at thermoneutrality. The MRS spectra acquired on BAT (red lines) and SWAT (green lines) samples (6 replicates each) are shown. Resonances of selected biomarkers are magnified above the full spectrum. NAD: nicotinamide adenine dinucleotide; AMP: adenosine monophosphate; TAU: taurine; GPC: sn-glycero-3-phosphocholine; PC: phosphocholine; Cre: creatine; Suc: succinate; Glu: glutamate; Ala: alanine.

Metabolic differences between adipose tissues from different depots (BAT, SWAT and VWAT) were evaluated at thermoneutrality (Fig. 1) and following cold exposure (Supplementary Fig. 1) using PCA. Additionally, the consequences of cold exposure on the metabolic profile of each adipose tissue were investigated using PCA score plots of the apolar and polar metabolic profiles from each tissue at thermoneutrality and following cold exposure. Figure 1 displays separation of BAT and WAT along the first principal component (with 30.74% and 29.50% of variances for the lipid and polar fractions, respectively) at thermoneutrality in both the apolar and polar fractions, indicating relevant differences in their basal metabolic activities. As shown in Fig. 2, several polar metabolites detected by MRS dictate the differences between brown and subcutaneous white adipocytes. Virtually no differences were observed at thermoneutrality between the metabolic profiles of SWAT and VWAT (Fig. 1). However, cold exposure induced distinguishable changes in lipid metabolism in these two depots (Supplementary Fig. 1A,C and E), consistent with previous reports^9, 19^.

**Figure 3:**
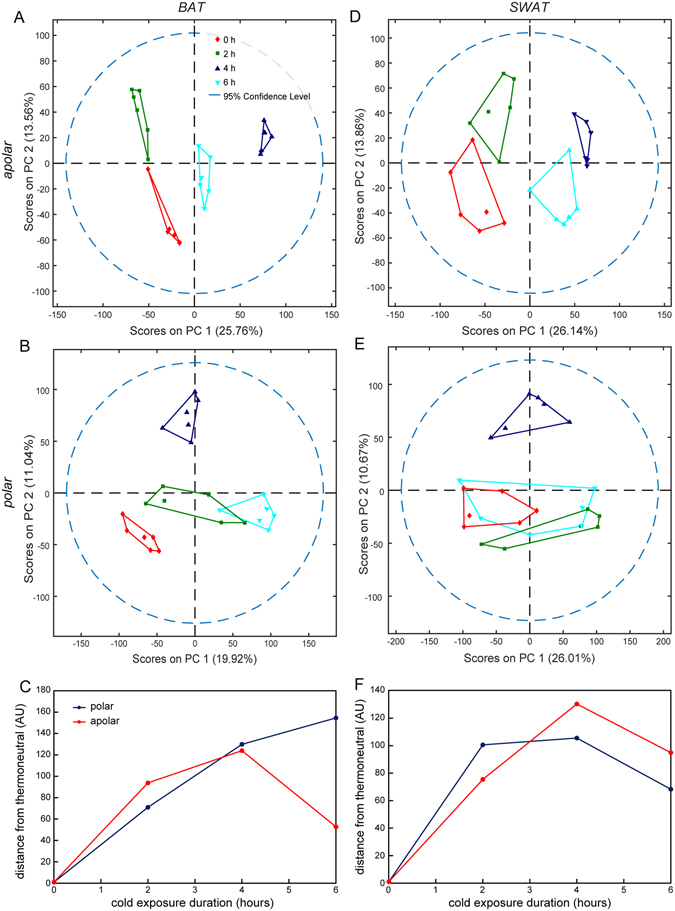
Metabolic changes in adipose tissues from different depots during cold exposure. An untargeted multilevel PCA was performed on combined HPLC-MS and MRS data acquired on BAT and SWAT sample extracts collected at thermoneutrality, and after 2, 4 and 6 hours of cold exposure. Score plots obtained from PCA performed on apolar and polar spectra of BAT (**A**,**B**) and SWAT (**D**,**E**) (6 replicates per tissue type) are shown. The average distance of each cold-exposure group from thermoneutrality was calculated for both polar and apolar sample extracts and for both BAT (**C**) and SWAT (**F**) depots.

Metabolic profiles induced by cold exposure specific to each adipose tissue (Fig. 3 and Supplementary Fig. 2) were also evaluated using PCA. Score plots for BAT identified distinct lipid metabolism at each time point during cold exposure (Fig. 3A); whereas, the polar fraction showed partially overlapping profiles in BAT at 2 and 6 hours post-exposure and divergent profiles at thermoneutrality and 4 hours (Fig. 3B). The distance between the BAT thermoneutral group profile and each of the cold-exposure group profiles was quantified in the PCA score plots of both the apolar and polar fractions (Fig. 3C **)**. Notably, the distance from thermoneutrality of the polar metabolic profiles increased in a near linear pattern, whereas the lipid profiles increased to a maximum distance at 4 hours of cold exposure, followed, at 6 hours post-exposure, by a return towards a profile more similar to thermoneutrality. A similar analysis performed on SWAT profiles indicated that lipid metabolism was more markedly influenced by cold exposure than polar metabolites (Fig. 3D,E). Interestingly, as observed for BAT, the 4 h time point induced the most extensive changes in SWAT (in this case for both the polar and apolar metabolites; Fig. 3F), while the 2 h and 6 h cold-exposure profiles group closer to thermoneutrality. Score plots of VWAT showed very similar lipid profiles at thermoneutrality and for short (2 h) cold exposure, with distinct profiles observed after longer cold exposures (4 h and 6 h). The polar fraction of VWAT showed only minor changes occurring throughout the course of cold exposure (Supplementary Fig. 2).

#### Amino acids, purine, pyrimidine and metabolites involved in redox regulation are significantly modulated by cold exposure in BAT but not in WAT depots

**Figure 4:**
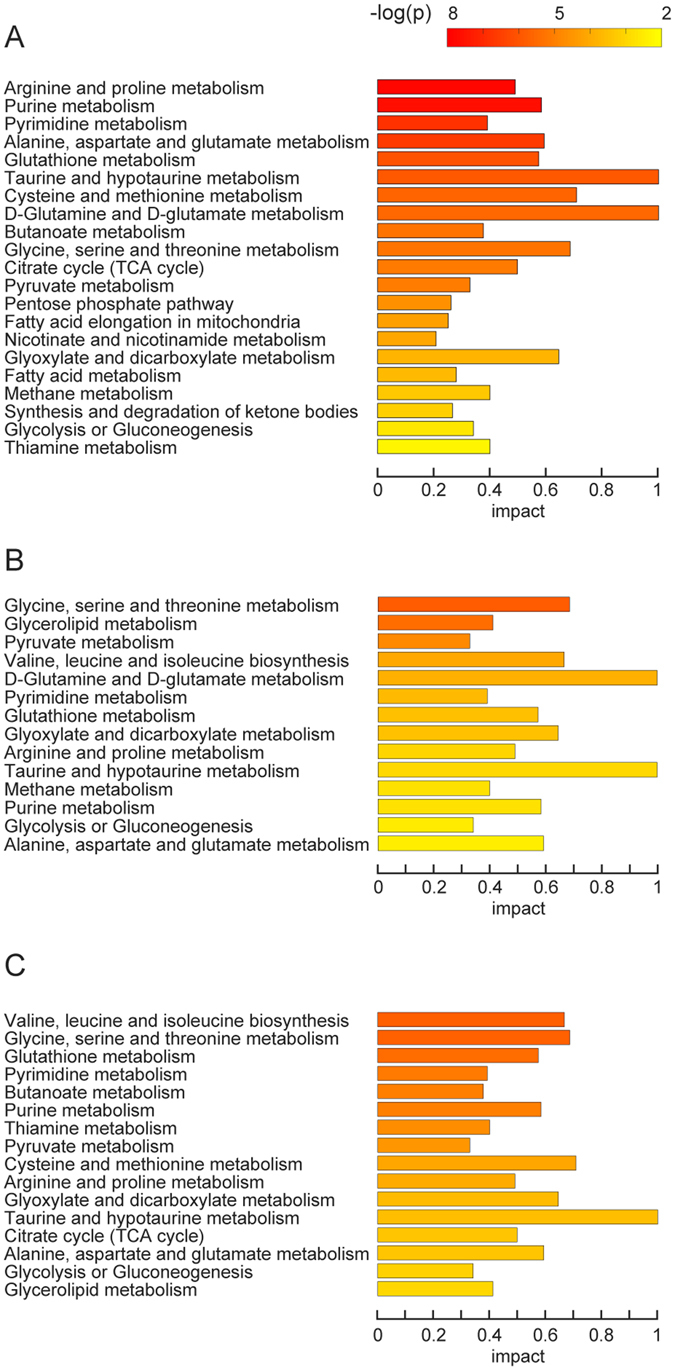
Metabolic pathways affected by cold exposure in adipose tissues from different depots. Combined pathway enrichment and pathway topology analyses were performed based on the changing polar metabolic profiles in BAT (**A**), SWAT (**B**) and VWAT (**C**) during cold exposure. Bar colors reflect the significance (p value) for the specific pathway. The length of each bar indicates the impact value of each metabolic pathway. Pathways with p < 0.01, FDR < 0.01, impact > 0.2 are shown.

To determine more specifically which metabolic alterations are associated with initial cold exposure, a total of 175 polar compounds (Supplementary Table 1) and 504 apolar metabolites (Supplementary Table 2) were identified. Metabolic pathway analysis by global test (performed on the polar metabolites from each tissue) revealed that cold exposure induced the highest impact on metabolic pathways in BAT (Fig. 4 includes pathways with p < 0.01, FDR < 0.01, impact > 0.2), while metabolic pathways were affected to a lesser extent in SWAT and VWAT. Among the pathways most affected by cold-exposure in BAT were amino acids, purine and pyrimidine metabolism, as well as pathways involved in redox regulation (Fig. 4A).

**Figure 5:**
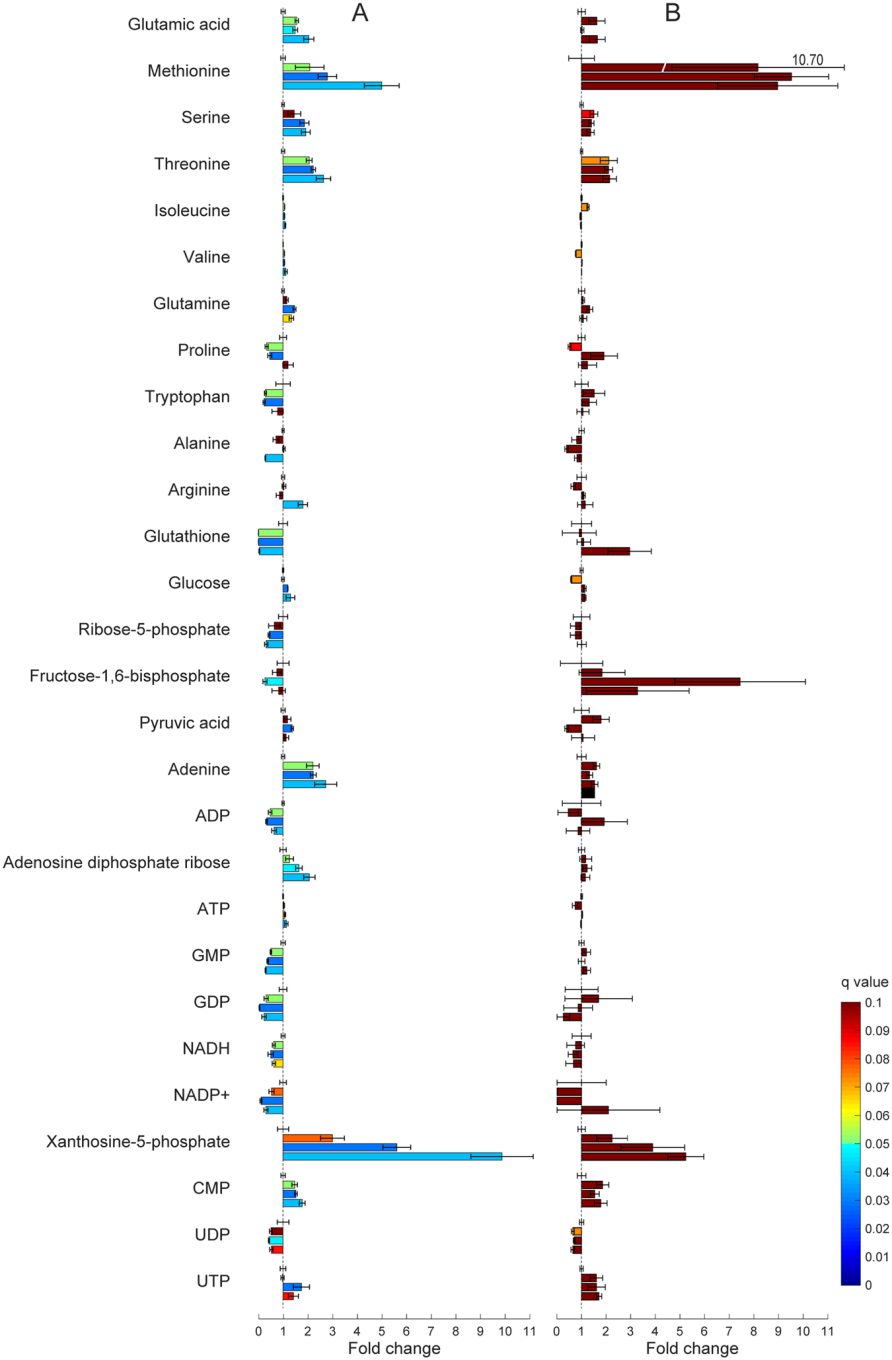
Metabolic changes in BAT and SWAT samples during cold exposure. Fold changes (compared to thermoneutrality; mean ± standard error, n = 6 mice) in selected metabolite levels in BAT (**A**) and SWAT (**B**) samples collected at 0, 2, 4, and 6 hours. For each metabolite 4 bars are shown: the top bar represents thermoneutrality (always shown at 1 ± standard error), and the others (top to bottom) indicate 2, 4 and 6 hours cold-exposure fold change vs thermoneutrality. Statistical significance was calculated by non-parametric Wilcoxon signed-rank tests with FDR. Bars are colored according to q-value.

Following up on the results from the metabolic pathway analysis, we performed further analysis of the specific polar metabolite levels in BAT and WAT depots. These data indicated that several metabolites were significantly modulated (FDR q < 0.05) in BAT following 4–6 h cold-exposure, with no metabolites reaching significance at 2 hours in BAT or after any duration of cold exposure in WAT depots (Fig. 5, Supplementary Fig. 3 and Supplementary Table 1). The levels of several amino acids increased significantly after 4–6 hours of cold exposure (glutamic acid, methionine, serine, threonine, isoleucine and valine). Glutamine accumulated while proline and tryptophan levels decreased transiently at 4 hours. Alanine and arginine levels were significantly modulated only after 6 hours of cold exposure. Also the levels of glutathione dropped significantly (to almost undetectable levels) after 4–6 hours of cold-exposure in BAT (and was markedly decreased with q = 0.052 after only 2 hours). Other modulations were observed in glycolysis and pentose phosphate pathway metabolites, with the progressive accumulation of glucose and drop of ribose-5-phosphate after 4 and 6 hours as well as the transitory pyruvate increase and fructose bisphosphate decrease at 4 hours of cold exposure in BAT. Interestingly, significant modulations in pyrimidine and, even more so, purine levels in BAT were also observed after 4 hours of cold exposure.

#### Di- and monoglyceride levels are almost univocally modulated by cold exposure in BAT and, moderately, in SWAT

**Figure 6:**
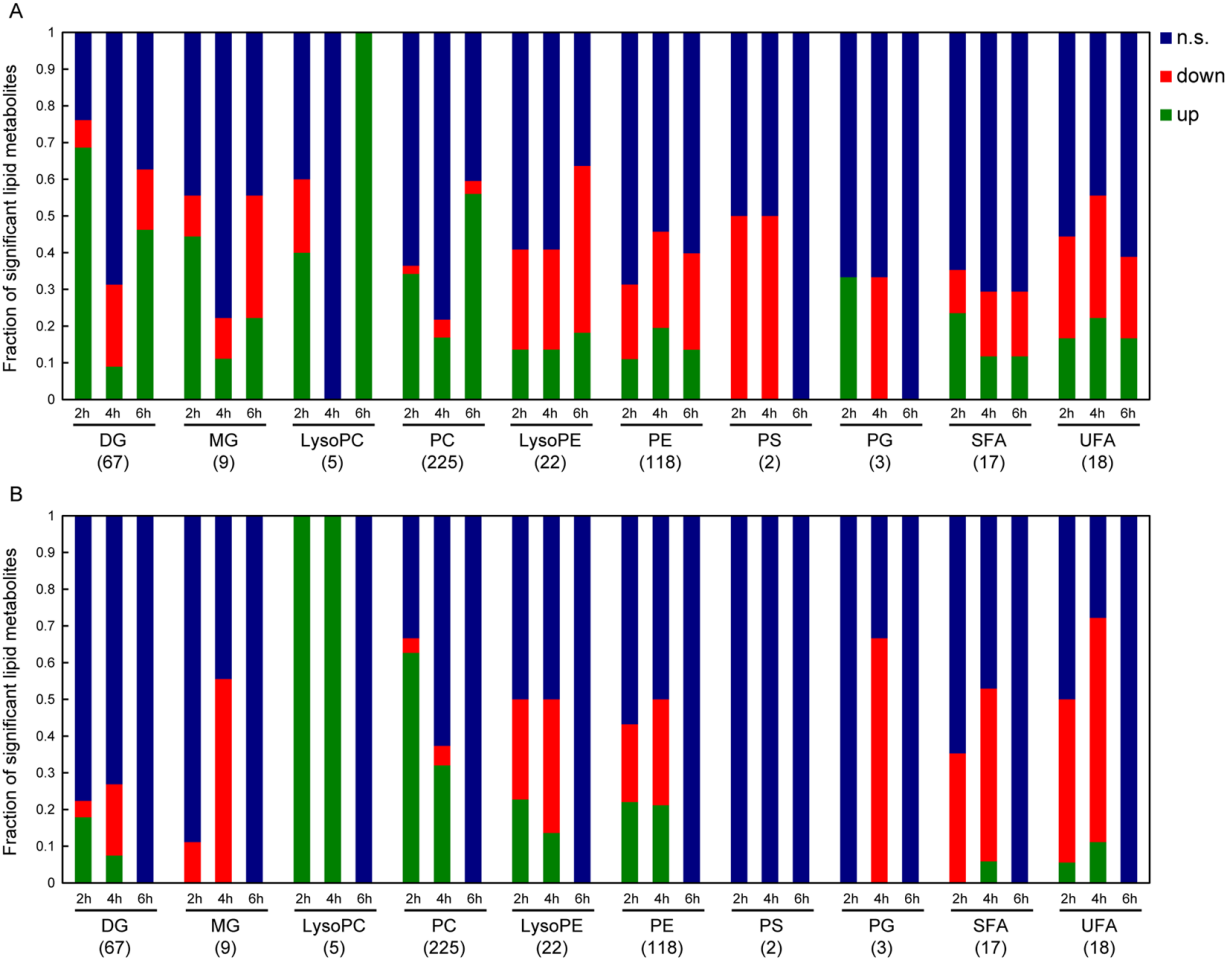
Number of lipid metabolites significantly affected by cold exposure in BAT and SWAT. Fractions of the total number of identified lipids (in parenthesis under the name of the lipid class) in each lipid class that are significantly increased (green), significantly decreased (red) and unchanged (blue; n.s.: non-significant) at 2, 4 and 6 hours cold exposure in BAT (**A**) and SWAT (**B**). DG: diglycerides; MG: monoglycerides; LysoPC: lysophosphatidylcholines; PC: phosphatidylcholines; LysoPE: lysophosphatidylethanolamines; PE: phosphoethanolamines; PS: phosphatidylserines; PG: prostaglandins; SFA: saturated fatty acids; UFA: unsaturated fatty acids.

An in depth analysis of the apolar metabolites revealed that, in line with the results of the polar fraction, metabolic changes induced by cold exposure occur to a much greater extent in BAT than in WAT depots (Supplementary Table 2). However, a larger fraction of the apolar metabolites was significantly modulated in SWAT at 2 (251 out of the total 504 identified lipids) and 4 (214 out of 504) hours, while none of the SWAT lipid metabolites changed significantly at 6 hours. Concurrently, in BAT, the largest number of significant changes were observed after 6 hours (269 out of 504). Nonetheless, several (209 out of 504) significant changes were observed as early as 2 h post exposure in BAT, and at 4 hours (214 out of 504). No significant changes were detected in VWAT (at any time point). The fraction of the total number of lipids identified in each lipid class are reported in Fig. 6 to depict the trend of the major lipid classes affected at 2, 4 and 6 hours cold exposure in BAT and SWAT. The majority of the detected diglycerides (DG; 51 out of 67 detected DGs) were significantly modulated (FDR q < 0.05) in BAT after a short 2 h cold exposure (46 DGs accumulated and 5 dropped significantly). Notably, for a majority of the accumulated DGs (at 2 h), the levels dropped after 4 h of cold exposure (mostly to levels comparable to the initial ones) and then increased again to significantly higher levels than before exposure after 6 hours of cold exposure (22 out of 34 DGs that changed significantly compared to thermoneutral at both 2 and 6 hours post exposure). Similarly, of the 5 DGs that decreased significantly after 2 h, 3 also decreased significantly at 6 hours and all of them appeared to bounce back at 4 h and then drop again at 6 h. Comparable trends were observed for the few MGs that were detected and changed significantly during cold exposure. No significant changes in DG were detected in VWAT, while 15 DGs changed significantly after 2 h (mostly DG accumulation, 12 out of 15) of cold exposure in SWAT. Only 5 of these were also significantly modulated at 4 h and mostly returned to levels closer to thermoneutrality.

## Discussion

In this study, the metabolic profile of BAT, and WAT of subcutaneous and visceral origin were analyzed at thermoneutrality and during acute cold exposure (at 2, 4 and 6 hours). BAT profiles of both the polar and apolar intracellular metabolite fractions differed dramatically from WAT depots.

Acute cold exposure induced dramatic metabolic changes in BAT with many significant changes occurring in both lipid and polar metabolites. The PCA performed on the lipid fraction indicated that distance from thermoneutrality occurred at an almost linear rate up to 4 h after which the profile shifted to be more similar to the thermoneutral profile between 4–6 h. Notably, significant changes in the lipid fraction were observed after only 2 hours post exposure in both BAT and SWAT (Supplementary Table 2). The most striking changes in lipid metabolites observed in BAT related to hydrolysis of triglycerides (TGs) into DGs, MGs and fatty acids. In fact, the large majority of the identified DGs and some of the MGs increased significantly during the very early phase of cold exposure followed by a transient normalization towards pre-exposure levels at 4 hours and another increase at 6 hours post exposure. In line with previous reports, these observations also provide a timeline for the BAT activity on TGs outlining the progress of lipolysis within the first few hours following cold exposure. This is in accordance with measured decreases in serum TGs in mice after 4 hour cold exposure, which is dependent upon local lipoprotein lipase (LPL) activity in BAT as previously reported^16^. Consistent with these reports, mice lacking the major fatty acid transporter CD36 displayed a marked decrease in BAT TG content and triglyceride-rich lipoprotein clearance by BAT after 2 hours cold exposure^20^. Collectively, these observations support the notion that proper substrate switching, from oxidation of intracellular fats and proteins to oxidation of WAT derived-TG, is a requirement for cold-induced thermogenesis and likely occurs within 2 hours after initiation of acute cold exposure.

The modulation of polar metabolites in BAT maximally occurred at 4 hours of cold exposure, after the significant lipid changes at 2 hours. Additionally, the PCA score plots suggest that the polar profiles in BAT are more similar to thermoneutral metabolism after 6 hours of cold exposure, suggesting a metabolic cycle may occur in BAT during cold exposure. Interestingly, cold exposure did not induce any significant changes to polar metabolite levels in the WAT depots, but conclusions on this point may be limited by the large variability between the WAT depots samples at thermoneutrality (as indicated by the PCA in Fig 1B).

Mild but significant alterations of glycolysis and pentose phosphate pathway metabolites were observed after 4 hours of cold exposure in BAT indicating a possible transition in the utilization of these pathways. Previous reports have detailed differences in the degree of activation and timeline of activated glycolysis and this is believed to be a consequence of housing conditions prior to cold exposure (22–25 °C versus 28–30 °C). Significant increases in serine observed in this data set may be interesting with regard to cold-induced upregulation of glycolysis^21^. An increase in serine levels has been observed in other studies of cold exposure for 12 hours, which suggests that increased serine levels could serve as an additional marker of cold mediated activation of BAT^22^. The serine biosynthetic pathway derives from, and has been demonstrated to influence glycolysis^23^. Additionally, serine is an important intermediate in one-carbon and glutathione metabolism and is related to cell signaling through mTORC1 activity^24, 25^. Specifically, serine can undergo cleavage to glycine and contributes one carbon units which can support NADPH production and cell proliferation^24^. Within glycolysis, serine has been shown to allosterically activate pyruvate kinase isoform M2 (PKM2) where alterations in serine levels can influence production of pyruvate, through a rapid and reversible mechanism^23^. This could represent a metabolic regulatory point for glycolysis in BAT during cold exposure. Pyruvate kinase is upregulated in BAT during cold exposure, and both PKM1 and PKM2 isoforms are expressed in BAT^26–28^. Our data indicate pyruvate levels increase transitorily during cold exposure in BAT at 4 hours. This result may suggest that regulation of pyruvate kinase and pyruvate levels during cold exposure can modulate glycolysis and pentose phosphate pathway, as occurs in cancer metabolism.

The uptake and cellular consumption of glutamine and its conversion to glutamic acid or alpha-ketoglutarate serves many different metabolic purposes, including increasing the capacity for TCA cycle flux to fatty acid oxidation through generation of C4 metabolites such as malic, oxaloacetic and aspartic acid. Glutamic and aspartic acid are also important for providing substrates for transamination reactions that are necessary for serine and amino acid metabolism. Moreover, glutamine is a nitrogen donor necessary for purine biosynthesis, whose levels were significantly decreased (other than ATP which increased slightly and only at the 6-h time point) at 4–6 hours of cold exposure in BAT; the decreased purine levels may serve to stimulate thermogenesis by decreasing the ability of purine metabolites to inhibit uncoupling protein-1 (UCP1)^29^.

Recent reports suggest that reactive oxygen species are a critical signaling component for acute thermogenesis. Depletion of mitochondrial ROS results in cold-intolerance^10^ and the dramatic depletion of glutathione observed in BAT may act as a vital stimulant of thermogenic programs in adipose tissue^11^. Oxidized glutathione levels did not change during the course of cold exposure supporting evidence that depletion of glutathione is a critical event in regulation of thermogenesis. These data are in congruence with previous report^30^ of glutathione depletion; in addition, the accumulation of glutamic acid, methionine and serine supports the lack of replenishment of glutathione from amino acids and glycolytic intermediates during cold exposure.

Collectively, our data provide a comprehensive timeline of global thermogenic metabolism in adipose depots during acute cold exposure. We have highlighted differences in the VWAT and SWAT thermogenic metabolism and demonstrate the distinct metabolism of BAT during cold exposure.

## Material and Methods

### Study design

C57BL/6 mice (12 week old) purchased from the Jackson Laboratory were acclimated for one week under standard 12/12 hour light dark cycle with ad libitum access to Prolab RMH 2000 5P06 diet. Mice were housed three per cage, and randomly assigned to sex matched groups (N = 6) for 0, 2, 4, or 6 hour cold exposure (4 °C). Experimental design included consideration for circadian rhythm such that all tissues were collected between 14:00–16:00. Prior to cold exposure, cages were placed into thermoneutrality upon timed intervals to ensure that each cage was exposed to 24 hours thermoneutrality (30 °C) prior to cold exposure for the specified duration. Control mice (0 hour or thermoneutral group) were placed at thermoneutrality for 24 hours prior to tissue collection. Upon completion of exposure time, mice were sacrificed using CO_2_ overexposure and adipose tissues were collected from inguinal fat pad for subcutaneous white adipose tissue (SWAT), interscapular brown adipose tissue (BAT), and from gonadal fat pad for visceral white adipose tissue (VWAT) according to Chau *et al*.^31^ and Casteilla *et al*.^32^. Tissues were collected and quickly frozen in liquid nitrogen and stored at −80 °C until further metabolic analysis.

### Solvents and reagents

All chemicals were HPLC grade and all the aqueous solutions were prepared using ultrapure water (Milli-Q system, Millipore, Billerica, MA). Deuterium oxide (D_2_O, 99.8%) for MRS data acquisition was purchased from Acros Organics (Fair Lawn, NJ). Formic acid (FA), acetonitrile (ACN), isopropanol (ISP), ammonium formate (AF, 99.9% purity), methanol (99.9% purity), chloroform (99.8%), and ammonium acetate (AA, 99% purity) were bought from Thermo Fisher Scientific (Pittsburgh, PA, USA). Compounds, including sodium azide (NaN_3_, 99%), sodium dodecyl sulfate, caffeine, caffeine fragment, monosodium phosphate (>99%), and disodium phosphate (>98%), were from Thermo Fisher Scientific (Pittsburgh, PA, USA). Deuterated 3-(trimethylsilyl)-2,2,3,3-tetradeuteropropionic acid (TSP) was from Cambridge Isotope Laboratories (Tewksbury, MA). Mass spectrometry calibration mixtures (Pierce LTQ Velos ESI Positive and Negative Ion calibration solutions) were also purchased from Thermo Fisher Scientific. Compounds for customized calibration, including pyruvic acid, sodium taurocholate, n-butylamine, and Met-Arg-Phe-Ala pentapeptide (MRFA) were purchased from Sigma-Aldrich (St. Louis, MO, USA). The following apolar ISs for mass spectrometry, 1,2-dipalmitoyl-d62-sn-glycero-3-phosphoethanolamine (16:0 PE-d62), 1,2-diphytanoyl-sn-glycero-3-phospho-L-serine (4ME 16:0 PS), 1,2-diphytanoyl-sn-glycero-3-phosphoethanolamine (4ME 16:0 PE), 1-myristoyl-2-hydroxy-sn-glycero-3-phosphocholine (Lyso PC), 1,2-dimyristoyl-d54-sn-glycero-2-[phospho-L-serine] sodium salt (14:0 PS-d54), 1,2-dilauroyl-sn-glycero-2-phosphocholine (12:0 PC), 1,2-dipalmitoyl-d62-sn-glycero-3-phosphocholine-1,1,2,2-d4-N,N,N-trimethyl-d9 (16:0 PC-d75), 1,2-dioleoyl-sn-glycero-3-phosphoinositol ammonium salt (18:1 PI), 1,2-dilauroyl-sn-glycerol (12:0 DG), and Ceramide/Sphingoid Internal Standard Mixture I were acquired from Avanti Polar Lipids Inc. (Alabaster, Alabama). In addition, heptadecanoic-17,17,17-d3 acid was purchased from CDN Isotopes (Pointe-Claire, Quebec, Canada). Water soluble ISs, including cysteine (3,3-D2, 98%), fumaric acid (2,3-D2, 98%), DL-glutamic acid (2,4,4-D3, 98%), tryptophan-d5 indole, d4 cystine, and succinic acid (D4, 98%) were bought from Cambridge Isotope Laboratories (Tewksbury, MA, USA).

### Adipocytes sample preparation

Tissues were homogenized in a Precellys®24 cooled tissue homogenizer equipped with Cryolys® system (Bertin Corp, Rockville, MD, USA) by adding 1 ml cold methanol/H_2_O (50:50) and operating at 6500 rpm × 2 cycles × 20 seconds for four times. Homogenates were transferred into glass vials and washed twice with 500 µl of methanol/H_2_O (50:50). Then, 2 ml of chloroform were added to each vial followed by the addition of 100 µl of the IS solutions (50 µl each of 100 µM and 25 µM of polar and non-polar IS mixtures, respectively) and vortexed thoroughly. Both polar and apolar phases were collected, aliquoted for MRS and MS analyses, dried using a CentriVap refrigerated vacuum concentrator (Labconco, Kansas City, MO, USA) and then immediately stored at −80 °C before the metabolomics analysis.

### MRS-based metabolomics analysis

Polar samples for MRS analysis were resuspended in 45 µl of phosphate buffer (0.1 M phosphate, 90% D_2_O, 1 mM TSP, 0.05% NaN_3_), vortexed, centrifuged at 4 °C for 10 min and finally 35 µl of the supernatants were transferred into 1.7 mm MRS tubes. One dimensional (1D) ^1^H MRS spectra were acquired on a Bruker Avance III 500 MHz with 1.7 mm TCI MicroCryoProbe system (Bruker BioSpin Corp., Billerica, MA) equipped with autosampler at 300 K. 1D ^1^H-MRS spectra were acquired suppressing water resonance by excitation sculpting pulse sequence^33^ and using at least 128 scans and 8 dummy scans, 32768 data points, and a spectral width of 8012.820 Hz. Raw data were processed using MRSLab and MetaboLab^34, 35^ in the MATLAB programming environment (MathWorks Inc., Natick, MA). Post processing of MRS spectra included scaling according to the probabilistic quotient normalization (PQN)^36^, segment alignment^37^, and exclusion of selected signals arising from extraction-solvents and TMSP, and application of a generalized log transformation^38^. Metabolite assignment and quantification were performed using Chenomx 8 MRS Suite (Chenomx Inc., Edmonton, Alberta, Canada), the Birmingham Metabolite Library^39^, and the Human Metabolome Database^40^.

### MS-based metabolomics and lipidomics analyses

Both polar and lipid analyses were performed as previously described^41–43^ on a Q Exactive Hybrid Quadrupole-Orbitrap Mass Spectrometer equipped with an Accela 1250 pump, a vacuum degasser, an open autosampler, and a temperature controller (Thermo Scientific, Waltham, MA).

The polar samples were reconstituted in 1 ml of ultrapure water, vortexed, centrifuged at 4 °C for 10 min and the supernatant was used for MS detection. Separation of polar metabolites was achieved through a 100 mm × 2.1 mm i.d., 5 µm particle size, Millipore ZIC p-HILIC column (Millipore Co., Billerica, MA, USA). Separation conditions were: solvent A, water/FA (99.9:0.1) containing 10 mM AF; solvent B, ACN/FA (99.9:0.1; separation gradient, initially 96% B, linear 96–20% B in 15 min, purging with 1% B for 5 min and column equilibration with 4% B for 10 min; flow rate, 0.3 mL/min; injection volume, 5 µl and 3 µl for scanned m/z ranges of 50–750 and 750–2000, respectively; autosampler temperature, 6 °C; column temperature, 22 °C. Mass spectrometry analysis of polar compounds was performed with the electrospray (ESI) source simultaneously operating in both negative and positive full-MS modes under the following conditions: spray voltage, 4.0 kV; capillary temperature, 300 °C; sheath gas, 50 (arbitrary units); auxiliary gas, 10 (arbitrary units); microscans, 1; AGC target, 1e^6^; maximum injection time, 100 ms; mass resolution, 70,000. To maximize accuracy of the MS analysis of polar metabolites, samples were analyzed twice according to two different *m/z* ranges at 50–750 and 750–2000, respectively. In this line, calibration of the mass spectrometer for both ionization modes corresponding to the highest m/z range was achieved through the routine commercial calibration solutions provided by the manufacturer. In contrast, customized calibrations were carried out at m/z 50–750 mass range as follows: for negative ionization mode, 87.00877 (pyruvic acid); 117.01624 (fumaric-d2 acid); 149.06471 (glutamic-d3 acid); 208.11399 (Tryptophan-d5 indole); 265.14790 (sodium dodecyl sulfate) and 514.288441 (sodium taurocholate); for positive ionization mode, 74.09643 (n-butylamine), 138.06619 (caffeine fragment), 195.08765 (caffeine) and 524.26496 (MRFA).

The apolar samples were reconstituted in 200 µl of isopropanol/acetonitrile/H_2_O (65:30:5) at the moment of the acquisition. Lipids were separated on a Kinetex® 2.6 µm C18 100 Å column (Phenomenex, Torrance, CA) with the following chromatographic conditions: solvent A, H_2_O/ACN (80:20) with 0.05% FA and 5 mM AA; solvent B, ISP/ACN/H_2_O (90:9:1) with 0.05% FA and 5 mM AA; separation gradient, initially 100% A, linear 5% A in 15 min, purging with 100% A for 2 min and column equilibration with 100% A for 8 min; injection volume, 10 µl; flow rate: 200 µl/min; autosampler temperature, 6 °C; column temperature, 22 °C; total running time, 30 min. The MS device used for the study of lipids was the same as previously described for polar metabolites operating in full-MS negative and positive ionization modes loading the following settings: spray voltage, 3.5 kV; capillary temperature, 250 °C; sheath gas, 25 (arbitrary units); auxiliary gas, 15 (arbitrary units); microscans, 1; AGC target, 1e^6^; maximum injection time, 200 ms; mass resolution, 70,000; scan *m/z* ranges simultaneously acquired, 200–1000 and 150–1000 for negative and positive ionization modes, respectively. Attending to the *m/z* ranges considered for lipids analysis, calibration of the MS system was performed through the commercial calibration solutions from the manufacturer.

The MS detector was calibrated before the analysis of each batch of samples, maintaining at all times a mass tolerance below 5 ppm. Raw MS data from polar samples simultaneously collected in both positive and negative ionization modes was splitted and converted into their respective isolated negative and positive mzXML files by ProteoWizard 3.0^44^. Generated mzXML data were analyzed by XCMS^45^ using the centWave method^46^ with the optimized parameters: ppm = 2.5; peakwidth = c(5,20); snthresh = 4; mzdiff = 0.01; integrate = 1; prefilter = c(3,100) and bw = 5. In the same line, non-polar MS data were also analyzed by XCMS loading the following settings: ppm = 2.5; peakwidth = c(10,60); snthresh = 10; prefilter = c(3,5000) and bw = 5. Furthermore, mzXML files were normalized to the total intensity of all useful features by PQN after IS and weight scaling. The features obtained from XCMS were mined against a database of accurate masses and retention times generated in our laboratory using the same instrumentation and the same conditions used for data acquisition for the 618 small molecule compounds contained in the IROA 300, Mass Spectrometry Metabolite Library of Standards (MSMLS; IROA Technologies, Bolton, MA). In addition, databases of accurate masses taken from Kyoto Encyclopedia of Genes and Genomes (KEGG)^47^, Lipidmaps^48^, Human Metabolome database^40^ and Metlin^49^ by MetaboSearch^50^ were also mined. MS data were then combined to the MRS data for the subsequent post-processing, followed by multivariate analyses and statistical significance analysis.

### Statistical and pathway analyses

Unsupervised multivariate analyses (principal component analysis, PCA) were carried out in PLS-Toolbox (Eigenvector Research, Manson, WA) after log transform. Non-parametric Wilcoxon signed-rank tests were used to evaluate significance between the levels of metabolites during cold exposure (at 2, 4 or 6 hours) and at thermoneutrality. Pathway analysis of polar metabolites from MRS and MS analyses was carried out by MetaboAnalyst 3.0 (http://www.metaboanalyst.ca/MetaboAnalyst/) which is based on the KEGG database (http://www.genome.jp/). Global test algorithm^51^ was applied for significance test of pathways.

### Study approval

All animal husbandry and experiments were carried out in strict accordance to guidelines defined by the Association for Assessment and Accreditation of Laboratory Animal Care and approved by the institutional animal research committees at The University of Texas at Austin.

## Electronic supplementary material

Supplementary Information

## References

[1] Cannon B, Nedergaard J (2004). Brown adipose tissue: function and physiological significance. Physiological reviews.

[2] Villarroya, F., Cereijo, R., Villarroya, J. & Giralt, M. Brown adipose tissue as a secretory organ. *Nature Reviews Endocrinology* (2016). 10.1038/nrendo.2016.136 27616452

[3] Beck V (2007). Polyunsaturated fatty acids activate human uncoupling proteins 1 and 2 in planar lipid bilayers. The FASEB Journal.

[4] Spiegelman BM, Flier JS (1996). Adipogenesis and obesity: rounding out the big picture. Cell.

[5] Wu J, Cohen P, Spiegelman BM (2013). Adaptive thermogenesis in adipocytes: Is beige the new brown?. Genes & development.

[6] Wu J (2012). Beige adipocytes are a distinct type of thermogenic fat cell in mouse and human. Cell.

[7] Feldmann HM, Golozoubova V, Cannon B, Nedergaard J (2009). UCP1 ablation induces obesity and abolishes diet-induced thermogenesis in mice exempt from thermal stress by living at thermoneutrality. Cell metabolism.

[8] Harms M, Seale P (2013). Brown and beige fat: development, function and therapeutic potential. Nature medicine.

[9] Rosell M (2014). Brown and white adipose tissues: intrinsic differences in gene expression and response to cold exposure in mice. American Journal of Physiology-Endocrinology and Metabolism.

[10] Chouchani, E. T. *et al*. Mitochondrial ROS regulate thermogenic energy expenditure and sulfenylation of UCP1. *Nature* (2016). 10.1038/nature17399 PMC5549630 27027295

[11] Barbato, D. L. *et al*. Glutathione decrement drives thermogenic program in adipose cells. *Scientific reports* **5** (2015). 10.1038/srep13091 PMC4531326 26260892

[12] Kell DB, Oliver SG (2016). The metabolome 18 years on: a concept comes of age. Metabolomics.

[13] Griffin, J. L. & Nicholls, A. W. *Metabolomics as a functional genomic tool for understanding lipid dysfunction in diabetes, obesity and related disorders*. (2006). 10.2217/14622416.7.7.1095 17054419

[14] Fiehn O (2001). Combining genomics, metabolome analysis, and biochemical modelling to understand metabolic networks. Comparative and functional genomics.

[15] Tadi S, Sweeney S, Tiziani S (2014). Future Perspectives of Metabolomics in Adipocytes. J Nutrition Health Food Sci.

[16] Bartelt A (2011). Brown adipose tissue activity controls triglyceride clearance. Nature medicine.

[17] Mattila, I., Seppänen-Laakso, T., Suortti, T. & Orešič, M. Application of lipidomics and metabolomics to the study of adipose tissue. *Adipose Tissue Protocols*, 123–130 (2008). 10.1007/978-1-59745-245-8_9 18516557

[18] Merlin J (2016). Could burning fat start with a brite spark? Pharmacological and nutritional ways to promote thermogenesis. Molecular nutrition & food research.

[19] Barbatelli G (2010). The emergence of cold-induced brown adipocytes in mouse white fat depots is determined predominantly by white to brown adipocyte transdifferentiation. American Journal of Physiology-Endocrinology and Metabolism.

[20] Putri M (2015). CD36 is indispensable for thermogenesis under conditions of fasting and cold stress. Biochemical and biophysical research communications.

[21] Albert, V. *et al*. mTORC2 sustains thermogenesis via Akt‐induced glucose uptake and glycolysis in brown adipose tissue. *EMBO molecular medicine*, e201505610 (2016). 10.15252/emmm.201505610 PMC4772955 26772600

[22] Lopez-Soriano F, Alemany M (1987). Effect of cold-temperature exposure and acclimation on amino acid pool changes and enzyme activities of rat brown adipose tissue. Biochimica et Biophysica Acta (BBA)-General Subjects.

[23] Chaneton B (2012). Serine is a natural ligand and allosteric activator of pyruvate kinase M2. Nature.

[24] Tedeschi PM (2013). Contribution of serine, folate and glycine metabolism to the ATP, NADPH and purine requirements of cancer cells. Cell death & disease.

[25] Ye J (2012). Pyruvate kinase M2 promotes de novo serine synthesis to sustain mTORC1 activity and cell proliferation. Proceedings of the National Academy of Sciences.

[26] Hao Q (2015). Transcriptome profiling of brown adipose tissue during cold exposure reveals extensive regulation of glucose metabolism. American Journal of Physiology-Endocrinology and Metabolism.

[27] Plaisier C (2012). Zbtb16 has a role in brown adipocyte bioenergetics. Nutrition & diabetes.

[28] Bettaieb A (2013). Protein tyrosine phosphatase 1B regulates pyruvate kinase M2 tyrosine phosphorylation. Journal of Biological Chemistry.

[29] Yao X, Shan S, Zhang Y, Ying H (2011). Recent progress in the study of brown adipose tissue. Cell & bioscience.

[30] Spasić M (1993). Effect of long-term exposure to cold on the antioxidant defense system in the rat. Free Radical Biology and Medicine.

[31] Chau Y-Y (2014). Visceral and subcutaneous fat have different origins and evidence supports a mesothelial source. Nature cell biology.

[32] Casteilla L, Pénicaud L, Cousin B, Calise D (2008). Choosing an adipose tissue depot for sampling: factors in selection and depot specificity. Methods Mol Biol.

[33] Hwang TL, Shaka AJ (1995). Water Suppression That Works - Excitation Sculpting Using Arbitrary Wave-Forms and Pulsed-Field Gradients. J Magn Reson Ser A.

[34] Gunther UL, Ludwig C, Ruterjans H (2000). NMRLAB-Advanced NMR data processing in matlab. J Magn Reson.

[35] Ludwig, C. & Günther, U. L. MetaboLab - advaced NMR data processing and analysis for metabolomics. *Bmc Bioinformatics* **12** (2011). 10.1186/1471-2105-12-366 PMC3179975 21914187

[36] Dieterle F, Ross A, Schlotterbeck G, Senn H (2006). Probabilistic quotient normalization as robust method to account for dilution of complex biological mixtures. Application in 1H NMR metabonomics. Anal Chem.

[37] Savorani F, Tomasi G, Engelsen SB (2010). icoshift: A versatile tool for the rapid alignment of 1D NMR spectra. Journal of Magnetic Resonance.

[38] Parsons HM, Ludwig C, Gunther UL, Viant MR (2007). Improved classification accuracy in 1- and 2-dimensional NMR metabolomics data using the variance stabilising generalised logarithm transformation. BMC Bioinformatics.

[39] Ludwig CEJ, Lodi A, Tiziani S, Manzoor SE (2012). Birmingham Metabolite Library: a publicly accessible database of 1-D H-1 and 2-D H-1 J-resolved NMR spectra of authentic metabolite standards (BML-NMR). Metabolomics.

[40] Wishart DS (2007). HMDB: the Human Metabolome Database. Nucleic Acids Res.

[41] Matre, P. *et al*. Inhibiting glutaminase in acute myeloid leukemia: metabolic dependency of selected AML subtypes. *Oncotarget* (2016). 10.18632/oncotarget.12944 PMC5340236 27806325

[42] Cramer, S. L. *et al*. Systemic depletion of L-cyst (e) ine with cyst (e) inase increases reactive oxygen species and suppresses tumor growth. *Nature Medicine* (2016). 10.1038/nm.4232 PMC5218918 27869804

[43] Sweeney SR (2016). Metabolomic profiling predicts outcome of rituximab therapy in rheumatoid arthritis. RMD open.

[44] Chambers, M. C. *et al*. A cross-platform toolkit for mass spectrometry and proteomics. *Nat Biotech* **30**, 918–920, doi:10.1038 (2012). 10.1038/nbt.2377 PMC3471674 23051804

[45] Smith CAWEJ, O’Maille G, Abagyan R, Siuzdak G (2006). XCMS: Processing mass spectrometry data for metabolite profiling using nonlinear peak alignment, matching, and identification. Anal. Chem..

[46] Tautenhahn R, Bottcher C, Neumann S (2008). Highly sensitive feature detection for high resolution LC/MS. BMC Bioinformatics.

[47] Okuda S (2008). KEGG Atlas mapping for global analysis of metabolic pathways. Nucleic Acids Res.

[48] Fahy E (2009). Update of the LIPID MAPS comprehensive classification system for lipids. Journal of Lipid Research.

[49] Sana TR, Roark JC, Li X, Waddell K, Fischer SM (2008). Molecular formula and METLIN Personal Metabolite Database matching applied to the identification of compounds generated by LC/TOF-MS. Journal of Biomolecular Techniques: JBT.

[50] Zhou B, Wang J, Ressom HW (2012). MetaboSearch: tool for mass-based metabolite identification using multiple databases. PLoS One.

[51] Xia J, Wishart DS (2010). MetPA: a web-based metabolomics tool for pathway analysis and visualization. Bioinformatics.

