# Supplementary material for: The early metabolomic response of adipose tissue during acute cold exposure in mice
Source: Sci Rep. 2017 Jun 14;7:3455. doi: 10.1038/s41598-017-03108-x (PMC5471228; doi:10.1038/s41598-017-03108-x)
Supplement: Supplementary file 1 — Supplementary Information [file 41598_2017_3108_MOESM1_ESM.pdf]

Supplementary Information

The early metabolomic response of adipose tissue during acute cold exposure in mice

Xiyuan Lu[1#], Ashley Solmonson[2,3,#], Alessia Lodi[1], Sara M. Nowinski[2], Enrique Sentandreu[1], Christopher L. Riley[3], Edward M. Mills[2,3], Stefano Tiziani[1,3*]

[1]Department of Nutritional Sciences & Dell Pediatric Research Institute, The University of Texas at Austin, 1400 Barbara Jordan Blvd., Austin, TX 78723, USA, [2] Division of Pharmacology and Toxicology, College of Pharmacy, The University of Texas, Austin, Texas 78712, USA, [3]Institute for Cellular and Molecular Biology, The University of Texas at Austin, Austin, Texas 78712, USA

[#] These authors contributed equally to this work

*To whom correspondence should be addressed:

Stefano Tiziani, PhD.

Email: tiziani@austin.utexas.edu

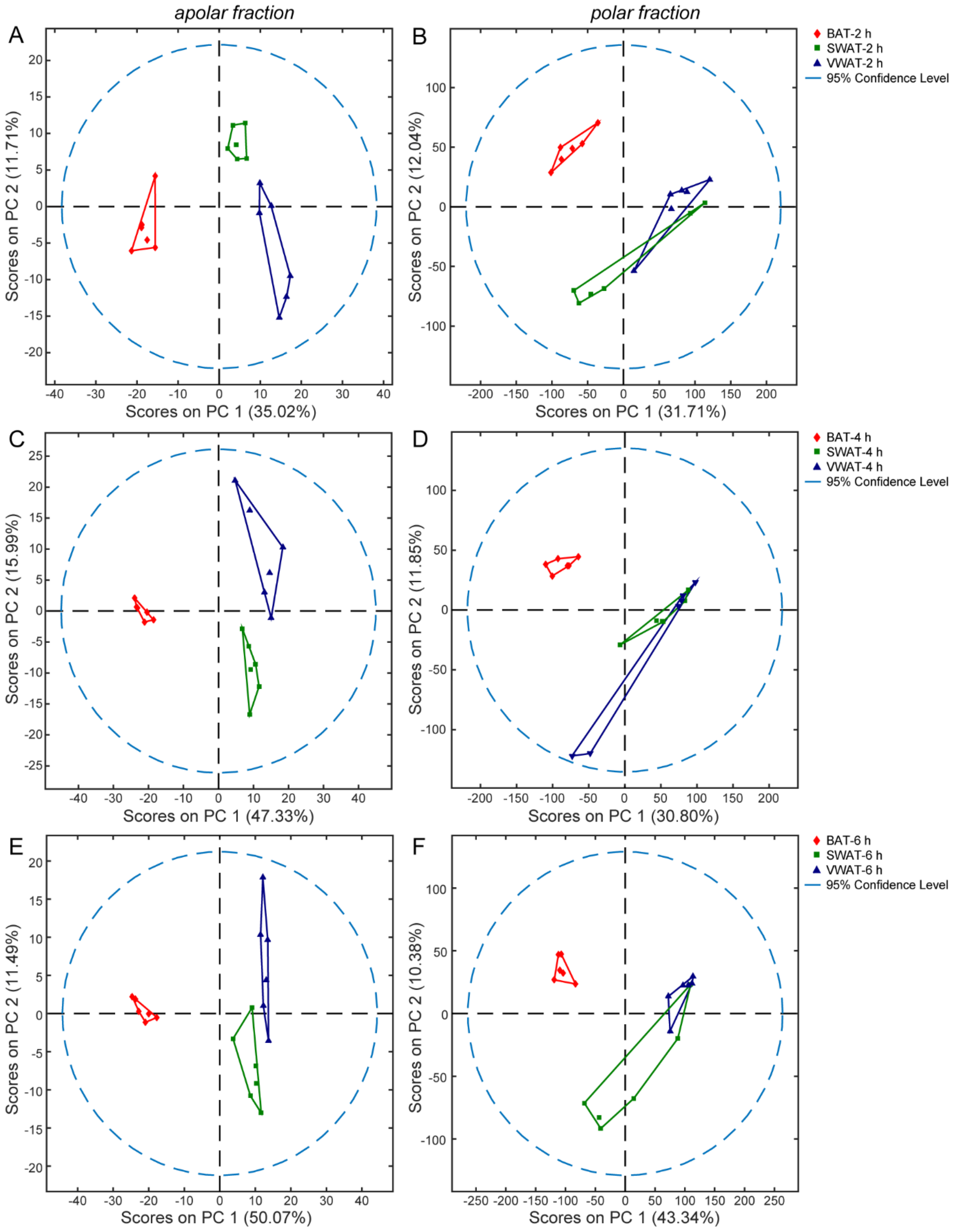

Supplementary Figure 1. Metabolic differences in adipose tissues from different depots following cold exposure. An untargeted multilevel PCA was performed on combined HPLC-MS and MRS data acquired on BAT, SWAT and VWAT samples after 2, 4 and 6 hours of cold exposure. The scores plots obtained from the PCA performed on apolar and polar spectra of BAT, SWAT, and VWAT samples (6 replicates per tissue type) collected after 2 (A and B), 4 (C and D) and 6 (E and F) hours are shown.

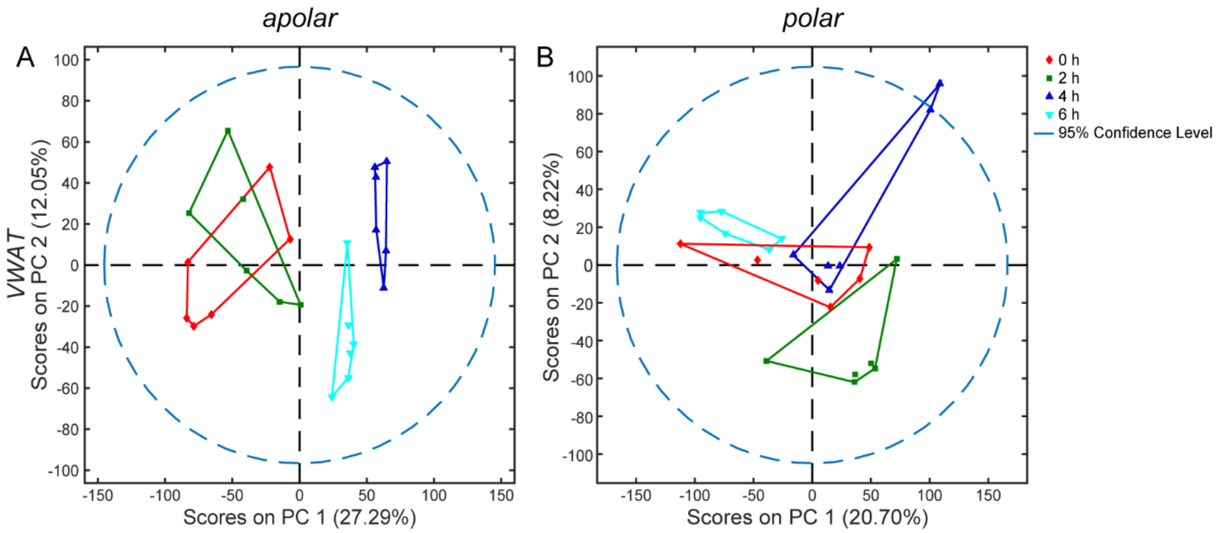

Supplementary Figure 2. Metabolic changes in VWAT during cold exposure. An untargeted multilevel PCA was performed on combined HPLC-MS and MRS data acquired on BAT, SWAT and VWAT samples at thermoneutrality (0 h) and after 2, 4 and 6 hours of cold exposure. The scores plots obtained from PCA performed on apolar and polar spectra acquired on VWAT samples (6 replicates at each time point) collected after 2 (A and B), 4 (C and D) and 6 (E and F) hours are shown.

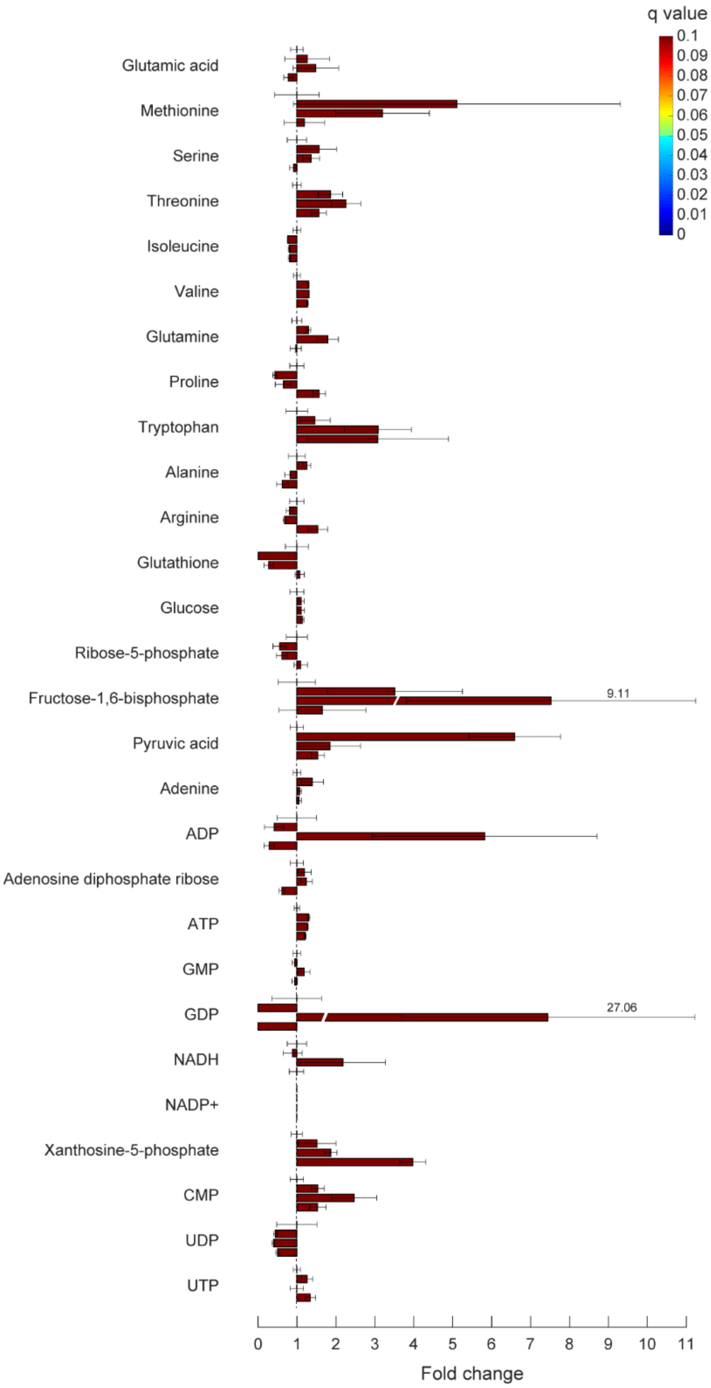

Supplementary Figure 3. Metabolic changes in VWAT samples during cold exposure. Fold changes (compared to thermoneutrality; mean ± standard error, n=6 mice) in selected metabolite levels in VWAT samples collected at 0, 2, 4, and 6 hours. For each metabolite 4 bars are shown: the top bar represent thermoneutrality (always shown at 1 ± standard error), the other (top to bottom) indicate 2, 4 and 6 hours cold-exposure fold change vs thermoneutrality. Statistical significance was calculated by non-parametric Wilcoxon signed-rank tests with FDR. Bars are colored according to q-value.

Supplementary Table 1. Polar metabolite levels in adipose tissues during cold exposure. Fold change (vs thermoneutral) and q values (Wilcoxon signed-rank test with FDR) for metabolites in BAT, SWAT and VWAT following cold-exposure for 2, 4 and 6 hours.

	BAT 0-2		BAT 0-4		BAT 0-6		SW0-2		SW0-4		SW0-6		VW0-2		VW0-4		VW0-6	
metabolite	fc	q	fc	q	fc	q	fc	q	fc	q	fc	q	fc	q	fc	q	fc	q
ATP	1.040	0.148	1.071	0.065	1.137	0.039	0.735	0.140	1.035	0.551	0.969	0.566	1.304	0.142	1.277	0.120	1.219	0.173
NAD+	1.002	0.431	1.014	0.118	1.043	0.122	1.369	0.087	0.973	0.500	0.984	0.621	0.748	0.154	0.773	0.241	0.819	0.349
NADH	0.621	0.052	0.495	0.028	0.627	0.065	0.756	0.434	0.648	0.393	0.668	0.621	0.887	0.423	2.190	0.572	0.993	0.643
NADP+	0.528	0.078	0.098	0.028	0.300	0.039	0.000	0.469	0.000	0.595	2.091	0.684	NaN	0.516	Inf	0.294	NaN	0.643
ADP	0.470	0.052	0.328	0.028	0.629	0.039	0.457	0.411	1.922	0.500	0.853	0.684	0.412	0.423	5.848	0.166	0.285	0.388
Coenzyme A	1.962	0.171	2.135	0.047	1.786	0.399	2.593	0.087	2.495	0.132	2.453	0.307	1.678	0.192	1.297	0.438	1.570	0.451
UDP	0.508	0.148	0.426	0.047	0.522	0.086	0.642	0.074	0.695	0.105	0.643	0.215	0.445	0.232	0.395	0.241	0.501	0.610
FAD	1.060	0.291	0.766	0.334	0.258	0.122	1.158	0.434	0.369	0.268	1.572	0.479	2.079	0.192	1.591	0.294	1.979	0.451
AMP	0.979	0.476	1.001	0.219	1.199	0.260	1.264	0.379	0.885	0.551	1.280	0.479	0.762	0.295	1.251	0.572	0.873	0.409
Pyruvic acid	1.186	0.171	1.374	0.028	1.129	0.157	1.789	0.102	0.384	0.205	1.060	0.678	6.625	0.142	1.853	0.438	1.542	0.289
Acetyl CoA	2.498	0.052	0.331	0.028	6.671	0.039	5.333	0.279	1.764	0.155	1.357	0.430	1.473	0.516	0.767	0.241	1.027	0.610
Glutamic acid	1.559	0.052	1.494	0.047	2.046	0.039	1.619	0.229	1.025	0.595	1.642	0.361	1.268	0.398	1.490	0.572	0.776	0.409
alpha keto glutarate	0.906	0.431	1.524	0.118	0.916	0.399	1.144	0.434	0.620	0.105	0.782	0.479	0.495	0.516	0.510	0.438	0.436	0.643
Glucose	0.995	0.431	1.192	0.028	1.300	0.039	0.576	0.074	1.134	0.393	1.140	0.307	1.112	0.398	1.113	0.484	1.141	0.451
GDP	0.307	0.052	0.038	0.028	0.222	0.039	1.692	0.469	0.858	0.595	0.257	0.543	0.000	0.398	27.061	0.377	0.000	0.496
Oxaloacetate	0.412	0.148	2.449	0.028	1.403	0.122	0.508	0.087	0.313	0.105	0.144	0.155	0.870	0.469	1.630	0.260	1.204	0.496
Glycine	1.060	0.078	1.033	0.151	0.933	0.122	1.284	0.074	1.035	0.155	0.984	0.566	1.139	0.154	1.092	0.260	0.985	0.610
Succinic acid	0.909	0.338	0.859	0.028	0.992	0.399	0.685	0.102	0.820	0.155	0.829	0.361	0.925	0.516	0.846	0.484	1.198	0.289
UDP-N-acetyl-glucosamine	0.806	0.291	0.609	0.151	0.358	0.039	0.929	0.469	0.657	0.132	0.648	0.307	0.824	0.469	1.142	0.526	0.338	0.197
Lysine	0.812	0.108	0.876	0.300	1.728	0.122	0.678	0.175	1.611	0.314	1.383	0.479	0.455	0.192	0.451	0.241	1.658	0.409
Glutathione	0.001	0.052	0.000	0.028	0.031	0.039	0.905	0.434	1.087	0.500	2.953	0.361	0.003	0.142	0.270	0.120	1.073	0.643
CMP	1.472	0.052	1.523	0.028	1.780	0.039	1.854	0.102	1.533	0.132	1.778	0.252	1.540	0.192	2.481	0.120	1.538	0.260
FMN	0.967	0.393	0.944	0.334	1.242	0.306	1.404	0.229	1.263	0.595	1.671	0.430	2.372	0.192	5.107	0.377	1.045	0.643
Arginine	1.037	0.393	0.855	0.219	1.805	0.039	0.660	0.175	1.095	0.551	1.146	0.678	0.811	0.359	0.683	0.241	1.540	0.349
Glutamine	1.153	0.108	1.467	0.028	1.337	0.065	1.065	0.279	1.328	0.105	1.069	0.678	1.300	0.154	1.796	0.120	0.971	0.643
Serine	1.456	0.171	1.868	0.028	1.926	0.039	1.500	0.087	1.400	0.105	1.367	0.307	1.573	0.359	1.368	0.294	0.913	0.552
Methionine	2.087	0.052	2.802	0.028	5.023	0.039	10.698	0.140	9.537	0.105	8.973	0.215	5.132	0.398	3.211	0.260	1.196	0.543
Phosphoenol pyruvic acid	1.197	0.291	0.838	0.219	1.191	0.353	0.822	0.329	1.342	0.500	1.228	0.684	0.697	0.423	0.898	0.572	1.393	0.496
UTP	0.978	0.476	1.749	0.028	1.421	0.086	1.600	0.140	1.606	0.314	1.706	0.155	1.263	0.192	1.001	0.526	1.346	0.173
Ornithine	0.955	0.393	1.257	0.047	1.080	0.260	0.491	0.074	0.822	0.314	0.752	0.307	0.829	0.359	0.897	0.572	1.454	0.260
Phenylalanine	0.854	0.431	0.672	0.300	1.885	0.206	1.029	0.469	1.947	0.205	3.962	0.155	0.642	0.192	0.960	0.526	1.976	0.173
Tyrosine	0.942	0.476	1.147	0.219	1.282	0.086	1.696	0.140	1.445	0.105	1.547	0.155	2.325	0.398	1.423	0.572	0.864	0.496
Malonyl CoA	11.318	0.052	4.673	0.028	21.305	0.039	5.780	0.087	0.605	0.466	4.508	0.155	2.082	0.232	0.719	0.377	2.395	0.173
Fructose-6-phosphate	0.315	0.078	0.497	0.093	0.323	0.065	1.210	0.379	5.436	0.132	2.200	0.215	4.953	0.192	5.167	0.120	4.214	0.173
Succinyl-CoA	1.987	0.108	0.795	0.257	3.284	0.065	1.574	0.329	2.583	0.500	8.084	0.252	3.104	0.142	0.974	0.484	1.334	0.409
Glucose 6-phosphate	0.610	0.052	0.892	0.334	0.743	0.260	1.554	0.329	4.796	0.132	2.674	0.155	3.429	0.142	3.733	0.120	3.426	0.173
sn-glycerol-3-phosphate	1.945	0.052	1.325	0.151	1.202	0.399	0.549	0.102	0.780	0.205	0.771	0.479	0.804	0.423	1.086	0.526	1.030	0.643
UMP	1.019	0.338	0.968	0.219	0.977	0.441	1.223	0.279	1.471	0.551	0.715	0.479	0.894	0.423	0.673	0.241	1.824	0.260
Anthranilic	1.111	0.291	1.290	0.151	2.134	0.039	1.627	0.087	1.468	0.314	1.721	0.215	1.403	0.192	1.688	0.438	1.308	0.409
Dihydroxyacetone phosphate	1.536	0.108	1.502	0.187	1.017	0.441	0.602	0.175	0.496	0.105	0.575	0.252	1.020	0.516	0.924	0.526	1.055	0.643
Choline	1.020	0.291	1.016	0.257	0.954	0.065	1.229	0.102	0.928	0.132	0.982	0.621	0.501	0.142	0.477	0.120	0.517	0.197
Glycerol	0.983	0.338	1.096	0.065	1.238	0.122	0.432	0.087	1.091	0.393	1.086	0.479	1.775	0.142	1.774	0.120	1.818	0.197
Ribose-5-phosphate	0.644	0.228	0.429	0.028	0.311	0.039	0.748	0.469	0.747	0.500	1.008	0.678	0.555	0.232	0.612	0.294	1.101	0.610
L-glyceraldehyde-3-phosphate	2.917	0.078	4.103	0.028	1.489	0.306	0.416	0.074	0.471	0.105	0.430	0.155	0.864	0.423	0.731	0.294	0.669	0.289
Fumarate	2.627	0.148	2.344	0.093	1.299	0.306	0.692	0.279	1.134	0.551	0.810	0.215	0.872	0.295	1.502	0.438	1.006	0.610
Leucine	1.200	0.171	0.937	0.187	0.876	0.260	3.150	0.329	0.829	0.314	0.739	0.307	1.036	0.469	0.632	0.241	0.698	0.496
Oxidized Glutathione	1.065	0.291	1.059	0.151	0.860	0.157	1.825	0.087	1.112	0.500	1.092	0.678	1.730	0.232	1.258	0.260	0.945	0.643
IMP	0.682	0.148	0.901	0.334	0.814	0.260	0.814	0.469	3.398	0.155	1.533	0.678	1.129	0.469	0.971	0.526	0.554	0.173
Histidine	0.904	0.431	0.891	0.257	0.979	0.353	0.602	0.140	1.061	0.466	0.769	0.684	0.799	0.295	0.890	0.438	1.726	0.349
Butyryl-CoA	4.733	0.052	1.879	0.093	6.792	0.039	3.544	0.140	0.695	0.551	2.345	0.252	0.467	0.359	0.413	0.294	0.675	0.451
myo-Inositol	0.953	0.148	0.977	0.187	1.023	0.306	0.494	0.074	1.168	0.105	1.026	0.684	1.421	0.142	1.354	0.120	1.352	0.197
N-acetyl-D-glucosamine	0.641	0.171	0.686	0.093	1.040	0.441	1.849	0.140	1.341	0.393	1.603	0.215	1.538	0.142	4.829	0.120	1.505	0.349
2-keto-isovalerate	1.766	0.171	1.644	0.065	2.351	0.086	0.745	0.229	1.259	0.466	1.507	0.430	0.841	0.469	1.340	0.260	1.291	0.451
GMP	0.502	0.052	0.374	0.028	0.288	0.039	1.199	0.279	0.999	0.595	1.213	0.430	0.945	0.423	1.188	0.572	0.946	0.496
Adenine	2.213	0.052	2.231	0.028	2.738	0.039	1.606	0.102	1.323	0.314	1.530	0.307	1.401	0.232	1.070	0.438	1.052	0.496
Proline	0.330	0.052	0.456	0.028	1.202	0.306	0.509	0.087	1.912	0.205	1.239	0.684	0.431	0.154	0.646	0.294	1.575	0.173
Asparagine	0.771	0.052	1.056	0.257	1.127	0.206	1.670	0.102	1.415	0.132	1.496	0.361	1.152	0.423	1.485	0.260	0.887	0.610
Citrate	0.480	0.291	2.135	0.028	2.683	0.122	0.206	0.087	2.002	0.205	1.020	0.678	0.126	0.142	0.685	0.572	0.794	0.610
Phenylpyruvate	0.906	0.476	0.985	0.334	0.940	0.399	1.212	0.379	1.024	0.551	0.911	0.684	1.026	0.398	0.887	0.526	1.047	0.610
Hydroxypyruvic acid	1.034	0.476	1.241	0.028	1.173	0.206	0.585	0.074	1.644	0.132	0.953	0.678	0.672	0.154	0.851	0.377	2.306	0.289
S-methyl-5-thioadenosine	0.000	0.078	1.039	0.334	0.892	0.399	3.693	0.469	13.885	0.595	0.462	0.684	2.724	0.295	9.148	0.120	31.796	0.173
Thymine	0.064	0.148	1.374	0.300	0.076	0.122	5.859	0.329	1.257	0.466	1.252	0.621	15.288	0.192	7.205	0.438	1.026	0.552
Valine	1.050	0.052	1.052	0.028	1.113	0.039	0.764	0.074	1.019	0.393	1.000	0.684	1.304	0.142	1.314	0.120	1.271	0.173
Lactic acid	0.705	0.052	0.869	0.219	0.850	0.441	0.491	0.074	1.344	0.314	1.004	0.684	0.772	0.154	1.220	0.294	1.733	0.173
Threonine	2.064	0.052	2.232	0.028	2.647	0.039	2.105	0.074	2.095	0.105	2.141	0.155	1.867	0.142	2.270	0.120	1.572	0.173
Glucono-lactone	0.732	0.108	0.963	0.187	1.208	0.157	1.563	0.175	1.366	0.105	1.208	0.252	1.112	0.398	1.470	0.260	0.932	0.496
Adenosine	0.829	0.148	1.684	0.028	0.613	0.086	0.802	0.229	0.918	0.500	1.428	0.215	1.971	0.142	2.813	0.120	3.105	0.173
Sarosine	0.983	0.338	0.977	0.118	1.029	0.441	1.059	0.379	0.972	0.314	0.962	0.479	0.945	0.359	0.963	0.438	0.949	0.496
Thymidine	2.405	0.052	4.117	0.028	1.363	0.157	0.306	0.074	1.115	0.551	1.106	0.684	0.804	0.398	2.594	0.120	1.399	0.409
Glutamate	0.869	0.052	0.884	0.028	0.903	0.039	0.707	0.074	1.024	0.393	0.991	0.684	1.135	0.154	1.238	0.120	1.168	0.197
Guanine	2.948	0.052	5.024	0.028	7.746	0.039	0.814	0.140	0.519	0.132	1.097	0.621	0.951	0.469	0.986	0.572	1.686	0.349
Taurine	0.630	0.052	0.642	0.028	0.527	0.065	0.448	0.074	0.848	0.595	0.822	0.678	0.812	0.142	0.633	0.241	1.187	0.349
Butyric acid	1.134	0.431	1.363	0.187	0.427	0.122	1.131	0.379	1.799	0.595	0.672	0.621	4.177	0.142	0.838	0.484	1.209	0.496
Nicotinate	0.399	0.052	0.720	0.187	0.807	0.399	0.729	0.229	1.582	0.155	1.269	0.684	0.489	0.232	0.438	0.260	0.890	0.610
Riboflavin	7.821	0.052	3.024	0.028	3.797	0.039	15.589	0.175	1.588	0.155	0.468	0.155	3.415	0.423	1.004	0.526	0.780	0.496
Glycerate	1.120	0.431	1.247	0.219	2.034	0.122	0.776	0.279	1.714	0.155	1.341	0.621	0.412	0.142	0.766	0.294	1.578	0.349
Hypoxanthine	0.551	0.171	1.905	0.093	1.299	0.306	1.802	0.087	1.537	0.268	1.346	0.252	1.555	0.142	1.462	0.377	0.883	0.451
D-erythrose-4-phosphate	0.606	0.052	0.811	0.093	0.664	0.039	0.639	0.175	1.007	0.551	0.732	0.361	0.758	0.142	0.707	0.241	1.019	0.610
Inosine	0.409	0.171	1.705	0.093	0.514	0.353	0.996	0.469	1.408	0.393	1.041	0.684	1.160	0.398	2.126	0.294	0.635	0.289
Uridine	0.762	0.148	1.386	0.093	2.503	0.039	1.694	0.102	1.647	0.105	2.615	0.155	1.965	0.142	7.712	0.166	3.255	0.173
Creatine	1.152	0.052	1.231	0.028	1.240	0.039	0.573	0.074	1.111	0.155	1.077	0.430	1.861	0.142	2.477	0.120	1.870	0.173
Adenosine diphosphate ribose	1.263	0.052	1.644	0.047	2.073	0.039	1.168	0.279	1.227	0.393	1.153	0.566	1.193	0.398	1.246	0.260	0.613	0.197
Isocitrate	0.557	0.078	0.714	0.118	1.076	0.441	0.468	0.229	1.091	0.595	1.272	0.684	0.441	0.142	0.468	0.166	1.096	0.643
Spermidine	0.500	0.052	0.272	0.047	0.000	0.039	1.295	0.329	1.187	0.466	1.947	0.307	1.349	0.154	0.589	0.294	0.846	0.552
Citrulline	0.837	0.171	0.927	0.219	0.848	0.206	1.327	0.140	1.688	0.105	1.078	0.621	2.251	0.359	1.580	0.294	0.704	0.289
Kynurenine	1.343	0.108	1.631	0.028	1.750	0.039	1.146	0.279	1.280	0.132	1.010	0.684	1.141	0.398	1.823	0.241	1.025	0.643
Glucosamine	0.827	0.338	0.856	0.187	0.682	0.122	0.854	0.329	0.944	0.595	1.083	0.684	1.166	0.398	0.525	0.241	1.177	0.349
Dihydroorotate	0.312	0.052	0.354	0.028	1.251	0.306	0.379	0.087	2.764	0.314	2.034	0.430	0.280	0.142	0.432	0.166	1.156	0.643
Geranyl-diphosphate	1.600	0.148	1.553	0.093	2.447	0.039	0.915	0.434	0.399	0.155	0.765	0.621	1.033	0.516	0.934	0.572	1.229	0.451
O-Phosphoethanolamine	0.849	0.052	0.855	0.028	0.870	0.039	0.652	0.074	0.994	0.551	1.018	0.479	1.146	0.232	1.527	0.166	1.205	0.197
D-glucosamine-6-phosphate	1.368	0.078	2.080	0.028	2.460	0.039	2.348	0.102	2.159	0.132	4.432	0.155	2.622	0.192	2.550	0.231	2.986	0.173
Fructose-1,6-bisphosphate	0.745	0.338	0.262	0.047	0.819	0.353	1.833	0.363	7.447	0.132	3.275	0.430	3.529	0.423	9.112	0.166	1.659	0.629
3-hydroxy-3-methylglutaryl-Co	0.728	0.431	0.204	0.028	0.677	0.122	10.097	0.087	5.496	0.314	92.663	0.307	5.934	0.142	0.869	0.484	1.913	0.289
d-GDP	0.489	0.052	0.163	0.028	0.502	0.065	0.427	0.469	0.530	0.551	0.246	0.621	0.000	0.259	13.532	0.294	0.000	0.388
Uric acid	0.625	0.171	0.925	0.187	1.010	0.306	0.696	0.140	1.051	0.500	0.990	0.678	0.578	0.142	0.765	0.294	1.188	0.451
Thiamine	0.507	0.078	0.680	0.118	0.606	0.039	1.687	0.140	1.221	0.551	0.849	0.566	0.552	0.232	3.481	0.120	3.087	0.197
Cytosine	1.057	0.393	0.949	0.334	1.328	0.086	1.498	0.074	0.917	0.466	1.833	0.155	1.343	0.516	0.737	0.241	1.120	0.451
Malonate	0.982	0.228	0.976	0.118	0.982	0.306	1.222	0.074	0.953	0.466	0.982	0.621	0.769	0.142	0.772	0.166	0.805	0.197
Xanthine	0.494	0.052	0.848	0.093	0.739	0.039	0.650	0.140	1.157	0.205	0.614	0.307	0.637	0.295	0.653	0.260	0.882	0.643
Guanosine	1.622	0.431	1.071	0.334	1.925	0.157	0.848	0.469	1.018	0.500	1.898	0.479	0.367	0.142	1.573	0.526	1.808	0.643
Aspartic acid	0.953	0.431	1.088	0.118	0.904	0.353	0.610	0.074	0.950	0.500	0.690	0.307	0.748	0.232	0.940	0.572	1.417	0.409
Pipecolic acid	1.225	0.338	1.185	0.334	2.474	0.039	3.182	0.074	1.904	0.105	2.091	0.215	0.835	0.359	0.611	0.241	0.804	0.496
cis-aconitic acid	0.943	0.476	0.950	0.219	0.835	0.086	0.630	0.074	0.751	0.105	0.850	0.479	0.835	0.232	0.721	0.294	1.093	0.496
N-acetyl-L-ornithine	0.649	0.171	0.639	0.093	1.141	0.206	0.531	0.087	0.620	0.268	1.027	0.684	0.697	0.295	0.495	0.260	1.744	0.451
N-carbamoyl-L-aspartate	1.380	0.228	0.431	0.151	1.217	0.306	0.932	0.469	1.264	0.466	1.858	0.155	0.850	0.423	0.617	0.231	10.525	0.643
d-TTP	1.026	0.476	0.788	0.093	0.871	0.260	1.657	0.140	1.165	0.595	1.013	0.684	2.727	0.192	1.347	0.241	0.850	0.496

d-UTP	0.810	0.291	0.838	0.187	0.732	0.039	0.629	0.087	0.650	0.105	0.737	0.361	0.976	0.516	0.757	0.294	1.091	0.451
Cytidine	1.239	0.228	1.575	0.065	1.518	0.086	2.081	0.087	1.180	0.205	1.581	0.430	0.600	0.192	0.851	0.526	0.630	0.289
Cystine	1.325	0.476	2.018	0.028	2.037	0.260	1.979	0.087	0.992	0.551	1.002	0.684	1.499	0.423	0.668	0.260	0.636	0.409
Shikimate	0.889	0.228	0.900	0.300	2.460	0.260	0.803	0.087	1.054	0.466	1.142	0.678	0.866	0.359	0.724	0.241	1.235	0.451
Cysteic acid	0.587	0.052	0.789	0.065	0.623	0.039	0.630	0.074	0.803	0.132	0.729	0.155	0.865	0.154	0.875	0.438	1.157	0.349
Hypotaurine	0.469	0.078	0.834	0.219	1.801	0.065	0.764	0.279	3.429	0.105	2.105	0.684	0.286	0.142	0.970	0.526	1.382	0.289
Tryptophan	0.279	0.052	0.229	0.028	0.774	0.260	1.513	0.279	1.314	0.393	1.054	0.684	1.468	0.359	3.097	0.241	3.090	0.289
Deoxyuridine	1.067	0.431	0.742	0.151	1.455	0.157	1.686	0.074	0.712	0.105	1.382	0.361	0.887	0.398	0.802	0.241	1.572	0.197
Pyridoxamine	1.253	0.228	1.161	0.151	0.584	0.122	1.287	0.469	0.750	0.205	0.899	0.621	0.752	0.295	0.755	0.484	1.301	0.409
CDP-ethanolamine	0.502	0.052	0.563	0.028	0.541	0.039	0.852	0.434	0.453	0.105	0.598	0.479	0.462	0.154	0.719	0.241	0.432	0.197
Guanidoacetic acid	0.541	0.052	0.549	0.028	0.494	0.039	0.789	0.140	0.649	0.105	0.628	0.155	1.120	0.359	1.184	0.260	0.647	0.409
Propylene glycol	1.030	0.148	1.008	0.257	1.041	0.122	1.133	0.087	0.984	0.314	0.974	0.479	0.978	0.398	0.930	0.260	0.937	0.197
O-Phosphocholine	1.073	0.052	1.038	0.028	0.983	0.353	0.966	0.434	0.950	0.155	1.012	0.621	1.027	0.469	0.951	0.377	0.996	0.643
3-phosphoglyceric	1.574	0.228	1.285	0.151	1.199	0.353	0.590	0.087	1.494	0.500	0.685	0.215	0.701	0.154	0.836	0.526	0.853	0.610
Cysteinesulfinate	0.841	0.338	0.791	0.118	1.696	0.065	2.121	0.102	1.294	0.155	1.375	0.361	3.907	0.398	3.987	0.260	1.012	0.610
N-acetyl-glutamate	3.076	0.052	4.290	0.028	5.444	0.039	0.793	0.279	0.994	0.551	1.306	0.479	0.869	0.516	0.700	0.484	1.643	0.289
2-phosphoglyceric	1.310	0.338	1.715	0.065	1.291	0.260	0.244	0.074	1.406	0.314	1.682	0.684	0.099	0.142	0.568	0.294	1.232	0.496
Xanthosine-5-phosphate	3.011	0.078	5.633	0.028	9.918	0.039	2.237	0.140	3.895	0.105	5.236	0.155	1.522	0.423	1.876	0.120	3.993	0.173
sn-Glycero-3-phosphocholine	0.807	0.052	0.877	0.028	0.864	0.039	0.705	0.074	1.028	0.393	0.942	0.307	1.123	0.142	1.465	0.120	1.067	0.409
Malic acid	0.708	0.338	0.599	0.028	0.368	0.039	0.428	0.102	0.615	0.205	0.320	0.215	0.473	0.142	0.857	0.438	0.582	0.289
Cysteine	1.436	0.431	1.922	0.118	2.177	0.353	1.137	0.469	0.252	0.155	2.675	0.566	3.678	0.154	4.330	0.438	79.816	0.173
2-hydroxy-2-methylbutanedioic	0.909	0.393	0.597	0.028	0.771	0.065	1.226	0.102	1.350	0.105	1.341	0.155	1.219	0.232	1.060	0.377	1.201	0.451
D-glucarate	1.090	0.431	0.784	0.219	1.445	0.065	2.932	0.074	2.041	0.205	1.912	0.357	2.286	0.469	1.787	0.526	0.832	0.552
L-Histidinol	0.688	0.078	0.813	0.093	0.236	0.039	0.865	0.279	0.849	0.205	1.158	0.566	0.510	0.192	0.814	0.526	0.971	0.451
Pantothenate	0.572	0.078	0.681	0.028	0.774	0.157	1.314	0.329	0.836	0.314	1.140	0.361	2.593	0.142	1.436	0.166	1.358	0.289
Crotonoyl CoA	1.306	0.171	0.785	0.219	1.424	0.260	1.078	0.379	1.988	0.205	1.261	0.430	1.117	0.423	1.611	0.241	1.086	0.451
Phosphoserine	0.034	0.078	0.000	0.047	0.105	0.086	1.036	0.469	0.362	0.314	1.602	0.252	0.503	0.398	1.425	0.438	5.306	0.260
4-guanidinobutanoic acid	0.779	0.171	0.801	0.187	0.355	0.039	1.024	0.279	2.633	0.268	1.786	0.430	0.614	0.359	0.586	0.294	0.665	0.409
N-acetyl-L-aspartic acid	1.767	0.052	1.684	0.028	1.893	0.065	1.272	0.279	0.931	0.551	1.073	0.678	1.097	0.423	1.673	0.526	0.773	0.289
Thiamine-phosphate	1.413	0.338	2.740	0.028	2.436	0.039	1.915	0.074	1.403	0.500	1.499	0.430	0.952	0.469	1.765	0.526	0.404	0.289
3-Hydroxybutyric acid	3.705	0.052	3.388	0.028	4.846	0.039	1.578	0.074	0.998	0.393	1.626	0.307	1.865	0.423	0.723	0.260	1.072	0.643
(s)-3-hydroxybutanoyl-CoA	0.467	0.171	0.248	0.028	1.507	0.399	3.598	0.074	1.672	0.314	3.106	0.252	2.293	0.192	0.847	0.377	1.414	0.451
2-oxo-4-methylthiobutanoic aci	Inf	0.142	Inf	0.047	Inf	0.441	0.730	0.198	1.859	0.500	2.759	0.357	2.850	0.192	2.838	0.241	5.438	0.197
6-Phosphogluconolactone	0.970	0.431	1.153	0.151	0.883	0.206	0.608	0.140	0.938	0.551	0.803	0.430	0.905	0.295	0.948	0.484	1.286	0.451
Diguanosine tetraphosphate	1.020	0.476	1.516	0.300	0.476	0.206	0.639	0.469	1.738	0.314	0.884	0.678	2.020	0.398	44.055	0.241	0.798	0.552
Alanine	0.709	0.148	1.035	0.187	0.276	0.039	0.798	0.329	0.384	0.132	0.813	0.479	1.257	0.423	0.827	0.526	0.626	0.289
Imidazole	1.024	0.291	1.009	0.334	1.022	0.399	1.308	0.279	0.841	0.205	0.881	0.361	0.490	0.154	0.485	0.166	0.499	0.197
Pyroglutamic acid	1.162	0.148	1.534	0.028	1.539	0.039	1.085	0.329	1.082	0.500	1.104	0.678	1.191	0.359	1.840	0.260	0.948	0.496
2-aminoacrylic acid	0.940	0.291	0.910	0.187	0.926	0.260	0.749	0.087	0.790	0.132	0.845	0.479	0.884	0.516	0.778	0.484	1.186	0.409
2-Aminomuconic acid	0.832	0.431	2.667	0.047	1.358	0.206	0.743	0.379	1.264	0.500	0.682	0.307	1.379	0.359	0.704	0.260	0.830	0.451
Citraconic acid	0.887	0.271	1.056	0.334	0.885	0.206	0.792	0.134	0.867	0.299	0.839	0.397	0.714	0.516	0.689	0.572	0.671	0.643
L-cystathionine	Inf	0.331	Inf	0.134	Inf	0.441	3.624	0.166	3.307	0.155	5.264	0.397	15.252	0.458	3.785	0.241	1.582	0.543
Creatine phosphate	0.966	0.228	0.932	0.093	0.912	0.065	0.930	0.229	1.051	0.393	0.938	0.215	1.133	0.142	1.075	0.294	1.058	0.451
(S)-Allantoin	0.668	0.078	0.919	0.219	0.991	0.441	0.661	0.074	0.670	0.105	1.254	0.566	0.544	0.232	0.777	0.484	1.065	0.552
1-methyladenosine	0.055	0.052	0.139	0.047	0.011	0.039	8.720	0.074	0.699	0.500	5.073	0.155	0.264	0.423	0.175	0.260	0.073	0.289
N-acetylputrescine	1.281	0.476	1.180	0.187	0.703	0.206	1.054	0.434	0.746	0.268	0.753	0.155	0.247	0.142	0.642	0.484	0.293	0.643
Hydroxyisocaproic acid	0.710	0.078	0.814	0.118	0.458	0.039	0.714	0.087	0.741	0.393	0.839	0.479	0.575	0.154	0.560	0.294	0.796	0.610
5-aminoimidazole ribonucleotid	0.814	0.148	0.074	0.028	1.281	0.441	0.583	0.229	0.025	0.105	0.855	0.684	0.046	0.142	0.030	0.120	2.490	0.552
5-l-glutamyl-alanine	5.740	0.052	7.989	0.028	10.755	0.039	1.168	0.469	4.744	0.105	0.980	0.684	2.675	0.142	7.812	0.120	2.976	0.349
Adenylsuccinic acid	3.154	0.052	3.792	0.028	3.141	0.039	4.174	0.102	3.482	0.105	4.456	0.479	1.359	0.516	3.915	0.166	0.637	0.451
5'-phosphoribosyl-N-formylglyc	20.993	0.052	28.150	0.028	46.582	0.039	1.152	0.379	0.416	0.268	1.232	0.678	2.402	0.295	2.378	0.526	1.692	0.451
P1,P4-Bis(5'-xanthosyl) tetraph	34.578	0.078	31.636	0.151	35.859	0.086	2.018	0.102	0.811	0.595	3.460	0.155	3.428	0.154	1.607	0.294	1.622	0.451
N-acetyl-D-glucosamine-1,6-ph	0.483	0.171	0.409	0.187	0.409	0.122	2.600	0.074	1.661	0.205	1.828	0.252	1.196	0.469	3.135	0.166	0.794	0.610
1-(5'-Phosphoribosyl)-5-amino-	4.684	0.148	5.466	0.028	6.932	0.039	Inf	0.317	Inf	0.299	Inf	0.397	Inf	0.259	Inf	0.572	Inf	0.496
Nicotinurate	1.013	0.171	0.994	0.187	1.031	0.399	0.998	0.434	0.992	0.595	1.010	0.479	0.992	0.469	1.076	0.377	0.983	0.496
B-Sulfinylpyruvate	1.880	0.052	2.233	0.028	2.465	0.039	2.185	0.102	1.943	0.105	1.708	0.155	1.817	0.192	2.392	0.120	1.525	0.173
Phenylpropiolic acid	1.061	0.476	1.347	0.151	0.417	0.157	0.556	0.102	0.693	0.205	0.308	0.155	0.940	0.469	0.870	0.294	0.648	0.289
Hydroxyphenylacetic acid	0.761	0.431	0.355	0.028	0.686	0.441	0.780	0.175	0.880	0.595	0.637	0.307	2.336	0.469	0.834	0.377	0.690	0.260
D-4-hydroxy-2-oxoglutarate	1.078	0.393	1.098	0.065	1.271	0.039	1.292	0.175	1.354	0.132	1.115	0.479	1.091	0.359	1.119	0.241	1.025	0.610
Diadenosine triphosphate	1.274	0.171	1.037	0.300	2.785	0.399	1.080	0.434	1.125	0.551	0.936	0.621	0.819	0.423	0.723	0.484	0.625	0.496
Picolinic acid	1.300	0.393	1.658	0.118	2.985	0.157	0.533	0.175	0.941	0.595	1.061	0.684	0.104	0.154	0.870	0.484	0.428	0.451
Acetyllysine	1.777	0.171	2.051	0.047	0.769	0.399	1.783	0.229	1.491	0.500	0.707	0.566	5.354	0.192	3.693	0.294	0.150	0.197
2,3-diketo-5-methylthiopentyl-1	0.419	0.171	0.422	0.300	0.569	0.260	0.318	0.074	1.483	0.595	1.319	0.684	0.137	0.142	0.318	0.572	0.746	0.451
Isoleucine	1.062	0.052	1.059	0.028	1.090	0.039	1.261	0.074	0.937	0.205	0.967	0.566	0.761	0.192	0.804	0.241	0.814	0.289
Homocysteinc acid	3.670	0.052	5.963	0.028	3.566	0.039	4.110	0.074	2.715	0.105	3.598	0.155	12.560	0.142	3.999	0.120	2.772	0.173

Supplementary Table 2. Apolar metabolite levels in adipose tissues during cold exposure. Fold change (vs thermoneutral) and q values (Wilcoxon signed-rank test with FDR) for metabolites in BAT, SWAT and VWAT following cold-exposure for 2, 4 and 6 hours.

	BAT 0-2		BAT 0-4		BAT 0-6		SW0-2		SW0-4		SW0-6		VW0-2		VW0-4		VW0-6	
metabolite	fc	q	fc	q	fc	q	fc	q	fc	q	fc	q	fc	q	fc	q	fc	q
O-butanoyl-carnitine	2.1391	0.1793	1.5879	0.0429	1.2551	0.0736	0.9960	0.2651	0.7317	0.1502	0.9484	0.5951	1.3615	0.1100	0.7292	0.1703	0.8707	0.5604
SFA C15	1.1759	0.1123	1.1363	0.0842	0.9448	0.2008	0.9686	0.2651	0.3907	0.0265	0.7996	0.1939	1.3622	0.0858	0.7082	0.2121	1.0766	0.5604
UFA C16:1	1.2605	0.2896	0.2326	0.0429	0.4064	0.0246	0.6163	0.0878	0.0812	0.0265	0.4571	0.1053	0.5072	0.0858	0.1164	0.0638	0.2732	0.1120
SFA C16	1.3216	0.1793	1.4152	0.2029	1.1035	0.2008	0.6398	0.0685	0.7288	0.0265	0.7437	0.3336	1.3297	0.1369	0.8637	0.3965	1.3138	0.3099
O-hexanoyl-R-carnitine	1.3411	0.0649	0.7445	0.0429	1.0308	0.3095	0.8192	0.0685	0.1781	0.0265	0.4894	0.1053	0.7889	0.1369	0.1721	0.0638	0.4120	0.1120
UFA C17:1	1.8573	0.0318	2.5634	0.0429	2.3060	0.0246	1.4498	0.1120	2.0749	0.0520	1.1190	0.4038	0.6730	0.1100	1.3475	0.2121	0.9140	0.4499
SFA C17	0.4545	0.0493	0.3560	0.0668	0.4633	0.0736	0.7167	0.1120	0.4683	0.0392	0.6050	0.1053	0.6357	0.2501	0.2824	0.0939	0.6534	0.5014
UFA C18:3	2.4771	0.1123	0.7186	0.0429	0.7423	0.1645	0.6236	0.0685	0.1637	0.0265	0.3536	0.1053	0.6358	0.1369	0.2403	0.0638	0.3009	0.1120
UFA C18:2	0.6906	0.0318	95.5951	0.0429	1.8375	0.0246	0.4286	0.0375	6.8325	0.0265	2.3785	0.1053	0.9369	0.2852	12.6144	0.0638	2.0951	0.1120
UFA C18:1	2.1098	0.0649	0.0096	0.0429	0.3309	0.0246	0.5269	0.0375	0.0019	0.0265	0.3223	0.1053	0.4515	0.0505	0.0008	0.0638	0.1941	0.1120
SFA C18	1.3797	0.0891	1.7691	0.0429	2.6169	0.0246	0.1074	0.0375	5.3683	0.0392	4.1909	0.1053	0.7021	0.2501	5.9234	0.1703	1.5878	0.3909
O-octanoyl-R-carnitine	1.1812	0.1430	2.1794	0.0429	1.9197	0.0362	1.5535	0.0375	0.4895	0.0392	0.7664	0.2804	1.2098	0.1674	0.4063	0.0638	0.5670	0.1576
UFA C19:1	1.2467	0.3269	1.4935	0.0429	1.0720	0.2742	0.7360	0.0263	0.4686	0.0265	1.0020	0.5951	1.2178	0.2852	0.5570	0.0638	0.7964	0.4499
SFA C19	1.0760	0.2896	0.4357	0.0842	4.8930	0.2742	0.6210	0.1120	3.1266	0.2663	0.4489	0.1500	0.9023	0.3688	1.0287	0.5426	0.4107	0.1872
UFA C20:5	2.0237	0.0318	1.1788	0.3689	3.4549	0.0504	0.6268	0.0499	0.8306	0.0520	0.5951	0.1939	0.8272	0.2852	1.0293	0.5426	1.0705	0.4499
UFA C20:4	1.6663	0.2512	0.8830	0.1565	0.9470	0.2378	0.5346	0.0263	0.4131	0.0265	0.5642	0.1053	0.4027	0.0858	0.2646	0.0638	0.4418	0.1120
UFA C20:3	1.1613	0.3269	0.7352	0.0668	0.9428	0.2742	0.4962	0.0263	0.4344	0.0265	0.6177	0.1500	0.5168	0.0858	0.3168	0.0638	0.4971	0.1576
UFA C20:2	0.2674	0.0318	0.2658	0.0429	0.6694	0.0246	0.5517	0.0878	0.6248	0.0926	0.8214	0.5017	0.9153	0.3688	0.7763	0.2121	0.8348	0.3099
UFA C20:1	1.8544	0.0891	1.5957	0.1231	2.1317	0.0246	2.3341	0.0263	3.3302	0.0265	2.6003	0.1053	0.4155	0.0505	3.3456	0.3409	0.8364	0.2593
SFA C20	2.1045	0.1430	0.7872	0.3689	0.8977	0.2742	0.6380	0.0685	0.0550	0.0265	0.1458	0.1053	0.5215	0.0505	0.0665	0.0638	0.1916	0.1120
PC(0:0/3:0)_PC(3:0/0:0)_PC(O-1:0,	1.6213	0.1430	0.1481	0.0842	0.4879	0.1645	3.7577	0.0499	0.2429	0.0392	0.1930	0.2547	5.6874	0.0696	0.2051	0.2035	0.2004	0.2480
15-deoxy delta-12,14-prostaglandi	1.6281	0.0493	0.5912	0.1565	0.4096	0.0246	0.7392	0.1120	0.0955	0.0265	0.2226	0.1053	0.8151	0.2123	0.1545	0.0638	0.1962	0.1120
O-decanoyl-R-carnitine	0.7879	0.1793	0.2811	0.0429	0.2844	0.0246	1.6359	0.1436	1.0236	0.2663	0.7031	0.3336	0.7756	0.2852	1.5873	0.5426	0.2606	0.1576
12-HEPE	1.3873	0.1123	0.2513	0.0429	0.2474	0.0246	0.6859	0.1436	0.0661	0.0265	0.2838	0.1053	0.8522	0.2852	0.1026	0.0638	0.2095	0.1120
1-Hexadecylglycerol	1.5058	0.0318	1.2239	0.2029	1.8629	0.0362	1.2855	0.1436	1.1218	0.2340	1.3595	0.3336	0.6851	0.0505	0.8089	0.2121	0.9653	0.5904
12-HETE_15-HETE	1.1599	0.2896	1.8419	0.2029	0.9113	0.3095	0.6777	0.1708	0.0633	0.0265	0.6490	0.4038	0.6364	0.1100	0.0556	0.0638	0.5801	0.1120
UFA C22:6	0.5433	0.2896	0.0073	0.0429	0.2674	0.0504	1.2122	0.2651	0.0103	0.0265	0.6010	0.1939	0.6885	0.1674	0.0175	0.0638	0.5761	0.2076
UFA C22:5	1.0462	0.2512	1.1611	0.2601	1.1939	0.1286	1.1965	0.0878	1.0959	0.1502	1.3421	0.1939	0.9340	0.2501	0.9865	0.5016	0.9863	0.5904
UFA C22:4	0.6647	0.0493	1.0791	0.2601	0.9660	0.2742	0.7525	0.0499	0.5682	0.0265	0.8053	0.4038	0.7504	0.0505	0.5449	0.0638	0.4950	0.1120
MG(16:0/0:0/0:0)	1.3887	0.0649	1.1628	0.1565	1.1867	0.1286	1.1622	0.1708	0.5514	0.0265	0.8881	0.3336	1.4114	0.0696	0.6160	0.0638	0.9008	0.3909
prostaglandin A2_13,14-dihydro-1	3.0123	0.1430	0.8142	0.2601	0.7992	0.1286	1.9649	0.0685	0.6888	0.2340	0.9048	0.5478	1.8169	0.1100	0.4226	0.1703	0.8722	0.5604
UFA C22:3	0.2987	0.0493	6.5379	0.0429	2.2115	0.0504	0.4993	0.1980	3.5878	0.0926	0.1071	0.2547	25.6025	0.0505	15.2975	0.0638	0.3351	0.2076
leukotriene B4	0.2195	0.0318	0.1198	0.0429	0.5614	0.0246	0.4032	0.0685	0.6563	0.1502	0.8468	0.5951	1.0827	0.3688	0.7581	0.4575	0.9380	0.3909
UFA C22:2	1.2798	0.2512	1.2718	0.0842	1.0858	0.1645	0.8284	0.1708	0.5084	0.0265	0.8668	0.5478	0.8715	0.2501	0.8488	0.2731	0.9445	0.5904
UFA C22:1	2.2783	0.0318	0.0783	0.0429	0.4734	0.0246	0.6486	0.0499	0.0491	0.0265	0.4524	0.1053	0.6113	0.0505	0.0426	0.0638	0.2590	0.1120
SFA C22	1.7944	0.1430	0.0679	0.0429	0.5096	0.1286	0.4690	0.1120	0.0708	0.0265	0.0704	0.1053	0.4218	0.0696	0.7585	0.2731	0.1708	0.1120
MG(17:0/0:0/0:0)	1.5843	0.1123	0.0546	0.0429	0.1928	0.0246	0.5312	0.0685	0.0743	0.0392	0.0967	0.1053	0.6348	0.1369	1.6620	0.3409	0.2294	0.1576
prostaglandin D2_prostaglandin E	2.2437	0.0318	0.4229	0.0429	0.7672	0.0972	0.8677	0.1436	0.1923	0.0265	0.5265	0.1053	0.5149	0.0505	0.2532	0.0939	0.3116	0.1120
prostaglandin E1_8-iso-15(R)-pros	0.9861	0.2512	0.9387	0.4152	0.8795	0.1286	0.5076	0.0685	0.2915	0.0265	0.5590	0.2547	0.9219	0.2852	0.7763	0.3965	0.8454	0.5014
MG(18:2(9Z,12Z)/0:0/0:0)	1.7308	0.0493	1.7558	0.2029	1.8267	0.0736	0.7547	0.2651	0.5467	0.0926	0.7870	0.5017	1.0388	0.3688	1.1559	0.5426	1.0715	0.5604
PC(0:0/6:0)_PC(6:0/0:0)	1.6871	0.0891	1.4129	0.2029	1.0949	0.2008	0.7894	0.1708	0.6097	0.0723	0.5957	0.2547	1.4054	0.0505	1.0298	0.5016	0.7676	0.4499
MG(18:1(11E)/0:0/0:0)_MG(18:1(	2.2498	0.0318	2.6612	0.0842	2.8074	0.0362	1.1582	0.1980	0.5202	0.0926	0.7467	0.5017	1.3596	0.1674	0.8026	0.5426	0.6910	0.5904
MG(18:0/0:0/0:0)	0.9099	0.1793	1.1302	0.0429	1.2060	0.0362	0.9850	0.2349	0.7137	0.0265	1.0101	0.4038	1.2508	0.0858	0.7322	0.1703	0.9012	0.4499
Noladin Ether	1.1147	0.1793	0.0711	0.0429	0.5083	0.0972	1.6727	0.1120	0.3109	0.0392	0.1108	0.1053	1.6009	0.1674	2.4100	0.2121	0.4175	0.4499
UFA C24:1	0.4062	0.0318	0.7726	0.0842	1.0522	0.3095	0.7513	0.1436	0.4065	0.0265	1.0947	0.5017	1.5323	0.1674	0.9278	0.3965	2.1821	0.1120
SFA C24	0.6678	0.1123	1.0202	0.4471	1.3363	0.2742	1.1018	0.2651	0.8063	0.1768	1.9787	0.4635	0.7050	0.1100	1.3174	0.3965	0.6857	0.2076
MG(0:0/20:5(5Z,8Z,11Z,14Z,17Z)/(	0.2536	0.0493	0.5836	0.2601	0.2223	0.0362	0.5460	0.0375	0.2443	0.0265	0.1707	0.1500	2.9837	0.0505	3.4935	0.2121	1.8894	0.4499
MG(0:0/20:4(5Z,8Z,11Z,14Z)/0:0)	2.9693	0.0318	1.2161	0.3185	1.6950	0.0504	1.4017	0.0878	1.0465	0.2340	1.5669	0.3336	0.6658	0.1674	1.4904	0.5426	0.7686	0.5604
SFA C25	2.7282	0.0318	1.2469	0.2029	0.9331	0.1286	0.6092	0.1980	0.1731	0.0265	0.3442	0.1053	0.3335	0.0696	0.0717	0.0638	0.2953	0.1120
MG(20:0/0:0/0:0)	9.0316	0.0318	0.2810	0.1231	0.3991	0.0246	0.7388	0.2651	0.0114	0.0265	0.0902	0.1053	0.2351	0.0505	1.0146	0.3409	0.1089	0.1120
15-HETE-G_12-HETE-G	1.5117	0.0649	1.4705	0.0842	1.4999	0.0362	1.3447	0.1120	1.1770	0.1205	0.7675	0.3336	0.7624	0.2123	1.2588	0.0939	0.9504	0.5904
SFA C26	63.3507	0.0318	7.6839	0.0668	31.6255	0.0362	0.3800	0.0375	0.0308	0.0265	0.1049	0.1053	0.3058	0.0505	0.0296	0.0638	0.0549	0.1120
O-hexadecanoyl-R-carnitine	0.2249	0.0493	0.0807	0.0429	0.1790	0.0246	1.6386	0.1120	1.1406	0.2041	1.4746	0.4038	0.4911	0.0505	1.2730	0.5426	0.4241	0.2076
MG(0:0/22:6(4Z,7Z,10Z,13Z,16Z,1	1.4554	0.1430	1.1338	0.3689	1.3011	0.1645	1.0064	0.1436	1.0594	0.2340	1.0030	0.5017	1.5272	0.1100	5.0911	0.1703	1.2113	0.5014
SFA C27	0.7421	0.1123	1.0050	0.4471	0.7373	0.0362	1.3453	0.1436	1.2810	0.2041	1.0957	0.5017	1.6909	0.0858	1.2120	0.4575	0.9902	0.5604
PE(6:0/6:0)	0.7879	0.0493	0.9696	0.3689	0.8663	0.0362	1.0858	0.2349	0.9478	0.2041	1.3568	0.2804	0.6096	0.0858	0.6395	0.0939	1.0446	0.5904
PC(10:0/0:0)_PC(O-8:0/2:0)	0.9273	0.1793	0.7393	0.0429	0.6861	0.0246	1.3002	0.1120	1.0966	0.2340	0.9158	0.5017	1.9222	0.0696	1.0187	0.3409	0.7730	0.3099
LysoPE(0:0/14:1(9Z))_PE(14:1(9Z)/	0.6068	0.0649	0.9007	0.4152	1.1386	0.2008	2.8132	0.0263	1.6769	0.0265	2.0295	0.1939	2.4890	0.0505	3.7119	0.0638	1.9208	0.1120
SFA C28	2.3070	0.0318	0.2659	0.0668	6.1147	0.3095	1.5605	0.1980	0.8217	0.1205	0.9201	0.5478	0.7127	0.1674	2.4889	0.3409	0.5649	0.1872
LysoPE(0:0/14:0)_PE(14:0/0:0)	1.0446	0.2896	1.2536	0.0842	2.7251	0.0246	2.6680	0.0263	1.6854	0.1502	3.2087	0.1053	0.8469	0.3688	0.9526	0.5426	1.8179	0.2076
PC(11:0/0:0)	1.3379	0.1793	0.6065	0.0429	0.7133	0.0362	2.3336	0.0375	0.7076	0.1205	0.6338	0.2804	0.3048	0.1369	0.6688	0.4575	0.1102	0.1872
oleoylcarnitine	0.2692	0.0493	0.1214	0.0429	0.2040	0.0246	1.1722	0.1980	1.1145	0.2041	1.2056	0.5951	0.3955	0.0696	1.2580	0.5426	0.3685	0.1872
O-octadecanoyl-R-carnitine	0.4710	0.1123	0.1715	0.0429	0.3001	0.0504	2.3839	0.0263	1.6975	0.0723	2.1511	0.1500	0.4686	0.0696	3.5143	0.5426	0.4742	0.1872
PE(15:1(9Z)/0:0)	0.8596	0.1123	0.8436	0.2601	2.0532	0.0246	0.5515	0.0499	0.8509	0.1768	1.7951	0.1939	0.2993	0.0696	0.4043	0.0638	0.6557	0.1576
PE(P-16:0/0:0)	0.3591	0.0318	0.6244	0.0429	0.5002	0.0246	0.5059	0.0263	0.6709	0.0392	1.0097	0.5951	0.6540	0.1674	0.5427	0.2731	0.8413	0.4499
PC(O-10:1(9E)/2:0)	4.0548	0.0891	4.7515	0.0429	1.3177	0.2378	3.0822	0.1980	1.7017	0.0723	1.2523	0.5017	1.4199	0.1674	2.3854	0.1703	3.0077	0.2593
LysoPE(0:0/15:0)_PE(15:0/0:0)	0.3314	0.0318	0.3449	0.0429	0.7772	0.0362	0.5750	0.0685	0.7929	0.1768	0.9578	0.5478	0.9472	0.2123	0.7524	0.2121	0.9428	0.4499
PE(O-16:0/0:0)	0.5479	0.1430	0.4626	0.2029	0.9243	0.2008	1.1444	0.1708	1.6336	0.0265	0.9492	0.4635	0.9354	0.3316	1.1733	0.3409	0.9688	0.5604
PC(O-12:0/O-1:0)	1.6046	0.0891	1.3066	0.2029	2.0253	0.0362	4.2158	0.0263	1.7404	0.1205	2.6711	0.2804	0.8598	0.2852	15.4936	0.4575	0.7195	0.5014
PC(6:2(2E,4E)/6:2(2E,4E))	0.7988	0.2186	0.3492	0.0429	0.8704	0.2742	0.3954	0.0685	0.7787	0.1768	0.8195	0.5017	1.5048	0.2501	2.0297	0.2121	1.5896	0.3099
LysoPE(0:0/16:1(9Z))_PE(16:1(9Z)/	1.6452	0.1430	1.6095	0.1565	1.1526	0.2742	0.5052	0.0878	0.6219	0.1502	0.8686	0.4038	0.2571	0.0696	19.6253	0.0939	0.3077	0.2076
SFA C30	0.6777	0.1430	1.1920	0.0429	0.7005	0.0362	0.7107	0.1120	2.2204	0.2663	1.0005	0.5951	0.4855	0.3316	0.6956	0.3409	0.4880	0.4499
PC(O-11:1(10E)/2:0)	1.2470	0.2896	0.5218	0.0429	0.5904	0.0362	1.6033	0.0685	0.9249	0.2041	0.8194	0.5478	1.4917	0.0696	1.3072	0.3409	0.6927	0.5604
LysoPE(0:0/16:0)_PE(16:0/0:0)	2.0519	0.1430	1.2110	0.4471	0.6088	0.0736	0.4332	0.0375	0.0995	0.0265	0.4298	0.1053	0.5772	0.0696	0.2759	0.0638	0.4545	0.1120
PC(O-14:1(1E)/0:0)_PC(P-14:0/0:0	1.6828	0.1430	1.5148	0.2029	2.2629	0.0362	2.3419	0.0375	1.2053	0.2041	1.3367	0.3336	1.4622	0.1100	3.3338	0.2731	0.8800	0.5904
PC(13:0/0:0)	1.0411	0.3269	0.9666	0.4471	1.0902	0.2742	2.3630	0.0263	1.2929	0.0520	1.4122	0.2804	2.1718	0.0505	2.2640	0.0638	1.5621	0.3909
PE(17:2(9Z,12Z)/0:0)	0.9102	0.2186	0.2517	0.0429	0.4690	0.0362	0.5019	0.0263	0.4320	0.0265	0.6227	0.1500	0.4337	0.0505	0.2563	0.0638	0.4790	0.1120
PE(17:1(9Z)/0:0)	0.3600	0.0891	0.5360	0.4471	0.5741	0.2378	1.2204	0.2349	0.7630	0.0520	1.4670	0.1500	1.2793	0.1674	0.6583	0.0939	1.5767	0.3099
PE(O-18:1(9Z)/0:0)_PE(P-18:0/0:0)	1.6459	0.0318	0.5481	0.2601	0.5213	0.0736	0.4764	0.0375	0.0651	0.0265	0.2269	0.1053	0.5859	0.0696	0.1343	0.0638	0.2483	0.1120
PC(14:1(9Z)/0:0)	1.6466	0.2186	0.9045	0.4471	0.9146	0.2742	0.6403	0.1120	0.0680	0.0265	0.1644	0.4635	13.0267	0.0858	2.9316	0.5426	0.3661	0.2076
PC(P-15:0/0:0)	1.1798	0.2512	1.4996	0.1231	2.0550	0.0246	4.4666	0.0263	2.6790	0.0265	2.7652	0.2547	1.0175	0.3316	8.4599	0.1703	0.6489	0.5014
PE(O-18:0/0:0)	3.1576	0.0649	0.4706	0.2601	0.3548	0.1286	0.8659	0.2651	0.0295	0.0265	0.1116	0.1053	0.2769	0.0505	0.0086	0.0638	0.0391	0.1120
PC(14:0/0:0)	1.5417	0.2896	0.9125	0.4152	1.1629	0.1645	2.9298	0.0263	2.0944	0.0392	2.0025	0.2804	1.1202	0.2501	3.5425	0.5016	0.7098	0.2593
PC(O-14:0/O-1:0)_PC(O-15:0/0:0)	1.6352	0.1430	1.4717	0.2029	2.3244	0.0362	1.1262	0.1708	0.5994	0.1768	1.3543	0.1939	1.0242	0.3688	1.1193	0.5016	0.9652	0.5904
LysoPE(0:0/18:4(6Z,9Z,12Z,15Z))_I	1.2851	0.0649	1.5889	0.0429	1.2001	0.0504	0.7951	0.2349	0.7644	0.2340	0.7441	0.5951	0.4786	0.0858	5.6154	0.3965	0.5498	0.1576
LysoPE(0:0/18:3(6Z,9Z,12Z))_Lysol	0.4731	0.0493	0.2920	0.0429	0.3782	0.0246	0.8793	0.1708	0.3469	0.0392	0.9580	0.5017	2.0383	0.0505	0.4592	0.2121	0.9904	0.5604
LysoPE(0:0/18:2(9Z,12Z))_PE(18:2	2.3269	0.1430	1.0614	0.2029	0.8355	0.0246	1.2582	0.2349	0.3634	0.0265	0.9983	0.5478	1.3626	0.2852	0.8713	0.3965	0.9794	0.5904
PE(P-19:1(12Z)/0:0)	0.7579	0.0649	0.6493	0.0429	0.8939	0.2742	0.7780	0.0685	0.5544	0.0265	0.6529	0.1053	0.7900	0.1100	0.7079	0.2121	0.5503	0.1120
LysoPE(0:0/18:1(11Z))_LysoPE(0:0	0.8842	0.1793	0.8498	0.0842	0.8137	0.0246	0.7976	0.0685	0.8757	0.1768	1.1999	0.2804	0.9314	0.2501	0.7486	0.1703	1.1255	0.5604
SFA C32	3.5362	0.0318	0.2870	0.0429	1.3672	0.1645	0.3463	0.0499	0.1031	0.0265	0.2386	0.1053	0.5018	0.0505	0.4506	0.0939	0.3743	0.1576
PC(15:1(9Z)/0:0)	1.3690	0.1123	0.7699	0.4152	1.0465	0.1645	1.7272	0.0375	0.7622	0.1205	0.7942	0.4635	0.5775	0.2852	1.4144	0.3965	0.4155	0.2076
LysoPE(0:0/18:0)_PE(18:0/0:0)	1.1797	0.1123	0.9159	0.3689	1.0426	0.2008	0.7327	0.1708	0.7477	0.2041	0.8329	0.5951	0.8801	0.2123	4.2474	0.1282	0.8565	0.5014
PC(O-16:1(11Z)/0:0)_PC(O-16:1(1E	1.0044	0.3269	0.9088	0.4471	1.3138	0.1286	3.7121	0.0685	0.4900	0.1502	1.9090	0.4038	1.6892	0.0696	10.7871	0.2121	0.6669	0.1120
PC(10:0/4:0)_PC(12:0/2:0)_PC(6:0,	2.1551	0.0493	2.8563	0.1231	1.4911	0.0972	3.1653	0.0375	1.1180	0.2663	0.7777	0.1500	0.7419	0.1100	0.4682	0.0939	0.4503	0.1872
PC(14:0/O-1:0)_PC(15:0/0:0)_PC(7	1.2477	0.0891	1.1836	0.3185	1.7522	0.0362	3.5212	0.0263	1.9533	0.0265	2.1817	0.1939	2.1029	0.0505	2.4789	0.0638	1.5253	0.3099
PC(O-16:0/0:0)_PC(O-8:0/O-8:0)	1.5127	0.1793	1.5140	0.0842	1.9367	0.0362	3.5298	0.0263	2.2173	0.0265	2.2230	0.2804	0.7229	0.1369	4.8499	0.5016	0.6412	0.2593
PE(19:1(9Z)/0:0)	0.7426	0.0891	0.8401	0.0668	0.6160	0.0246	0.7747	0.0685	0.8236	0.1502	0.9112	0.4635	0.6394	0.1100	0.6540	0.1282	0.6737	0.2076
PE(P-20:0/0:0)	1.5194	0.0649	0.9834	0.4471	1.1111	0.3095	1.1068	0.1120	0.7775	0.0926	0.7312	0.1939	0.5531	0.0505	0.7782	0.3409	0.6293	0.1872
SFA C33	0.2953	0.0891	0.2314	0.0429	0.3987	0.0362	0.5015	0.0499	0.9597	0.2340	0.9899	0.5951	0.5189	0.1369	1.2697	0.2731	1.2220	0.2593
PC(16:1(9E)/0:0)_PC(16:1(9Z)/0:0)	1.3990	0.3269	0.8854	0.2601	0.9734	0.2742	2.3492	0.0375	1.8625	0.0265	1.7112	0.3336	1.9036	0.0505	2.6743	0.1282	1.0555	0.5904
PE(19:0/0:0)	0.3948	0.0318	0.5571	0.0429	0.4501	0.0246	0.9811	0.1436	0.3886	0.0392	1.0461	0.5951	2.6379	0.0505	0.4370	0.1703	1.1201	0.5014
PC(P-17:0/0:0)	0.7425	0.3269	0.5061	0.4152	1.1422	0.2742	4.7888	0.0263	1.9688	0.1205	2.6940	0.2804	0.1074	0.0858	1.2910	0.5426	0.0265	0.1576
PE(O-20:0/0:0)	0.7172	0.1430	0.0460	0.0429	0.7032	0.1645	0.7782	0.2651	0.6030	0.1502	1.1513	0.5478	0.6812	0.2501	0.0792	0.0638	0.4942	0.2076
PC(0:0/16:0)_PC(16:0/0:0)_PC(16:	1.1207	0.2512	1.3202	0.1565	1.5710	0.0362	1.9398	0.0263	2.1217	0.0265	1.7720	0.2804	1.4805	0.0696	1.9425	0.2121	1.0587	0.5904
PC(O-16:0/O-1:0)_PC(O-17:0/0:0)	1.8479	0.1123	3.3303	0.0429	7.3427	0.0246	5.6231	0.0263	3.6940	0.1768	0.5034	0.5017	0.8168	0.3688	0.8994	0.5016	0.4494	0.3099
LysoPE(0:0/20:5(5Z,8Z,11Z,14Z,17	1.0876	0.2512	0.6226	0.0429	0.8025	0.0246	0.5719	0.0263	0.5938	0.0265	0.6208	0.1053	0.5279	0.0696	3.8917	0.5426	0.5178	0.1120
LysoPE(0:0/20:4(5Z,8Z,11Z,14Z))_I	0.5286	0.0318	0.9487	0.1231	0.7678	0.0246	0.7754	0.0685	0.8001	0.0723	1.0061	0.5951	0.9050	0.1674	0.8611	0.2731	0.9351	0.5014
PC(8:2(2E,4E)/8:2(2E,4E))	1.1571	0.0891	0.9915	0.4471	0.8876	0.0246	1.0613	0.1980	0.7877	0.0265	0.9595	0.5017	1.3941	0.0505	0.7416	0.1703	0.9094	0.5604

LysoPE(0:0/20:3(11Z,14Z,17Z))_Ly	0.5338	0.0493	1.6616	0.0429	0.7542	0.0362	0.5469	0.0263	0.7722	0.0723	0.9419	0.4635	0.8663	0.2123	0.6880	0.1703	1.0500	0.4499
LysoPE(0:0/20:2(11Z,14Z))_PE(20:	1.6616	0.2186	0.0279	0.0429	0.2798	0.0246	0.6160	0.0499	0.0136	0.0265	0.3464	0.1053	0.4152	0.0696	0.0073	0.0638	0.1636	0.1120
PC(17:2(9Z,12Z)/0:0)	1.3918	0.0891	0.6364	0.0842	1.1051	0.1645	1.9965	0.0375	0.9416	0.2663	1.0694	0.5951	0.6980	0.1369	1.0707	0.5426	0.5278	0.2593
LysoPE(0:0/20:1(11Z))_PE(20:1(11	2.3861	0.0891	0.7199	0.2029	1.5547	0.0972	1.1265	0.2651	0.2885	0.0392	0.8043	0.5951	0.5797	0.1369	0.1719	0.0939	0.4934	0.2076
PC(P-18:1(9Z)/0:0)	1.3245	0.1123	1.0968	0.3185	1.7185	0.0246	2.0305	0.0499	1.4979	0.0723	1.6522	0.3336	0.6556	0.1100	0.9202	0.5426	0.5384	0.1872
SFA C34	0.2857	0.0493	0.3379	0.0668	0.4989	0.0504	0.4892	0.0375	0.6956	0.0926	1.0140	0.5951	0.8485	0.1674	0.4882	0.0939	0.9538	0.5604
PC(17:1(10Z)/0:0)_PC(17:1(9Z)/0:(	1.0972	0.1793	1.3344	0.1565	1.4597	0.0736	1.6347	0.0263	1.6617	0.0265	1.4223	0.2804	0.8778	0.2123	1.4606	0.2121	0.8360	0.5604
LysoPE(0:0/20:0)_PE(20:0/0:0)	0.4200	0.0318	1.0593	0.4152	1.1127	0.2378	0.6716	0.0263	1.3031	0.0392	2.7976	0.1500	0.7482	0.1674	0.9819	0.5426	1.9505	0.1576
PC(O-18:1(11Z)/0:0)_PC(O-18:1(1E	1.6793	0.0493	1.4578	0.1231	2.0738	0.0362	2.8776	0.0263	1.9015	0.0723	2.1167	0.2547	0.4421	0.1100	1.7537	0.5016	0.3993	0.2076
PC(16:0/O-1:0)_PC(17:0/0:0)_PC(	1.5519	0.0649	1.7864	0.0668	2.1319	0.0362	2.2199	0.0263	2.1195	0.0265	1.3578	0.3336	1.2923	0.1100	2.1606	0.2731	0.9276	0.5604
PC(O-16:0/O-2:0)_PC(O-18:0/0:0)	1.5102	0.0891	1.4916	0.1565	1.9379	0.0362	3.3344	0.0263	1.9529	0.0392	1.8433	0.2804	0.6679	0.1674	3.0077	0.5016	0.5376	0.3099
PC(18:4(6Z,9Z,12Z,15Z)/0:0)_PC(1	1.0555	0.3269	0.6520	0.1231	0.8481	0.2008	2.3146	0.0375	1.8038	0.0392	1.5953	0.2804	1.7626	0.0505	3.2318	0.2731	0.9139	0.5604
PC(18:3(6Z,9Z,12Z)/0:0)_PC(18:3(	1.2036	0.2512	0.9375	0.4152	1.2037	0.2008	3.6039	0.0263	3.0265	0.0265	2.6108	0.2547	5.5566	0.0505	7.4196	0.0638	4.2568	0.1120
PC(18:2(2E,4E)/0:0)_PC(18:2(9Z,1	0.8408	0.0318	1.2305	0.1565	1.5195	0.0362	2.2032	0.0263	1.9067	0.0265	1.9301	0.2804	2.2001	0.0505	2.0843	0.0939	1.5302	0.3099
PC(P-19:1(12Z)/0:0)	1.7657	0.0891	0.7885	0.0429	0.7159	0.0362	1.2075	0.1708	0.7911	0.0520	0.8794	0.4038	1.2269	0.2501	0.7826	0.3965	0.9811	0.5904
SFA C35	1.0720	0.2896	1.2125	0.0842	0.9830	0.2008	0.4190	0.0263	1.0708	0.2041	0.7037	0.5951	0.9216	0.3316	0.6940	0.0939	1.2546	0.5604
PC(18:1/0:0)	1.1392	0.1793	0.7807	0.4471	1.2169	0.1645	1.7090	0.0878	1.3922	0.0723	1.1476	0.5478	1.1571	0.2852	2.2649	0.4575	0.6177	0.3909
PE(22:6/0:0)	5.9073	0.0318	1.8418	0.1565	1.6962	0.1645	0.8504	0.1980	3.4514	0.0723	0.2837	0.1053	0.4550	0.0505	0.8542	0.3965	0.4437	0.3099
PC(18:0/0:0)	1.3428	0.0493	1.5534	0.0842	2.0430	0.0246	2.7780	0.0263	2.0432	0.0392	1.8202	0.2547	1.4325	0.0696	2.8315	0.0638	1.2193	0.5014
LysoPE(0:0/22:5(4Z,7Z,10Z,13Z,16	2.7114	0.0318	0.0060	0.0429	0.3306	0.0246	0.6610	0.0685	0.0033	0.0265	0.2596	0.1053	0.4606	0.0505	0.0064	0.0638	0.1807	0.1120
PC(19:3(10Z,13Z,16Z)/0:0)	1.2502	0.1793	1.4345	0.1231	1.8869	0.0362	2.3159	0.0375	2.2663	0.0265	2.0289	0.2804	1.3413	0.1674	2.0752	0.1703	0.9761	0.5904
LysoPE(0:0/22:2(13Z,16Z))_PE(22:	3.6627	0.0318	5.6430	0.0429	15.0174	0.0246	0.5331	0.0685	0.6259	0.1502	0.5168	0.1053	1.5684	0.1674	0.0531	0.0638	0.9274	0.5014
1-linoleoyl-sn-glycero-3-phosphoc	1.3810	0.1123	1.1314	0.3689	1.5406	0.1645	5.5445	0.0263	3.9449	0.0265	2.8879	0.2804	3.3631	0.0505	4.0578	0.0638	1.7088	0.5014
LysoPE(0:0/22:1(13Z))_LysoPE(22:	1.2693	0.1793	0.2524	0.0842	1.8834	0.0504	1.2506	0.0375	1.2057	0.1502	0.8958	0.5478	0.4385	0.1100	1.5528	0.2121	0.7934	0.5014
PC(16:1(9Z)/2:0)	1.2608	0.1793	2.0592	0.0429	2.3705	0.0246	7.3973	0.0263	2.7918	0.0265	3.3505	0.1053	7.0970	0.0505	4.9310	0.0638	2.1991	0.1120
PC(19:1(9Z)/0:0)_PC(O-16:0/3:1(2	1.5601	0.0649	1.3166	0.1565	1.5854	0.0736	1.9782	0.0263	1.6446	0.0392	1.3234	0.4635	0.9106	0.3688	1.4081	0.5016	0.5771	0.3099
PC(O-18:0/O-2:1(1E))_PC(P-20:0/0	2.0064	0.0493	1.1181	0.3185	2.2757	0.0504	4.9464	0.0263	2.1961	0.1502	1.9461	0.3336	0.4003	0.1674	1.2304	0.5426	0.1743	0.3099
PC(16:0/2:0)_PC(2:0/16:0)_PC(9:0,	1.7045	0.1123	0.7660	0.2601	1.1148	0.3095	4.1162	0.0263	1.5199	0.1205	1.6080	0.5017	24.0940	0.0505	9.5308	0.0638	1.7545	0.5014
PC(19:0/0:0)_PC(O-16:0/3:0)_PC(	1.4583	0.2186	1.4935	0.1231	1.7655	0.0362	1.8450	0.0263	1.3958	0.0723	1.1473	0.5017	1.4636	0.1369	1.8861	0.2121	0.9888	0.5904
PC(O-18:0/O-2:0)_PC(O-20:0/0:0)	1.7821	0.0891	1.6678	0.0668	2.5919	0.0246	5.7820	0.0263	3.0065	0.0520	2.3771	0.2804	0.7155	0.2852	1.7020	0.3965	0.6320	0.4499
DG(12:0/18:1(9Z)/0:0)[iso2]_DG(1	3.1895	0.0318	2.9722	0.0429	3.4544	0.0246	1.8022	0.0878	1.4655	0.1205	1.5134	0.2804	2.3915	0.0696	4.6166	0.0638	2.0530	0.1120
DG(12:0/18:0/0:0)[iso2]_DG(13:0/	4.9971	0.0318	1.5153	0.1565	0.9249	0.3095	1.9018	0.0499	1.4533	0.1205	0.4089	0.1053	0.8842	0.3688	1.1477	0.4575	0.4897	0.3909
PC(20:5(5Z,8Z,11Z,14Z,17Z)/0:0)	1.0029	0.2512	1.0526	0.4152	1.7967	0.0736	2.8803	0.0263	2.5454	0.0265	2.7075	0.1500	2.6218	0.0505	13.2200	0.0638	2.2465	0.1120
LysoPC(20:4(8Z,11Z,14Z,17Z))_PC(	0.6899	0.0493	1.2140	0.2601	1.7672	0.0246	2.3806	0.0263	2.4955	0.0265	2.5174	0.1500	1.7416	0.0505	7.0761	0.0638	1.3858	0.3099
LysoPC(20:3(5Z,8Z,11Z))_PC(20:3(	1.0352	0.2896	1.1715	0.3689	1.7204	0.0362	2.2068	0.0375	1.9885	0.0265	1.7522	0.2804	2.1031	0.0696	3.6057	0.0939	1.2344	0.4499
PC(20:2/0:0)	1.5036	0.1793	1.3197	0.1231	1.8964	0.0362	2.9925	0.0263	2.4438	0.0265	2.5545	0.2804	1.2055	0.2123	2.6121	0.3409	1.0389	0.5904
PC(16:0/3:1(2E))	0.9042	0.2186	0.8992	0.4152	0.9975	0.2742	8.4735	0.0263	7.2176	0.0520	4.5851	0.1500	4.8486	0.0505	2.1851	0.0939	1.9967	0.2076
PC(2:0/O-18:1(9Z))_PC(20:1(11Z)/	1.6715	0.0493	1.4176	0.2601	1.9614	0.0736	1.8957	0.0375	1.2739	0.0926	1.1529	0.4038	1.2097	0.2501	2.1659	0.5016	0.7141	0.3909
DG(P-14:0/18:1(9Z))	4.7662	0.0318	3.0708	0.0429	2.6391	0.0246	1.4577	0.0263	1.0679	0.1502	0.7411	0.2547	1.1394	0.2852	1.2513	0.2121	0.8766	0.5904
LysoPE(0:0/24:6(6Z,9Z,12Z,15Z,18	0.4109	0.0318	0.1345	0.0429	0.5213	0.0246	0.4264	0.0263	0.2422	0.0265	0.7431	0.2547	1.7611	0.1369	0.3540	0.1703	0.9453	0.5904
PC(2:0/O-18:0)_PC(20:0/0:0)_PC(	1.9060	0.2512	1.6590	0.0668	2.4739	0.0246	2.1229	0.0878	1.3421	0.1205	1.2711	0.3336	1.4220	0.2501	2.3568	0.3965	1.0284	0.5904
PC(6:2(3E,5E)/14:2(11E,13E))	2.8295	0.0649	3.2010	0.0668	0.5093	0.1645	0.2191	0.0499	3.9284	0.0723	0.2597	0.2547	0.6626	0.0696	0.5576	0.0939	0.0919	0.1120
PC(21:4(6Z,9Z,12Z,15Z)/0:0)	1.4022	0.1793	2.0409	0.0429	2.5422	0.0246	2.3629	0.0263	1.8663	0.0392	2.0230	0.2547	0.4565	0.0858	1.0042	0.5426	0.5355	0.2593
DG(12:0/20:4(5Z,8Z,11Z,14Z)/0:0)	2.6946	0.0318	0.0000	0.0429	0.4141	0.0246	2.7495	0.0263	1.3145	0.0723	1.4750	0.3336	0.7974	0.3316	0.1899	0.1703	0.6463	0.4499
LysoPE(0:0/24:1(15Z))_LysoPE(24:	1.9296	0.0318	1.0411	0.4152	1.4273	0.0362	1.7023	0.0375	0.8725	0.0926	0.8445	0.4038	0.5860	0.0505	0.6123	0.1703	0.6085	0.1120
DG(12:0/20:3(8Z,11Z,14Z)/0:0)[isc	2.7432	0.0318	0.7384	0.2029	0.7081	0.0736	1.4739	0.0685	1.1315	0.1502	0.4537	0.1053	1.0602	0.3688	0.6515	0.3965	0.4548	0.1872
PC(O-18:0/3:1(2E))	2.1610	0.0493	0.9344	0.4471	0.9421	0.2742	1.5792	0.0878	0.8265	0.1502	0.9259	0.5478	1.7903	0.1100	0.9819	0.5016	1.3862	0.3099
LysoPE(0:0/24:0)_LysoPE(24:0/0:0	1.2599	0.2896	1.5962	0.3185	19.1690	0.0246	47.5780	0.0263	5.2412	0.0265	6.0499	0.1053	0.5471	0.1369	1.1281	0.5426	2.3975	0.1872
DG(12:0/20:2(11Z,14Z)/0:0)[iso2]_	3.1709	0.0318	0.9886	0.4471	0.8391	0.2742	1.3153	0.1708	0.8430	0.2041	0.4387	0.1053	1.3446	0.1674	0.9360	0.5426	0.5535	0.3099
PC(21:0/0:0)_PC(O-16:0/5:0)_PC(	1.7656	0.1430	1.4793	0.0842	2.6431	0.0246	4.6938	0.0263	2.0081	0.0926	1.1414	0.5478	0.5631	0.2123	0.9706	0.5426	0.2820	0.3099
DG(12:0/20:1(11Z)/0:0)[iso2]_DG(	3.4209	0.0318	2.0145	0.0842	1.0435	0.2742	2.8055	0.0263	2.7805	0.0265	0.8566	0.5478	1.7573	0.1674	2.4075	0.0638	0.8880	0.5604
PC(22:6(4Z,7Z,10Z,13Z,16Z,19Z)/0	0.8823	0.2186	1.3148	0.2029	1.9340	0.0362	1.8578	0.0685	3.4323	0.0392	2.2164	0.1500	2.6671	0.0505	17.2956	0.0638	1.6907	0.1576
DG(12:0/20:0/0:0)[iso2]_DG(13:0/	4.6869	0.0318	2.7996	0.0429	2.4692	0.0246	1.7376	0.0375	1.3607	0.1502	0.8220	0.5017	1.3083	0.2123	1.5843	0.0939	1.0771	0.5604
LysoPC(22:5(4Z,7Z,10Z,13Z,16Z))_	0.7467	0.0649	1.4651	0.1565	1.9324	0.0246	2.1602	0.0263	3.3357	0.0392	2.4944	0.2547	1.6470	0.0696	3.6677	0.0638	1.3074	0.5014
1-hexadecanoyl-sn--glycero-3-pho	1.5707	0.0891	1.5810	0.1565	1.9444	0.0362	0.7743	0.1436	1.3445	0.1502	1.6799	0.2804	3.8728	0.0696	2.0130	0.0939	3.0307	0.1576
PC(22:4(7Z,10Z,13Z,16Z)/0:0)	1.4583	0.1793	1.7878	0.0668	2.8098	0.0246	3.1683	0.0263	3.1917	0.0265	3.6504	0.1053	0.9110	0.2852	4.4759	0.4575	1.0065	0.5904
PC(22:2(13Z,16Z)/0:0)	2.4990	0.1123	1.0914	0.4152	2.1509	0.0504	2.8696	0.0499	1.3466	0.1205	1.4713	0.5017	0.4092	0.1100	1.4409	0.5426	0.2979	0.2076
PE(12:0/12:0)	1.1217	0.2896	0.4881	0.0842	0.3939	0.0504	0.8397	0.1436	0.7670	0.1768	1.1186	0.5478	0.9913	0.2852	0.5156	0.1282	1.0857	0.5904
LysoPC(22:1(13Z))_PC(22:1(11Z)/0	1.8527	0.0493	1.4559	0.0842	2.4009	0.0362	2.7311	0.0263	1.6692	0.0392	1.5039	0.2804	0.7934	0.2501	1.4366	0.5016	0.5846	0.2593
PC(16:0/5:0)_PC(18:0/3:0)_PC(19:	1.7698	0.2512	0.9635	0.4152	0.9543	0.2742	5.1324	0.0685	1.9073	0.0926	3.1894	0.1053	9.1835	0.0505	3.6035	0.1692	3.8518	0.1120
PC(22:0/0:0)_PC(O-16:0/6:0)_PC(	1.7052	0.0649	1.2982	0.0842	2.4541	0.0246	3.5779	0.0263	2.1368	0.0392	1.6361	0.2804	0.7215	0.2123	1.4508	0.3965	0.5300	0.3099
DG(12:0/22:5(7Z,10Z,13Z,16Z,19Z	2.8138	0.0318	0.6797	0.2601	0.4654	0.0246	1.1897	0.1120	0.7982	0.0926	0.4250	0.1053	1.3615	0.1674	0.5669	0.1282	0.5926	0.2593
DG(12:0/22:4(7Z,10Z,13Z,16Z)/0:(	2.4547	0.0493	1.1065	0.2601	1.1262	0.1645	1.8669	0.0263	1.8846	0.0265	0.7105	0.1500	1.7990	0.1100	1.5465	0.1703	0.8968	0.5014
DG(12:0/22:3(10Z,13Z,16Z)/0:0)[is	3.3087	0.0318	1.0930	0.4152	1.7975	0.0246	1.3513	0.0499	0.9184	0.2340	0.7926	0.3336	1.3391	0.2123	0.7882	0.2121	0.8371	0.4499
PE(12:0/13:0)_PE(13:0/12:0)_PE-N	0.8455	0.1123	1.2297	0.0429	0.9300	0.1286	1.1253	0.1436	1.1742	0.1205	0.8644	0.3336	0.6888	0.1369	0.8701	0.2731	0.6877	0.2076
DG(12:0/22:2(13Z,16Z)/0:0)[iso2]_	3.1390	0.0318	1.5796	0.0668	1.4654	0.0246	1.5236	0.0375	1.0788	0.1502	0.6431	0.1053	1.7100	0.1100	1.5198	0.0939	0.9122	0.5604
PC(11:0/11:0)_PC(16:0/6:0)_PC(4:	1.0890	0.1793	1.4514	0.1231	1.1001	0.2008	0.8673	0.1708	0.8509	0.2041	0.7950	0.4635	1.0165	0.3688	1.2314	0.1282	0.9860	0.5904
DG(12:0/22:1(11Z)/0:0)[iso2]_DG(	4.0194	0.0318	2.8289	0.0429	2.3329	0.0246	1.6525	0.0375	1.2281	0.1502	0.8199	0.4038	1.5916	0.1674	1.4491	0.0939	1.0263	0.5904
DG(12:0/22:0/0:0)[iso2]_DG(13:0/	5.6633	0.0318	3.4669	0.0429	3.6547	0.0246	1.2345	0.1436	0.6723	0.1205	0.7144	0.4038	1.2302	0.2123	1.3239	0.1282	1.1606	0.3909
6-O-(1-O-stearoyl-sn-glycero-3-ph	2.9778	0.0318	0.1295	0.0429	0.5370	0.0736	0.7758	0.1980	0.0196	0.0265	0.1592	0.1053	0.5036	0.0505	0.0701	0.0638	0.1050	0.1120
DG(13:0/22:5(7Z,10Z,13Z,16Z,19Z	0.7914	0.2512	1.0765	0.4152	1.3841	0.2008	1.1637	0.1436	0.7748	0.0723	0.4229	0.1053	1.3716	0.0505	0.6730	0.1703	0.7370	0.2593
DG(13:0/22:4(7Z,10Z,13Z,16Z)/0:(	2.1671	0.2512	3.0358	0.0842	3.8233	0.0504	1.2366	0.1120	1.3819	0.2041	0.5076	0.1053	1.3499	0.0858	0.6869	0.1282	0.9064	0.5014
DG(13:0/22:3(10Z,13Z,16Z)/0:0)[is	1.4134	0.1793	1.2843	0.2601	1.3489	0.1286	1.4017	0.1436	1.3457	0.2663	0.8753	0.2804	0.6908	0.2852	1.5381	0.2731	0.9305	0.5904
PE(12:0/14:0)_PE(13:0/13:0)_PE(1	0.5381	0.0649	0.4729	0.0429	0.5876	0.0504	0.7847	0.1708	0.8585	0.1502	1.5854	0.1053	0.6393	0.1100	1.7316	0.5016	0.9984	0.5904
LysoPC(24:1(15Z))	1.7502	0.0318	1.0393	0.3185	2.1971	0.0246	3.7730	0.0263	2.5362	0.0392	1.7701	0.2804	0.7743	0.2123	1.7768	0.3965	0.5672	0.3909
DG(13:0/22:2(13Z,16Z)/0:0)[iso2]_	4.1267	0.0318	0.8627	0.4152	1.4324	0.0504	1.3844	0.0878	0.7060	0.0520	0.8602	0.5017	1.2975	0.2123	0.8314	0.4575	0.7853	0.4499
PC(24:0/0:0)	1.4555	0.0891	1.4835	0.0429	2.0736	0.0246	3.6639	0.0263	2.7808	0.0265	1.7226	0.2547	0.5261	0.2123	0.9432	0.5426	0.3519	0.3099
DG(14:1(9Z)/22:6(4Z,7Z,10Z,13Z,1	13.5558	0.0318	1.2130	0.4152	0.9859	0.3095	1.5204	0.2651	1.9285	0.0392	0.7964	0.4038	1.2921	0.0858	1.2929	0.3409	0.8661	0.5904
DG(14:0/22:6(4Z,7Z,10Z,13Z,16Z,1	2.2812	0.0891	0.4481	0.0842	0.4135	0.0362	1.2313	0.1980	0.7920	0.0520	0.4979	0.1500	1.4713	0.1100	0.6120	0.2731	0.7064	0.4499
DG(14:0/22:5(7Z,10Z,13Z,16Z,19Z	2.0940	0.0318	0.8204	0.1565	0.7758	0.0972	1.0669	0.1708	0.7670	0.0265	0.7092	0.1053	1.3845	0.0696	0.7774	0.1282	0.8331	0.2593
DG(14:0/22:4(7Z,10Z,13Z,16Z)/0:(	2.3171	0.0318	1.0273	0.4152	1.4019	0.0362	1.1554	0.0878	0.7616	0.0265	0.7140	0.1053	1.5679	0.0505	0.9645	0.3965	0.9102	0.3909
DG(14:0/22:3(10Z,13Z,16Z)/0:0)[is	3.0509	0.0318	1.3945	0.1231	1.9868	0.0246	1.2925	0.0685	0.8604	0.1502	0.6471	0.1053	1.5772	0.0505	1.2173	0.2731	0.8865	0.5604
PE(O-16:0/12:0)	7.3768	0.0318	0.2824	0.2029	1.7327	0.2742	0.3112	0.0685	0.0190	0.0265	0.0210	0.1053	0.8236	0.3316	0.1251	0.0939	0.1198	0.1576
DG(14:0/22:2(13Z,16Z)/0:0)[iso2]_	3.8825	0.0318	2.6857	0.0429	2.5271	0.0246	1.4013	0.0685	0.9632	0.2340	0.7610	0.2547	1.4789	0.1100	1.2533	0.2731	0.9925	0.5904
DG(14:0/22:1(11Z)/0:0)[iso2]_DG(	1.2479	0.1793	1.3522	0.3689	1.7487	0.3095	1.0390	0.2349	0.7306	0.0723	0.8817	0.4635	1.5466	0.0696	1.5728	0.3409	1.7853	0.2593
DG(15:0/22:6(4Z,7Z,10Z,13Z,16Z,1	2.3480	0.0318	0.8197	0.2029	1.4136	0.0246	1.4218	0.0878	1.0542	0.1502	0.6096	0.1053	1.9575	0.0505	1.2370	0.3965	1.0008	0.5014
DG(15:0/22:5(7Z,10Z,13Z,16Z,19Z	3.4455	0.0318	0.9157	0.4152	2.0253	0.0246	1.2738	0.1120	0.8006	0.0926	0.6832	0.1939	1.4889	0.0696	0.7652	0.1703	0.7864	0.3099
DG(18:0/0:0/18:0) (d5)	3.6914	0.0318	2.0330	0.0842	2.2305	0.0246	1.0459	0.2651	0.7873	0.1205	0.7611	0.4038	0.9808	0.3688	1.0754	0.3409	0.7839	0.5014
DG(15:0/22:4(7Z,10Z,13Z,16Z)/0:(	7.4894	0.0318	1.6635	0.2029	4.2899	0.0246	1.9101	0.0878	0.8518	0.2663	0.8481	0.5017	1.2836	0.2852	0.3153	0.0939	0.4952	0.1576
DG(15:0/22:3(10Z,13Z,16Z)/0:0)[is	1.8557	0.1430	0.6722	0.0842	1.4842	0.0362	1.2935	0.0685	0.6993	0.0265	0.9374	0.5478	1.6518	0.0858	0.8700	0.5016	1.1110	0.5604
PE(12:0/16:0)_PE(13:0/15:0)_PE(1	0.5632	0.0891	0.3833	0.0668	1.3823	0.0736	1.1390	0.1708	1.8621	0.0265	1.6467	0.1500	1.2166	0.2501	1.9692	0.0638	1.0688	0.5904
PC(12:0/13:0)_PC(13:0/12:0)_PC(1	1.3083	0.0493	1.7407	0.0429	1.2965	0.0736	1.1578	0.1980	1.1326	0.1502	1.0136	0.5478	1.0685	0.2852	1.2512	0.0638	1.0286	0.5014
DG(18:3(6Z,9Z,12Z)/20:5(5Z,8Z,11	2.8619	0.0891	0.1677	0.0668	0.1104	0.0246	1.7226	0.1120	1.3062	0.0926	0.6598	0.1939	2.7343	0.0505	1.1848	0.3965	1.7003	0.2076
DG(16:1(9Z)/22:6(4Z,7Z,10Z,13Z,1	2.2578	0.0318	0.7353	0.0668	1.0536	0.3095	1.1976	0.1120	0.7868	0.0265	0.6320	0.1053	1.6208	0.0505	0.7683	0.1703	0.8795	0.3099
DG(16:0/22:6(4Z,7Z,10Z,13Z,16Z,1	2.3514	0.0318	0.8573	0.2029	1.3243	0.0246	1.0240	0.2651	0.6742	0.0265	0.6703	0.1053	1.3692	0.0505	0.7109	0.0638	0.7908	0.1576
DG(16:0/22:5(7Z,10Z,13Z,16Z,19Z	2.6966	0.0318	0.9419	0.4152	1.7457	0.0246	1.2411	0.0685	0.7306	0.0265	0.7979	0.1500	1.3106	0.1100	0.6829	0.0638	0.8593	0.3099
DG(16:0/22:4(7Z,10Z,13Z,16Z)/0:(	3.7639	0.0318	1.0564	0.4152	2.3832	0.0246	1.4690	0.0375	0.8208	0.1768	0.9071	0.4038	1.2906	0.2123	0.6586	0.1282	0.7951	0.2076
DG(16:0/22:3(10Z,13Z,16Z)/0:0)[is	4.0511	0.0318	1.4508	0.0842	2.9926	0.0246	1.0411	0.2651	1.6271	0.2041	2.6701	0.2547	0.8059	0.3316	0.8968	0.5016	1.1802	0.4499
PC(10:0/16:1(9Z))_PC(12:0/14:1(9	2.4392	0.0318	0.0000	0.0429	0.9327	0.2742	0.4286	0.0375	0.0001	0.0265	0.4698	0.1500	0.2021	0.0505	0.0001	0.0638	0.1356	0.1120
DG(16:0/22:2(13Z,16Z)/0:0)[iso2]_	0.9818	0.3269	1.8065	0.1231	2.5128	0.0362	1.4061	0.1120	1.2898	0.1205	1.3378	0.4038	1.1427	0.1100	1.1545	0.2121	1.0402	0.5604
PC(10:0/16:0)_PC(12:0/14:0)_PC(1	1.2579	0.0493	1.6066	0.0429	1.2510	0.0972	1.2041	0.1120	1.3339	0.0926	1.1086	0.4038	1.2003	0.1100	1.4454	0.0638	1.0848	0.2593
DG(17:1(9Z)/22:6(4Z,7Z,10Z,13Z,1	3.1011	0.0318	0.1931	0.0429	2.2120	0.0246	1.0927	0.2651	0.6400	0.0520	0.5438	0.1053	1.1438	0.3316	0.6197	0.1282	0.6455	0.1872
DG(17:0/22:6(4Z,7Z,10Z,13Z,16Z,1	3.0760	0.0318	0.9040	0.4152	1.9570	0.0246	1.0669	0.2651	0.5600	0.0265	0.5819	0.1053	1.3299	0.1674	0.5684	0.0638	0.6056	0.1872
DG(17:0/22:5(7Z,10Z,13Z,16Z,19Z	6.5314	0.0318	0.6472	0.3185	4.1541	0.0246	1.5666	0.1120	0.5347	0.0723	0.6432	0.2547	0.9473	0.3688	0.2467	0.0939	0.4687	0.1120
PE(12:0/18:2(9Z,12Z))_PE(13:0/17	0.3586	0.0318	0.7461	0.0429	0.6598	0.0246	1.0054	0.2651	0.9185	0.2340	1.1721	0.4635	0.7203	0.1369	0.9579	0.5426	0.6661	0.2593
DG(17:0/22:4(7Z,10Z,13Z,16Z)/0:(	0.7843	0.1793	0.0992	0.0429	0.5657	0.0246	0.8433	0.1980	1.0517	0.2340	0.8465	0.5951	1.0661	0.3688	1.6166	0.2731	0.9352	0.5604
PE(12:0/18:1(9Z))_PE(13:0/17:1(9	1.8875	0.0891	0.8243	0.3185	0.6472	0.0362	0.8459	0.1436	0.9516	0.2041	1.0783	0.5017	1.0252	0.3688	0.1911	0.0638	0.3221	0.1120
PE(O-16:0/15:1(9Z))_PE(P-16:0/15	3.2864	0.0891	0.6115	0.4471	0.2601	0.1286	0.2615	0.0375	0.4606	0.1768	0.4642	0.4038	14.2531	0.0505	0.5698	0.0939	0.3623	0.1576
PC(12:0/15:1(9Z))_PC(13:0/14:1(9	1.2953	0.2896	0.8960	0.4471	0.6351	0.0972	2.4508	0.1120	1.1016	0.2041	1.0865	0.5478	11.6196	0.0696	11.5165	0.0638	2.0852	0.3099
PE(12:0/18:0)_PE(13:0/17:0)_PE(1	1.0531	0.3269	0.9557	0.3689	1.1059	0.1645	3.1708	0.0263	1.9344	0.0723	1.0855	0.5951	2.0319	0.0696	4.2360	0.0939	0.6976	0.4499

PE(O-16:0/15:0)_PE(O-18:0/13:0)	0.9835	0.2896	0.9543	0.4152	1.1194	0.2378	3.0146	0.0263	2.9537	0.0265	2.6753	0.1053	4.5939	0.0505	7.2321	0.0638	4.6144	0.1120
DG(18:3(6Z,9Z,12Z)/22:6(4Z,7Z,10	1.0592	0.3269	0.5895	0.2029	1.0477	0.2008	1.7551	0.0878	2.8055	0.0392	1.2047	0.5478	3.8453	0.0505	3.4702	0.0638	2.7530	0.2076
PC(12:0/15:0)_PC(13:0/14:0)_PC(1	2.3915	0.1123	0.9316	0.4471	0.6177	0.0736	0.6267	0.1980	0.1627	0.0265	0.2889	0.1053	0.6764	0.3688	0.4902	0.3409	0.6744	0.3909
PC(O-16:0/12:0)	2.2626	0.2186	3.4893	0.0429	3.4452	0.0246	2.5851	0.0878	3.2884	0.0265	1.4854	0.3336	0.3088	0.0696	14.6082	0.0638	0.3360	0.1120
DG(18:2(9Z,12Z)/22:6(4Z,7Z,10Z,1	1.1825	0.2512	1.5191	0.2029	1.1719	0.1645	1.3543	0.1120	1.0530	0.1502	0.7659	0.2547	2.1582	0.0505	1.1127	0.3965	1.2957	0.3099
DG(20:5(5Z,8Z,11Z,14Z,17Z)/0:0/2	1.6540	0.0318	0.6938	0.0842	1.1033	0.2378	1.1833	0.1708	0.9491	0.2340	0.7651	0.2547	1.8668	0.0505	1.0124	0.5426	1.0589	0.4499
DG(18:1(9Z)/22:6(4Z,7Z,10Z,13Z,1	1.6842	0.0318	0.3972	0.0429	1.2077	0.0972	1.1380	0.0375	0.7565	0.0520	0.8083	0.1500	1.0236	0.2852	0.5647	0.0638	0.6562	0.1576
DG(18:0/22:6(4Z,7Z,10Z,13Z,16Z,1	3.6244	0.0318	0.9149	0.4152	2.1517	0.0246	1.1966	0.1436	0.6615	0.0265	0.6806	0.1053	1.0735	0.3316	0.4906	0.1282	0.6109	0.1872
PE(13:0/18:3(6Z,9Z,12Z))_PE(13:0,	0.9286	0.2896	0.7923	0.4471	1.0989	0.1645	1.4047	0.1120	1.3692	0.0723	1.2039	0.5478	1.0081	0.3316	2.5412	0.3965	0.6819	0.4499
DG(20:4(5Z,8Z,11Z,14Z)/0:0/20:4(	3.6391	0.0318	0.8718	0.3689	2.1682	0.0246	1.2372	0.1436	0.6873	0.0265	0.6946	0.1053	1.0548	0.3316	0.4493	0.0638	0.5553	0.1576
DG(18:0/22:5(7Z,10Z,13Z,16Z,19Z	8.4875	0.0318	1.5977	0.1565	6.4519	0.0246	1.8633	0.1120	0.9656	0.2663	0.9515	0.5951	0.8218	0.2852	0.5123	0.0939	0.8954	0.5604
PC(10:0/18:2(9Z,12Z))_PC(14:1(9E	2.1147	0.2512	0.8729	0.3689	1.3279	0.2378	0.4773	0.1120	0.2540	0.0520	0.0956	0.1053	0.2822	0.0505	1.0838	0.5016	0.0742	0.1120
DG(18:0/22:3(10Z,13Z,16Z)/0:0)[is	61.0114	0.0318	7.4753	0.1231	85.9878	0.0246	Inf	0.1630	Inf	0.2663	Inf	0.5951	2.9574	0.3105	0.0000	0.5426	0.0000	0.5904
PC(10:0/18:1(9Z))_PC(12:0/16:1(9	1.6128	0.2186	0.6238	0.1565	0.6386	0.0736	3.2850	0.0878	2.2429	0.0926	1.0294	0.5478	1.5544	0.2501	14.4037	0.3965	0.1920	0.1872
PC(P-16:0/13:0)	1.3906	0.0649	1.6305	0.0842	2.3320	0.0246	0.4234	0.0499	0.3780	0.0520	0.5046	0.2804	0.7910	0.2123	0.4120	0.1282	0.5266	0.3909
PC(10:0/18:0)_PC(11:0/17:0)_PC(1	1.7216	0.1430	1.2456	0.1231	1.1274	0.1645	2.8650	0.0375	2.5618	0.0265	1.5716	0.3336	0.5467	0.1674	3.7286	0.5016	0.3269	0.1872
DG(20:2(11Z,14Z)/0:0/20:2(11Z,14	1.2243	0.2512	0.6769	0.2601	1.2929	0.0972	1.0979	0.2651	0.8091	0.1768	0.9279	0.5017	2.0483	0.1100	2.2808	0.0638	1.6385	0.1576
DG(19:1(9Z)/22:6(4Z,7Z,10Z,13Z,1	0.2589	0.0493	0.4663	0.1231	0.4455	0.0736	0.6098	0.0263	0.4108	0.0265	0.7457	0.1053	0.7431	0.1369	0.3007	0.0638	0.6578	0.2076
DG(19:0/22:6(4Z,7Z,10Z,13Z,16Z,1	0.5679	0.1430	0.4327	0.0429	0.6531	0.0362	0.8726	0.1980	1.0785	0.2041	1.2170	0.2547	1.0480	0.3688	1.5092	0.0939	1.7079	0.1120
PE(12:0/20:3(8Z,11Z,14Z))_PE(14:	0.9128	0.2896	0.8471	0.4471	1.6424	0.1286	2.1491	0.0263	2.3549	0.0520	2.1501	0.2547	0.5535	0.0696	1.5265	0.2121	0.3501	0.1120
DG(19:0/22:5(7Z,10Z,13Z,16Z,19Z	0.0960	0.0318	0.2908	0.0429	0.1391	0.0246	0.6932	0.1436	1.0455	0.2340	1.0871	0.1053	0.8455	0.3316	1.7227	0.0939	1.5267	0.1120
PE(12:0/20:2(11Z,14Z))_PE(14:0/1	1.2794	0.1430	1.3447	0.1565	1.4789	0.0736	1.6445	0.0263	1.6216	0.0392	1.3748	0.3336	0.7789	0.2852	1.3997	0.4575	0.6332	0.3909
DG(20:5(5Z,8Z,11Z,14Z,17Z)/22:6(	2.2218	0.0318	0.2615	0.0429	0.3038	0.0246	1.0351	0.2349	0.9703	0.1768	0.5815	0.1053	3.0318	0.0505	1.2315	0.3965	1.3323	0.3099
DG(19:0/22:4(7Z,10Z,13Z,16Z)/0:C	0.5858	0.0891	0.0562	0.0429	0.5067	0.0972	0.9777	0.2349	1.2632	0.1768	0.8232	0.5017	0.8899	0.3688	1.2443	0.3965	0.9133	0.4499
PC(12:0/17:2(9Z,12Z))_PC(14:1(9Z	1.4640	0.1123	1.1419	0.3185	0.9972	0.3095	1.4132	0.1120	1.3114	0.0926	1.1864	0.5951	1.8351	0.0505	1.8911	0.0638	1.0105	0.5604
PE(12:0/20:1(11Z))_PE(13:0/19:1(	0.3454	0.0318	0.9024	0.3689	0.4828	0.0246	2.5516	0.0263	3.6387	0.0265	4.0995	0.1500	5.4671	0.0505	2.6673	0.0939	4.1066	0.1120
PE(O-16:0/17:1(9Z))_PE(O-18:0/15	0.4446	0.1430	0.1001	0.0668	0.4773	0.1645	1.1511	0.2349	0.1334	0.0690	1.1998	0.5951	15.1403	0.0505	0.8698	0.5426	1.2806	0.5604
PC(P-16:0/14:1(9Z))	1.8120	0.2423	2.6601	0.4152	0.6081	0.1645	0.8892	0.2651	0.5128	0.1205	0.2731	0.2804	11.2465	0.0505	7.9367	0.0638	4.3976	0.1120
DG(20:4(5Z,8Z,11Z,14Z)/22:6(4Z,7	1.5246	0.0318	0.2262	0.0429	0.3872	0.0362	1.4159	0.0685	1.4281	0.0520	0.7281	0.1500	2.6208	0.0696	2.1527	0.0638	1.3953	0.3099
PC(12:0/17:1(9Z))_PC(13:0/16:1(9	1.9934	0.1123	1.3099	0.2029	0.6476	0.0736	2.3164	0.0375	1.5388	0.0926	0.9074	0.5017	1.9332	0.0858	1.7751	0.0939	1.0335	0.5904
PE(12:0/20:0)_PE(13:0/19:0)_PE(1	0.8462	0.0318	0.5829	0.0429	0.9807	0.1645	1.6654	0.0375	1.2511	0.1502	1.1829	0.4635	1.4060	0.0696	2.1460	0.2731	0.9587	0.5604
PE(O-16:0/17:0)_PE(O-18:0/15:0)_	0.3196	0.1123	0.4812	0.2601	1.1014	0.3095	2.6302	0.0878	3.5847	0.0265	1.6228	0.5478	0.6876	0.2123	0.9525	0.5016	2.2703	0.1576
PC(O-14:0/16:1(9Z))_PC(O-16:0/14	2.3843	0.0318	1.6869	0.0668	2.4459	0.0246	1.1298	0.1708	0.5857	0.1502	0.5375	0.5017	0.3351	0.0858	3.6062	0.5016	0.1990	0.1872
PE-NMe2(O-16:0/O-16:0)	0.5035	0.1430	0.1363	0.0429	0.9959	0.2378	1.0506	0.2349	1.7167	0.0723	1.4108	0.3336	0.6618	0.1674	1.0688	0.4575	1.5917	0.3099
DG(20:3(8Z,11Z,14Z)/22:6(4Z,7Z,1	1.6341	0.0318	0.1884	0.0429	0.6780	0.0736	1.1120	0.1980	0.9730	0.2663	1.0358	0.5951	2.0352	0.1100	1.9257	0.0939	1.7675	0.2593
PC(10:0/19:0)_PC(12:0/17:0)_PC(1	1.7484	0.0891	1.4576	0.0668	1.4889	0.0736	3.7753	0.0375	2.8192	0.0392	1.9071	0.3336	0.3407	0.1100	1.4703	0.5016	0.2151	0.2076
PC(O-14:0/16:0)_PC(O-16:0/14:0)_	2.5554	0.0318	1.4608	0.0429	1.9771	0.0362	3.2969	0.0263	1.2304	0.1768	1.3970	0.3336	0.2725	0.1100	0.8567	0.5426	0.1701	0.2076
DG(20:2(11Z,14Z)/22:6(4Z,7Z,10Z,	3.5882	0.0318	0.6736	0.2601	1.7332	0.2008	1.2842	0.1120	0.6290	0.1768	0.8395	0.5951	1.7711	0.0858	0.8943	0.5016	0.9799	0.5604
DG(20:1(11Z)/22:6(4Z,7Z,10Z,13Z,	0.1056	0.0493	0.4447	0.1231	0.1057	0.0362	0.4650	0.0499	0.7214	0.0265	0.6379	0.1500	1.2698	0.2852	1.4514	0.1703	1.3186	0.3909
PE(13:0/20:4(5Z,8Z,11Z,14Z))_PE(	0.5648	0.0318	1.2331	0.2601	1.6590	0.0362	2.0318	0.0263	2.5394	0.0265	2.6787	0.1500	1.5046	0.0696	7.0658	0.0638	1.5121	0.2076
PE(O-16:0/18:4(6Z,9Z,12Z,15Z))_P	0.9754	0.2896	1.0610	0.4152	1.4208	0.0246	2.0830	0.0263	1.9410	0.0392	1.7274	0.1939	1.1188	0.2123	2.0019	0.0638	1.2484	0.5014
DG(20:0/22:6(4Z,7Z,10Z,13Z,16Z,1	1.7452	0.0318	0.5259	0.0429	1.1704	0.2008	0.7863	0.1708	1.0855	0.2663	0.9046	0.5017	0.9093	0.3688	1.7233	0.0638	1.3998	0.2076
PC(12:0/18:4(6Z,9Z,12Z,15Z))_PC(	1.8962	0.2186	0.9845	0.4471	0.5362	0.0972	0.7320	0.2431	7.2946	0.0392	0.4787	0.5656	2.2257	0.2773	78.3746	0.0638	0.4815	0.4428
PC(12:0/18:3(6Z,9Z,12Z))_PC(12:0,	1.6945	0.1123	0.9606	0.4152	0.9690	0.3095	4.2400	0.0263	3.7331	0.0265	1.6201	0.4635	0.5376	0.1674	5.9210	0.3409	0.2352	0.1872
PE(O-16:0/18:2(9Z,12Z))_PE(P-16:	0.9890	0.3269	1.0529	0.3689	1.3287	0.0362	1.7303	0.0263	1.1622	0.1768	1.4549	0.2804	1.7900	0.1369	2.4848	0.0638	0.9877	0.5904
PC(12:0/18:2(9Z,12Z))_PC(13:0/17	1.8058	0.2186	1.0156	0.4152	0.9493	0.2008	3.5494	0.0375	6.0901	0.0265	1.9017	0.3336	2.1351	0.0505	2.6563	0.0939	0.7212	0.5014
PE(13:0/20:1(11Z))_PE(14:0/19:1(	4.3789	0.0318	2.0069	0.0842	2.7435	0.0246	4.5182	0.0263	2.1704	0.0520	0.6523	0.5017	3.0069	0.0505	33.6496	0.2731	1.2561	0.4499
PE(O-16:0/18:1(9Z))_PE(O-18:0/16	1.8365	0.0891	0.7728	0.2601	2.2704	0.1645	4.2549	0.0263	2.0020	0.0265	1.3701	0.4635	0.4131	0.1369	0.6282	0.2121	0.4044	0.2593
PC(12:0/18:1(9Z))_PC(13:0/17:1(9	1.6565	0.2186	1.0012	0.3689	1.0380	0.2742	2.6935	0.0375	3.3651	0.0265	1.7105	0.3336	0.7883	0.2501	6.8154	0.3409	0.4272	0.1872
PE(12:0/21:0)_PE(13:0/20:0)_PE(1	1.4660	0.1430	7.1490	0.0429	1.8610	0.0736	0.7943	0.2651	0.7601	0.1768	0.7974	0.5478	0.6366	0.1100	61.1870	0.2731	1.0684	0.5014
PE(O-16:0/18:0)_PE(O-18:0/16:0)_	1.5785	0.0891	5.8461	0.0429	2.0901	0.0246	2.5009	0.0263	2.0614	0.0265	1.8812	0.2547	6.4025	0.0696	1.3622	0.5426	0.7619	0.3099
PC(O-16:0/15:1(9Z))_PC(P-16:0/15	1.0119	0.2896	1.0749	0.3185	1.4149	0.1286	1.6285	0.1436	0.9328	0.2663	1.2196	0.5017	0.2177	0.0858	0.4210	0.1703	0.2851	0.2076
PE(12:0/22:6(4Z,7Z,10Z,13Z,16Z,1	2.4402	0.0649	0.6457	0.1231	0.4752	0.0972	1.5680	0.0263	0.3463	0.0265	0.1664	0.1053	1.4676	0.1100	0.3833	0.0638	0.2560	0.1120
PC(10:0/20:0)_PC(11:0/19:0)_PC(1	2.1507	0.0318	1.5302	0.0429	1.3805	0.0736	3.0256	0.0263	1.8897	0.0392	1.4628	0.2804	0.6413	0.2501	1.2878	0.5016	0.4051	0.2076
PC(O-16:0/15:0)_PC(O-18:0/13:0)	2.1982	0.0493	0.7319	0.2601	2.1255	0.0504	2.9524	0.0685	0.4683	0.1768	1.0048	0.5951	0.2648	0.1100	0.1053	0.1703	0.1318	0.2076
PE(14:0/20:5(5Z,8Z,11Z,14Z,17Z))_	0.3353	0.0891	1.4220	0.0668	0.9476	0.2378	0.7677	0.1894	3.1671	0.0265	1.4999	0.5951	1.6975	0.2501	3.5801	0.0939	3.6810	0.2076
PE(12:0/22:4(7Z,10Z,13Z,16Z))_PE	1.3271	0.1430	1.1967	0.2601	1.0574	0.2742	0.5654	0.0263	0.4927	0.0265	0.8661	0.4038	0.8672	0.1369	0.3765	0.0939	1.1662	0.3909
PC(13:0/18:4(6Z,9Z,12Z,15Z))_PC(	1.7696	0.0493	0.9724	0.4471	1.3061	0.0736	1.6637	0.1120	1.4876	0.0926	1.4392	0.4038	1.5795	0.1100	4.3524	0.0939	0.8626	0.5904
DG(22:6(4Z,7Z,10Z,13Z,16Z,19Z)/2	1.9498	0.0493	0.1492	0.0429	2.7804	0.0246	0.8448	0.1120	0.6295	0.0265	0.7539	0.2804	0.9639	0.3316	0.6877	0.1282	0.9239	0.5014
DG(21:0/22:5(7Z,10Z,13Z,16Z,19Z	0.1418	0.0318	0.2917	0.0429	0.1761	0.0362	0.6742	0.0685	0.9645	0.2663	0.9407	0.5017	0.6227	0.1369	1.2285	0.2121	1.1243	0.3909
PC(13:0/18:3(6Z,9Z,12Z))_PC(13:0,	1.4993	0.0649	1.0984	0.2601	0.8904	0.1645	1.2057	0.1708	1.0721	0.1502	0.7739	0.4038	1.6743	0.0505	1.6236	0.0638	0.9257	0.5904
PE(12:0/22:2(13Z,16Z))_PE(14:0/2	0.3469	0.0318	0.9837	0.4152	1.0743	0.2008	0.7491	0.0685	1.5362	0.0265	2.8244	0.1053	0.6100	0.1100	1.2082	0.4575	2.2793	0.1120
PE(O-18:0/17:2(9Z,12Z))_PE(P-16:	0.5202	0.0318	0.4817	0.0429	0.6318	0.0972	1.5921	0.0878	1.5369	0.0926	1.2939	0.4635	1.1289	0.2501	1.9224	0.3965	0.6056	0.3099
PC(O-14:0/18:3(9Z,12Z,15Z))	1.3547	0.1793	1.2048	0.2029	2.0402	0.0246	5.7944	0.0263	3.4647	0.0265	2.0127	0.4038	0.9318	0.3688	8.8647	0.1703	0.5026	0.3099
DG(22:5(7Z,10Z,13Z,16Z,19Z)/22:6	1.0528	0.3269	0.0101	0.0429	1.6668	0.0246	0.8153	0.1980	0.7312	0.1205	0.9138	0.5478	0.8359	0.1674	0.5180	0.0638	0.8677	0.3099
PC(13:0/18:2(9Z,12Z))_PC(14:0/17	2.1502	0.0318	1.8587	0.0429	1.9358	0.0246	1.7152	0.0263	1.4102	0.0520	1.0130	0.5017	2.0148	0.0505	1.6973	0.0638	1.2204	0.3099
PE(12:0/22:1(11Z))_PE(14:0/20:1(	2.5351	0.0493	0.0000	0.0429	0.0328	0.0246	0.2284	0.0263	0.0000	0.0265	0.1057	0.1053	0.4376	0.0858	0.0000	0.0638	0.1476	0.1120
PC(14:0/P-18:1(11Z))_PC(14:0/P-1	1.5534	0.0891	1.5832	0.1231	2.3346	0.0246	2.2619	0.0685	1.5030	0.1502	1.1239	0.4038	0.5431	0.1369	2.2352	0.5016	0.3178	0.2076
DG(22:4(7Z,10Z,13Z,16Z)/22:6(4Z,	5.1726	0.0493	0.1006	0.0668	1.4200	0.1645	1.4289	0.1436	0.5851	0.0265	0.6247	0.1500	2.3860	0.1674	0.9118	0.3965	0.8365	0.3909
PC(12:0/19:1(9Z))_PC(13:0/18:1(9	2.0704	0.0493	1.4307	0.0668	1.1460	0.1286	1.7322	0.0263	1.1275	0.1768	1.0777	0.5478	1.6309	0.0858	1.3017	0.1703	1.0233	0.5904
PE(12:0/22:0)_PE(13:0/21:0)_PE(1	0.6393	0.1793	0.5513	0.2601	0.4768	0.1286	0.7011	0.2349	0.2743	0.0723	0.1495	0.2804	0.4022	0.0505	0.3135	0.0638	0.2129	0.1872
PC(14:0/P-18:0)_PC(16:0/P-16:0)_	2.2497	0.0318	1.4224	0.0668	1.8957	0.0246	2.3694	0.0263	1.2567	0.1205	1.0791	0.5478	0.2757	0.1100	0.9080	0.5426	0.1701	0.2076
PE(O-18:0/O-18:0)_PE(O-20:0/O-1	0.6388	0.1123	0.4008	0.0429	0.6226	0.0246	2.2878	0.0499	1.3166	0.1502	1.1748	0.5951	1.5018	0.0858	5.1655	0.5426	0.8394	0.5904
PE(P-16:0/20:5(5Z,8Z,11Z,14Z,17Z	0.2112	0.0318	0.5307	0.0668	0.2382	0.0246	0.5322	0.1120	0.3712	0.0723	1.3820	0.2547	1.8330	0.0858	0.4093	0.2731	0.8194	0.5014
PC(10:0/21:0)_PC(11:0/20:0)_PC(1	2.9624	0.0318	1.4028	0.0668	2.0789	0.0362	3.2705	0.0263	1.3065	0.1502	1.4527	0.3336	0.7509	0.2501	0.6953	0.3409	0.4665	0.2076
PC(16:0/O-16:0)_PC(O-14:0/18:0)_	1.6755	0.0493	1.0487	0.4471	1.3741	0.0246	3.7167	0.0263	1.2767	0.1502	1.5777	0.2804	0.5259	0.1674	0.5090	0.3409	0.3060	0.2076
DG(22:2(13Z,16Z)/22:6(4Z,7Z,10Z,	0.6571	0.0318	0.8867	0.4152	0.8760	0.1286	1.5294	0.0375	1.2851	0.0265	1.2859	0.4635	1.8363	0.0505	2.6944	0.0638	1.5486	0.1120
PE(O-16:0/20:5(5Z,8Z,11Z,14Z,17Z	0.7363	0.0318	0.9961	0.4471	0.8751	0.0972	0.7927	0.0685	0.8384	0.1205	1.4489	0.1500	0.9237	0.2501	0.7460	0.1703	1.2706	0.3099
PC(12:0/20:5(5Z,8Z,11Z,14Z,17Z))_	3.0424	0.2186	1.2159	0.0842	0.9735	0.3095	1.4392	0.1436	2.1604	0.0392	0.9877	0.5017	0.9048	0.2501	3.9039	0.0638	1.2640	0.3909
PE(O-16:0/20:4(5Z,8Z,11Z,14Z))_P	0.7917	0.2186	2.3186	0.1231	1.8276	0.1286	2.0261	0.0263	5.4922	0.0265	2.0714	0.1053	1.8558	0.0505	3.5248	0.0638	1.7513	0.1576
DG(22:0/22:6(4Z,7Z,10Z,13Z,16Z,1	2.0090	0.0649	0.2879	0.0429	2.1232	0.0246	0.6368	0.0263	0.9229	0.1768	1.0593	0.4038	0.6070	0.0858	1.3628	0.0939	1.2405	0.1120
PC(12:0/20:4(5Z,8Z,11Z,14Z))_PC(	1.4252	0.1793	0.9980	0.4471	0.7876	0.1286	4.5746	0.0375	5.5218	0.0265	1.8200	0.4038	0.8127	0.3688	5.5790	0.2731	0.2816	0.2076
DG(22:0/22:5(7Z,10Z,13Z,16Z,19Z	5.4866	0.0318	0.8569	0.4471	3.5669	0.0246	1.0716	0.1980	0.5049	0.0520	0.8016	0.4635	0.1932	0.0505	0.2902	0.0939	0.2036	0.1576
PC(12:0/20:3(8Z,11Z,14Z))_PC(14:	2.4455	0.1123	1.1795	0.3185	1.1619	0.1286	3.2595	0.0263	6.9685	0.0265	2.5616	0.2547	2.0194	0.0858	3.1143	0.0638	1.0514	0.5904
PE(13:0/22:2(13Z,16Z))_PE(15:0/2	0.8491	0.1793	1.4122	0.1231	1.7683	0.0362	2.4503	0.0263	2.2354	0.0265	2.4944	0.1500	2.2283	0.0505	3.7657	0.0638	2.0917	0.1576
PC(P-16:0/17:2(9Z,12Z))	0.8038	0.2896	13.6603	0.0429	3.8679	0.0246	0.6248	0.1708	30.8323	0.0265	17.7977	0.1053	0.1751	0.0505	0.9430	0.4575	1.0059	0.3909
PC(12:0/20:2(11Z,14Z))_PC(14:0/1	1.8048	0.0318	1.3537	0.0429	1.6450	0.0362	2.8851	0.0263	2.8104	0.0265	1.5152	0.2804	1.2621	0.2123	3.0428	0.0638	0.6967	0.3909
PE(13:0/22:1(11Z))_PE(14:1(9Z)/2	0.1504	0.0493	0.8521	0.4152	0.8129	0.3095	0.2779	0.0685	0.1349	0.0392	0.4645	0.1500	21.2630	0.0505	1.8296	0.1282	3.5586	0.2076
PC(18:1(9Z)/P-16:0)_PC(O-16:0/17	1.1286	0.2896	1.1220	0.2601	1.4971	0.0246	1.3704	0.1436	3.3402	0.0723	1.9333	0.2547	0.4040	0.1100	0.7455	0.3965	0.4992	0.2076
PE(O-16:0/20:1(11Z))_PE(O-18:0/1	0.3110	0.0318	0.8276	0.2029	1.0527	0.2378	0.3724	0.0685	1.0099	0.1768	1.2816	0.3336	0.4540	0.0696	1.8714	0.0638	1.2942	0.1120
PC(12:0/20:1(11Z))_PC(13:0/19:1(	2.1554	0.0318	1.6343	0.0429	1.5003	0.0362	2.5906	0.0263	2.1227	0.0265	1.2593	0.3336	1.0224	0.3688	1.9404	0.3409	0.5414	0.2076
PE(13:0/22:0)_PE(14:0/21:0)_PE(1	2.2096	0.0649	0.8305	0.4471	1.0002	0.2742	1.3975	0.0375	0.2897	0.0392	0.0829	0.1053	2.2345	0.0505	0.4068	0.0638	0.1318	0.1120
PE(O-16:0/20:0)_PE(O-18:0/18:0)_	0.2306	0.0318	0.1409	0.0429	0.2777	0.0246	0.3636	0.0263	0.5795	0.0392	0.5474	0.1053	0.7490	0.2501	2.0850	0.0638	1.9512	0.1120
PC(O-16:0/17:1(9Z))_PC(O-18:0/15	2.6625	0.0318	1.2211	0.1565	1.0353	0.2742	2.1106	0.0499	1.0161	0.2041	1.1564	0.5478	0.2762	0.1100	0.2319	0.1703	0.1505	0.2076
PE(14:0/22:6(4Z,7Z,10Z,13Z,16Z,1	0.5936	0.0318	0.5834	0.0429	0.5296	0.0246	0.5013	0.0685	1.0190	0.1768	1.1206	0.4635	0.3654	0.1100	0.8425	0.3965	0.9722	0.5904
PC(10:0/22:0)_PC(11:0/21:0)_PC(1	1.9315	0.0318	1.3360	0.0668	1.8214	0.0246	2.5909	0.0263	1.1366	0.1768	1.2064	0.3336	0.9052	0.3316	0.8142	0.3965	0.5743	0.3099
PC(O-16:0/O-18:0)	1.5807	0.0318	0.6442	0.0842	0.9533	0.1645	5.4551	0.0263	1.3175	0.2041	0.7686	0.5478	2.2727	0.0696	2.5738	0.3965	0.2718	0.5014
PE(14:1(9Z)/22:4(7Z,10Z,13Z,16Z))	3.1083	0.0318	2.7344	0.0429	2.2944	0.0246	1.1247	0.1980	0.9427	0.2663	0.7333	0.1939	1.0829	0.3316	1.2151	0.2121	0.9350	0.5604
PC(13:0/20:5(5Z,8Z,11Z,14Z,17Z))_	1.2867	0.0649	0.9046	0.3185	1.1624	0.1286	1.0794	0.1980	0.8228	0.0926	0.6131	0.2547	1.8799	0.0505	1.6476	0.1703	1.1701	0.5904
PE(14:0/22:4(7Z,10Z,13Z,16Z))_PE	0.4985	0.0891	0.3881	0.1231	0.4717	0.0972	0.9459	0.2349	1.5139	0.1205	1.1984	0.4635	0.8291	0.1674	1.4326	0.1703	1.4737	0.1120
PC(18:4(6Z,9Z,12Z,15Z)/P-16:0)_P	0.1952	0.0318	0.2808	0.0668	0.3456	0.0736	1.4938	0.1120	2.6746	0.0723	1.2364	0.5017	5.6083	0.0505	7.0309	0.0638	3.8996	0.1120
PC(13:0/20:4(5Z,8Z,11Z,14Z))_PC(	2.1264	0.0318	1.4396	0.0429	1.8892	0.0246	1.8152	0.0263	1.3323	0.0392	1.0404	0.5478	1.5248	0.0696	2.1206	0.1703	0.8428	0.3909
PE(14:1(9Z)/22:2(13Z,16Z))_PE(16	0.7230	0.0891	1.2619	0.0842	0.7611	0.0972	0.7765	0.0499	1.0293	0.2041	1.3344	0.1500	0.9971	0.3688	1.1281	0.2731	1.2597	0.3099
PC(18:3(6Z,9Z,12Z)/P-16:0)_PC(18	1.6570	0.0493	1.6955	0.0842	3.4348	0.0246	2.6640	0.0263	1.6049	0.0723	1.3440	0.5017	0.6051	0.2123	31.0756	0.3965	0.3691	0.2076
PE(14:0/22:2(13Z,16Z))_PE(14:1(9	1.7348	0.1123	0.6340	0.0429	0.2380	0.0246	1.4957	0.0375	0.2780	0.0265	0.2132	0.1053	1.4851	0.0858	0.4130	0.0638	0.2365	0.1120
PC(13:0/20:3(8Z,11Z,14Z))_PC(15:	1.6600	0.0649	1.4446	0.0429	1.7011	0.0246	1.1983	0.1980	1.0106	0.2663	0.8417	0.3336	1.8569	0.0505	1.3482	0.1703	1.0631	0.5014
PC(16:1(9Z)/P-18:1(11Z))_PC(16:1	2.1768	0.0891	1.6790	0.0429	2.0635	0.0246	2.8371	0.0685	1.4068	0.1768	0.9857	0.5951	0.5287	0.2123	3.7701	0.4575	0.3131	0.2076
PC(13:0/20:2(11Z,14Z))_PC(14:1(9	1.7594	0.0318	1.6053	0.0429	1.7673	0.0246	1.8950	0.0263	1.5177	0.0392	1.1588	0.3336	2.0734	0.0505	1.8182	0.0638	1.3335	0.2076
PE(14:0/22:1(11Z))_PE(14:1(9Z)/2	1.0048	0.3269	0.8678	0.2601	1.0572	0.2742	4.1536	0.0263	1.4276	0.1502	2.8394	0.1939	3.8153	0.0696	2.6326	0.0939	2.6573	0.1872
PC(16:0/P-18:1(11Z))_PC(16:0/P-1	1.4583	0.0318	1.0704	0.4152	2.3508	0.0246	1.9161	0.0375	1.0051	0.2340	0.9902	0.5478	0.7690	0.1674	0.7139	0.3965	0.4577	0.2076

PC(13:0/20:1(11Z))_PC(14:0/19:1(	1.7939	0.0318	1.6926	0.0429	2.0184	0.0246	2.0778	0.0263	1.7501	0.0265	1.2221	0.3336	1.2833	0.1674	1.9453	0.2121	0.8746	0.4499
PE(14:0/22:0)_PE(15:0/21:0)_PE(1	0.8055	0.3269	1.9125	0.0429	0.9658	0.3095	5.0101	0.1436	1.1062	0.1768	1.0680	0.5017	14.0378	0.0505	9.6144	0.4394	0.4432	0.5904
PC(O-16:0/18:1(9Z))_PC(O-16:1(9Z	1.6227	0.0318	1.2205	0.0429	1.5311	0.0246	2.5025	0.0263	1.4356	0.0723	1.3778	0.3336	0.3977	0.1100	0.6162	0.3409	0.2641	0.2076
PE(O-16:0/21:0)_PE(O-18:0/19:0)_	0.6708	0.1430	5.0593	0.0429	1.3476	0.1286	7.6086	0.0263	5.9321	0.0265	6.5934	0.2547	2.1803	0.1369	1.5975	0.3409	0.5353	0.3099
PE(15:0/22:6(4Z,7Z,10Z,13Z,16Z,1	0.2690	0.0318	0.3401	0.0668	0.3118	0.0362	0.6256	0.0375	1.2186	0.1502	1.2470	0.1053	0.9814	0.3316	0.8105	0.5016	0.7677	0.3099
PE(O-16:0/22:6(4Z,7Z,10Z,13Z,16Z	0.7547	0.3269	5.5629	0.0429	1.1679	0.2378	3.0915	0.1630	1.6866	0.1712	1.6270	0.5656	0.0000	0.3688	4.1188	0.2416	3.7646	0.4428
PC(10:0/23:0)_PC(11:0/22:0)_PC(1	1.6905	0.1430	1.2356	0.2029	1.1872	0.1645	3.7555	0.0263	1.1969	0.1502	1.2802	0.4635	0.7447	0.3316	0.2873	0.1703	0.4336	0.3909
PC(18:0/O-16:0)_PC(O-16:0/18:0)_	1.2210	0.1430	1.0520	0.3689	1.5382	0.0362	3.7485	0.0499	1.1947	0.2340	1.3843	0.5017	0.6532	0.1369	0.9577	0.5426	0.5564	0.3909
PC(12:0/22:6(4Z,7Z,10Z,13Z,16Z,1	2.5123	0.0891	1.3461	0.0668	1.6030	0.1286	11.2217	0.0263	23.6876	0.0265	3.6985	0.1053	2.5988	0.2501	11.2061	0.0638	0.6845	0.5014
PE(O-16:0/22:5(4Z,7Z,10Z,13Z,16Z	1.9375	0.0318	0.0122	0.0429	0.1894	0.0246	0.2789	0.0263	0.0051	0.0265	0.2181	0.1053	0.6340	0.1100	0.0248	0.0638	0.2139	0.1120
PC(14:0/20:5(5Z,8Z,11Z,14Z,17Z))_	1.9211	0.0318	1.1825	0.0668	1.3547	0.0246	3.5526	0.0263	3.2457	0.0265	1.3860	0.3336	1.2165	0.2852	2.3096	0.2121	0.5300	0.2076
PE(15:0/22:4(7Z,10Z,13Z,16Z))_PE	5.5936	0.0318	2.5916	0.0429	2.4945	0.0246	0.8144	0.1708	0.5865	0.1205	0.3988	0.1053	0.6925	0.1674	0.7101	0.3409	0.5052	0.2076
PE(O-16:0/22:4(7Z,10Z,13Z,16Z))_	2.2321	0.0493	0.0000	0.0429	0.0533	0.0246	0.2019	0.0263	0.0000	0.0265	0.1288	0.1053	0.6062	0.1674	0.0210	0.0638	0.1283	0.1120
PC(12:0/22:4(7Z,10Z,13Z,16Z))_PC	1.4156	0.1793	1.0160	0.4471	1.7113	0.0504	2.5601	0.0263	3.8422	0.0265	1.8894	0.1939	1.3288	0.2123	8.0321	0.1282	0.6535	0.3099
PE(15:1(9Z)/22:2(13Z,16Z))_PE(17	2.3353	0.1793	17.0616	0.0429	0.2201	0.0362	1.3318	0.1436	0.7623	0.0926	0.6502	0.2547	3.2149	0.0696	2.0640	0.1703	0.9517	0.5904
PC(O-15:0/20:4(5Z,8Z,11Z,14Z))	1.4194	0.0318	1.4213	0.1565	1.8018	0.0246	1.8741	0.1708	1.0045	0.1502	1.3693	0.5478	0.1750	0.1100	0.7497	0.4575	0.1601	0.1576
PC(14:0/20:3(5Z,8Z,11Z))_PC(14:0,	0.9339	0.3269	1.5192	0.1565	0.8303	0.1645	1.7802	0.1120	1.7026	0.1768	0.9321	0.5478	0.9072	0.3316	1.4280	0.3409	0.6873	0.3909
PE(O-16:0/22:2(13Z,16Z))_PE(O-18	0.7946	0.0891	0.0032	0.0429	0.5608	0.0246	0.4360	0.0375	0.0085	0.0265	0.7624	0.1053	0.4084	0.0505	0.0021	0.0638	0.5592	0.3099
PC(12:0/22:2(13Z,16Z))_PC(14:0/2	2.5658	0.0318	1.8369	0.0429	2.0824	0.0246	2.0110	0.0263	1.4210	0.0392	0.9088	0.5017	1.6718	0.0696	1.3203	0.0638	0.9309	0.5904
PE(15:0/22:1(11Z))_PE(15:1(9Z)/2	0.8888	0.2896	1.0524	0.4471	2.2593	0.0246	2.6185	0.0263	2.6549	0.0265	3.3353	0.1939	2.3268	0.2501	2.4551	0.4575	0.7627	0.4499
PC(18:1(9Z)/P-18:0)_PC(O-18:0/17	0.8815	0.2186	1.4570	0.1565	2.2474	0.1286	1.2515	0.1708	0.9436	0.2340	0.5156	0.1939	5.4892	0.0696	5.8450	0.0638	1.8237	0.1872
PE(16:1(9Z)/22:6(4Z,7Z,10Z,13Z,16	0.5736	0.1430	0.3411	0.0842	0.7513	0.2742	2.4043	0.0685	1.4149	0.0723	0.9082	0.5478	1.8278	0.1674	1.2121	0.3965	1.1504	0.5014
PC(12:0/22:1(11Z))_PC(14:0/20:1(	2.2498	0.0318	1.6132	0.0429	1.7786	0.0246	1.9431	0.0263	1.3602	0.0392	1.0029	0.5478	1.3106	0.1674	1.4176	0.4575	0.7352	0.3099
PE(15:0/22:0)_PE(16:0/21:0)_PE(1	0.8094	0.2896	12.0621	0.0429	1.2005	0.2008	4.1917	0.0685	7.8277	0.0392	4.2737	0.1500	349.1941	0.0505	207.7153	0.0638	10.9833	0.1120
PC(O-16:0/19:1(9Z))_PC(O-17:0/18	1.7934	0.0318	0.8681	0.3689	1.4273	0.0362	1.8246	0.0685	1.6851	0.0265	0.8437	0.3336	2.8857	0.0505	3.9979	0.0638	1.3371	0.3099
PE(16:0/22:6(4Z,7Z,10Z,13Z,16Z,1	0.4205	0.0318	0.6889	0.0668	0.8601	0.0972	0.3827	0.0263	0.9203	0.1768	1.0207	0.5478	0.4203	0.0505	1.2270	0.2121	1.4612	0.1120
PC(10:0/24:0)_PC(11:0/23:0)_PC(1	1.4286	0.0318	1.0999	0.3689	1.4095	0.0736	2.9758	0.0263	1.3343	0.0723	1.6345	0.2804	0.8620	0.2852	1.7751	0.5016	0.5854	0.4499
PC(13:0/22:6(4Z,7Z,10Z,13Z,16Z,1	1.9194	0.0318	1.1023	0.3185	1.6052	0.0246	1.4511	0.0263	1.4431	0.0723	0.8997	0.5017	1.8636	0.0505	1.7678	0.0638	1.0819	0.5604
PE(16:1(9Z)/22:4(7Z,10Z,13Z,16Z))	0.6490	0.0493	1.3025	0.0429	1.1203	0.0362	0.4898	0.0263	0.9625	0.1768	1.0692	0.4635	0.4167	0.0696	1.0122	0.5016	1.0817	0.4499
PC(15:0/20:5(5Z,8Z,11Z,14Z,17Z))_	2.0732	0.0318	1.3465	0.1231	1.9825	0.0246	1.3505	0.0263	0.9710	0.1768	0.7431	0.2804	1.4204	0.0505	1.5877	0.2731	0.7740	0.3099
PE(16:0/22:4(7Z,10Z,13Z,16Z))_PE	0.5178	0.0318	0.4078	0.0429	0.6285	0.0246	0.3345	0.0263	0.8019	0.1205	0.9065	0.5478	1.2100	0.1369	1.1820	0.3409	1.6123	0.1120
PC(18:3(6Z,9Z,12Z)/P-18:1(11Z))_F	1.2873	0.1793	1.1936	0.2029	1.2878	0.2742	2.8449	0.0263	0.8458	0.2663	1.3562	0.4635	0.3804	0.1100	5.6787	0.5426	0.1961	0.1576
PC(13:0/22:4(7Z,10Z,13Z,16Z))_PC	1.7097	0.0318	1.2801	0.0668	1.6335	0.0246	2.4262	0.0263	1.7212	0.0265	1.2580	0.2804	1.6413	0.0505	1.9676	0.1703	0.9604	0.5904
PE(16:1(9Z)/22:2(13Z,16Z))_PE(18	0.4989	0.0891	0.5547	0.2029	0.5270	0.0736	0.2678	0.1340	1.2763	0.2663	0.4352	0.4635	0.1981	0.0505	3.0291	0.3965	0.2134	0.1120
PC(18:2(9Z,12Z)/P-18:1(11Z))_PC(	2.4614	0.0493	1.6745	0.0668	3.2785	0.0246	2.6166	0.0375	1.2511	0.1502	1.2682	0.3336	0.7381	0.1674	1.1101	0.5016	0.5027	0.2076
PC(15:0/20:3(8Z,11Z,14Z))_PC(15:	1.5962	0.0318	1.2579	0.1231	1.6142	0.0246	1.5790	0.0263	1.2699	0.0723	1.0313	0.4038	1.4195	0.0696	1.5201	0.0638	0.8362	0.4499
PC(18:1(11Z)/P-18:1(11Z))_PC(18:	1.9865	0.0318	1.1012	0.4471	1.7393	0.0246	1.9062	0.0499	0.9735	0.2663	0.9416	0.5951	0.5017	0.1100	0.4006	0.1703	0.2769	0.2076
PC(13:0/22:2(13Z,16Z))_PC(15:0/2	2.2650	0.0318	1.9491	0.0429	2.7565	0.0246	1.4958	0.0375	1.1377	0.1768	0.6888	0.1939	2.0565	0.0505	1.5000	0.0939	1.0457	0.5604
PE(16:0/22:1(11Z))_PE(16:0/22:1(	1.0039	0.3269	0.2149	0.1565	0.8689	0.3095	0.0671	0.0375	1.3080	0.2041	0.5758	0.2547	0.7721	0.3316	0.5535	0.1703	0.3367	0.1120
PC(18:1(11Z)/P-18:0)_PC(20:1(11Z	1.4758	0.0493	1.1381	0.3185	1.4480	0.0246	2.2145	0.0263	1.2351	0.1502	1.2050	0.4635	0.5777	0.1369	0.8358	0.4575	0.3234	0.2076
PC(18:4(6Z,9Z,12Z,15Z)/18:4(6Z,9Z	1.2068	0.2896	1.5999	0.0668	1.0312	0.3095	2.9768	0.1630	14.9491	0.0265	1.1575	0.5951	Inf	0.2420	Inf	0.0638	Inf	0.5904
PE(17:1(9Z)/22:6(4Z,7Z,10Z,13Z,16	0.8762	0.2512	3.7811	0.0429	1.0349	0.3095	0.9376	0.1436	1.9809	0.0265	1.6135	0.1053	1.0129	0.3688	1.2466	0.3409	1.5649	0.1576
PE(P-18:0/22:6(4Z,7Z,10Z,13Z,16Z	1.9847	0.1123	59.4269	0.0429	0.7142	0.1645	2.6167	0.0878	1.2937	0.1502	1.1693	0.4038	34.9576	0.0505	55.0138	0.0638	1.5438	0.1576
PC(13:0/22:1(11Z))_PC(14:1(9Z)/2	1.9112	0.0318	1.6876	0.0429	1.5005	0.0246	2.4818	0.0263	2.1740	0.0265	1.1809	0.4038	1.4270	0.1369	1.7465	0.3409	0.7410	0.4499
PE(16:0/22:0)_PE(17:0/21:0)_PE(1	2.0265	0.1123	79.1743	0.0429	2.1272	0.0362	1.9591	0.0685	8.0048	0.0265	4.8548	0.1500	24.9091	0.0505	79.8203	0.0638	1.2631	0.3099
PC(O-16:0/20:1(11Z))_PC(O-16:0/2	1.1448	0.2512	2.2433	0.0429	1.1842	0.1286	2.6228	0.0263	1.2561	0.1768	1.2766	0.4635	0.6134	0.1369	3.1856	0.4575	0.4569	0.2076
PC(14:1(9Z)/22:6(4Z,7Z,10Z,13Z,16	1.4836	0.0318	1.0374	0.4152	1.4388	0.0362	1.0136	0.2349	1.0629	0.2340	0.5914	0.2804	2.2338	0.0505	1.9196	0.0638	0.8964	0.5604
PE(17:0/22:6(4Z,7Z,10Z,13Z,16Z,1	0.7890	0.0891	0.1896	0.0429	0.6337	0.0362	0.5002	0.0375	0.1153	0.0265	0.8509	0.3336	0.4617	0.0505	0.0536	0.0638	0.6532	0.2076
PE(O-18:0/22:6(4Z,7Z,10Z,13Z,16Z	0.5171	0.0649	1.3629	0.1231	1.5468	0.0362	4.1481	0.0263	3.6343	0.0265	3.2609	0.2547	3.4727	0.0505	5.4536	0.0638	3.1016	0.1120
PC(10:0/25:0)_PC(11:0/24:0)_PC(1	1.2937	0.1430	0.7412	0.0668	1.5203	0.0504	4.1882	0.0263	2.0644	0.0392	1.7924	0.3336	0.4291	0.2123	2.2423	0.5016	0.3715	0.3909
PC(14:0/22:6(4Z,7Z,10Z,13Z,16Z,1	1.2093	0.3269	0.7693	0.1565	1.1481	0.1286	1.6053	0.1120	5.8303	0.0392	1.6012	0.2804	1.4994	0.2501	9.9548	0.1703	0.5586	0.2593
PE(O-20:0/20:5(5Z,8Z,11Z,14Z,17Z	1.1469	0.1793	20.3269	0.0429	1.1288	0.0972	1.0562	0.2651	0.8320	0.1205	1.2109	0.2547	1.9445	0.0505	5.1890	0.3409	1.1942	0.4499
PC(14:0/22:5(4Z,7Z,10Z,13Z,16Z))_	1.3926	0.0649	1.0636	0.3185	1.3715	0.0504	2.3342	0.0263	2.4947	0.0265	1.4095	0.3336	1.6011	0.0505	3.2472	0.0638	1.0056	0.5604
PE(17:0/22:4(7Z,10Z,13Z,16Z))_PE	0.8491	0.2186	1.4301	0.4471	1.4384	0.0736	1.3446	0.1708	2.5082	0.0265	1.0158	0.5478	0.7631	0.2501	0.8379	0.5016	0.9170	0.5604
PE(O-18:0/22:4(7Z,10Z,13Z,16Z))_	2.7843	0.0649	0.1387	0.0842	1.0688	0.2008	0.6692	0.0878	0.1313	0.0265	0.7892	0.5017	1.2545	0.1674	0.5985	0.5016	0.7176	0.5604
PE(18:4(6Z,9Z,12Z,15Z)/22:6(4Z,7Z	0.2757	0.0318	14.8288	0.0429	6.1279	0.0736	0.4194	0.0878	0.0621	0.0392	0.1754	0.1500	0.6186	0.0696	0.3497	0.0939	0.1716	0.1576
PC(14:0/22:4(7Z,10Z,13Z,16Z))_PC	2.1368	0.0318	1.6585	0.0668	2.5274	0.0246	1.9301	0.0263	1.4923	0.0520	0.9599	0.5951	1.5879	0.0505	1.5213	0.0939	0.8777	0.4499
PE(17:1(9Z)/22:2(13Z,16Z))_PE(17	1.1561	0.1793	0.1982	0.0842	0.7414	0.1645	3.3550	0.0375	1.8788	0.2340	0.7252	0.2804	0.6713	0.2501	0.8222	0.5016	0.2783	0.2076
PC(O-17:0/20:4(5Z,8Z,11Z,14Z))	1.1001	0.2512	1.2791	0.2601	2.1168	0.0246	2.6356	0.0499	1.0587	0.2340	1.5293	0.4038	0.3594	0.1369	0.2872	0.1703	0.2182	0.2076
PE(18:3(6Z,9Z,12Z)/22:6(4Z,7Z,10Z	1.6370	0.0891	0.5816	0.2029	2.4416	0.2008	0.8526	0.1708	0.3697	0.0265	0.4719	0.1500	1.1780	0.0858	0.6501	0.2121	0.6670	0.2593
PC(14:1(9Z)/22:2(13Z,16Z))_PC(16	2.3936	0.0318	1.7715	0.0429	2.2160	0.0246	1.8764	0.0375	1.1179	0.1502	0.7501	0.2547	1.5008	0.0696	1.2867	0.1703	0.7456	0.2076
PC(14:0/22:2(13Z,16Z))_PC(14:1(9	2.1286	0.0318	1.6646	0.0429	1.9116	0.0246	1.8774	0.0263	1.2878	0.0723	0.9073	0.5478	1.8544	0.0505	1.3635	0.1703	1.0490	0.5904
PE(17:0/22:1(11Z))_PE(17:1(9Z)/2	0.9064	0.2186	1.3279	0.3185	0.5695	0.0736	0.3775	0.0685	0.2498	0.0520	0.2161	0.1500	1.6123	0.1100	0.3438	0.0638	0.2955	0.1120
PE(18:1(9Z)/22:6(4Z,7Z,10Z,13Z,16	0.8027	0.2896	27.1606	0.0429	0.4780	0.2008	1.8606	0.0685	1.0180	0.2340	1.0354	0.5951	27.9730	0.0505	19.1020	0.0638	1.6708	0.3909
PE(17:0/22:0)_PE(18:0/21:0)_PE(1	1.3415	0.0891	0.9133	0.4471	0.9530	0.2742	0.8997	0.1436	0.7659	0.0265	0.8601	0.3336	1.3764	0.0696	0.7744	0.3409	1.1607	0.5014
PC(14:0/22:1(11Z))_PC(14:0/22:1(	1.6728	0.0318	1.5596	0.0429	1.7360	0.0246	1.7643	0.0263	1.5252	0.0392	1.0867	0.4038	1.6282	0.0696	3.0030	0.0638	0.9210	0.5014
PC(15:1(9Z)/22:6(4Z,7Z,10Z,13Z,16	2.0602	0.0318	1.3481	0.0429	1.8663	0.0246	1.3553	0.0499	1.3061	0.0723	0.7762	0.2804	1.5263	0.0505	1.9275	0.0638	0.8537	0.5014
PC(20:5(5Z,8Z,11Z,14Z,17Z)/P-18:	1.4716	0.0891	0.9696	0.4152	1.7735	0.0246	1.6843	0.0685	1.3334	0.0926	0.9725	0.5951	0.8660	0.2501	8.9015	0.3965	0.5325	0.2593
PC(11:0/25:0)_PC(12:0/24:0)_PC(1	0.9942	0.3269	0.6990	0.1231	1.5086	0.0246	3.0974	0.0375	1.2188	0.1205	1.7143	0.2547	1.1165	0.3316	20.3556	0.2121	0.6913	0.5014
PE(18:1(9Z)/22:4(7Z,10Z,13Z,16Z))	0.5673	0.0493	0.0042	0.0429	0.2507	0.0246	0.3244	0.0263	0.0005	0.0265	0.3386	0.1053	1.2304	0.2123	0.0022	0.0638	0.4065	0.1120
PC(15:0/22:6(4Z,7Z,10Z,13Z,16Z,1	1.9706	0.0318	1.3827	0.0842	1.5876	0.0246	1.6423	0.0375	1.4792	0.0520	0.9749	0.5478	1.3642	0.1674	1.3103	0.2731	0.8233	0.3909
PC(20:4(5Z,8Z,11Z,14Z)/P-18:1(11Z	2.3226	0.0318	1.4605	0.0842	2.4918	0.0246	2.2283	0.0375	1.6413	0.0926	1.1231	0.5478	0.7750	0.2123	3.9778	0.5016	0.4280	0.2076
PC(15:1(9Z)/22:4(7Z,10Z,13Z,16Z))	1.5357	0.0318	1.0798	0.4471	1.3141	0.0246	1.8601	0.0263	1.4168	0.0723	1.1505	0.2804	1.0106	0.3316	0.9284	0.5016	0.6043	0.2076
PC(20:3(5Z,8Z,11Z)/P-18:1(11Z))_F	1.6145	0.0318	0.9851	0.4471	1.6504	0.0246	2.3195	0.0499	1.0003	0.2340	1.1870	0.5017	0.4190	0.1100	0.5523	0.2731	0.2777	0.1872
PC(15:0/22:4(7Z,10Z,13Z,16Z))_PC	1.7923	0.0318	1.4192	0.0429	2.3350	0.0246	1.8424	0.0263	1.4225	0.0926	1.1490	0.4635	1.0315	0.3316	1.3275	0.5016	0.5843	0.1872
PE(18:1(9Z)/22:2(13Z,16Z))_PE(18	0.9748	0.3269	1.6963	0.0429	1.5071	0.2008	0.4498	0.0375	0.6006	0.2663	0.4961	0.2804	1.5749	0.1674	1.9138	0.1282	2.5329	0.1120
PC(20:2(11Z,14Z)/P-18:1(11Z))_PC	1.5641	0.1430	0.8669	0.3689	1.6597	0.0246	2.4728	0.0685	1.3634	0.1205	1.3299	0.5017	0.4060	0.1100	0.8558	0.5016	0.2122	0.2076
PC(15:1(9Z)/22:2(13Z,16Z))_PC(17	1.7934	0.0318	1.3028	0.0668	1.7036	0.0246	1.2578	0.0685	0.8130	0.1768	0.5074	0.1053	1.6897	0.0505	1.1403	0.2731	0.6971	0.3099
PE(18:0/22:2(13Z,16Z))_PE(18:1(9	1.2012	0.2896	27.4741	0.0429	1.7456	0.0246	1.1586	0.1708	1.1774	0.2041	0.6422	0.3336	5.7905	0.0696	16.5088	0.0638	0.9842	0.5904
PC(O-18:0/20:3(8Z,11Z,14Z))_PC(C	0.9685	0.3269	1.1843	0.2601	2.2338	0.0246	0.4139	0.0263	0.8768	0.2041	0.9141	0.4038	0.0573	0.0505	0.4615	0.2731	0.1464	0.1120
PC(18:4(6Z,9Z,12Z,15Z)/20:5(5Z,8Z	1.1639	0.2896	0.4618	0.0668	0.8753	0.1645	1.6445	0.1120	8.5979	0.0265	1.6292	0.3336	2.4813	0.1674	13.3310	0.1703	0.3379	0.2593
PC(15:0/22:2(13Z,16Z))_PC(15:1(9	1.4215	0.1123	1.1178	0.4471	1.4357	0.0362	1.6181	0.0263	1.1541	0.1502	0.8934	0.5478	2.0124	0.0505	1.5126	0.0939	0.9595	0.5904
PE(18:0/22:1(11Z))_PE(18:0/22:1(	0.9212	0.3269	0.8885	0.4152	0.6154	0.1286	1.3441	0.1708	1.8391	0.0392	0.8900	0.3336	1.5552	0.1369	2.9678	0.0638	1.3327	0.4499
PC(18:3(6Z,9Z,12Z)/20:5(5Z,8Z,11Z	1.2727	0.0318	0.9467	0.4471	1.3371	0.0246	4.7277	0.0263	7.2733	0.0265	1.9854	0.1939	4.3939	0.0505	6.0015	0.0638	2.4451	0.1120
PE(19:1(9Z)/22:6(4Z,7Z,10Z,13Z,16	2.7510	0.0891	0.0243	0.0668	0.0440	0.0362	0.1211	0.0263	0.0000	0.0265	0.0890	0.1053	0.6626	0.2123	0.0000	0.0638	0.0824	0.1120
PC(15:0/22:1(11Z))_PC(15:1(9Z)/2	1.3848	0.0493	1.4342	0.0668	1.5011	0.0504	2.1369	0.0263	1.9193	0.0265	1.0195	0.5951	1.6228	0.0696	2.6409	0.0638	0.7371	0.5014
PC(16:1(7Z)/22:6(4Z,7Z,10Z,13Z,16	1.2769	0.1793	1.0301	0.3185	1.4845	0.0362	1.6673	0.0263	2.0848	0.0265	1.1347	0.5478	1.4469	0.1100	2.0273	0.0939	1.0569	0.5604
PE(19:0/22:6(4Z,7Z,10Z,13Z,16Z,1	4.0366	0.0493	0.0000	0.0429	7.2453	0.0736	0.3551	0.0375	0.0067	0.0265	0.3889	0.1500	0.2112	0.0505	0.0000	0.0638	0.2598	0.2593
PE(O-20:0/22:6(4Z,7Z,10Z,13Z,16Z	0.4432	0.0318	1.0674	0.4471	0.5795	0.0736	0.5025	0.0685	1.0868	0.2340	0.9712	0.5478	1.7446	0.3688	2.6665	0.0939	1.3204	0.3909
PC(16:0/22:6(4E,7E,10E,13E,16E,1	1.6796	0.0318	1.1599	0.2029	2.1259	0.0246	1.6298	0.0263	2.4525	0.0723	1.0470	0.5951	1.4819	0.1100	2.3281	0.1703	0.6667	0.3909
PE(20:5(5Z,8Z,11Z,14Z,17Z)/22:6(4	1.7080	0.1793	40.5854	0.0429	0.4760	0.0736	0.2196	0.0499	0.1696	0.0392	0.0621	0.1500	1.0707	0.3688	18.9956	0.5426	0.0977	0.1120
PC(16:0/22:5(4Z,7Z,10Z,13Z,16Z))_	2.0168	0.0318	1.2686	0.1231	1.8583	0.0246	1.8227	0.0263	1.5354	0.0723	0.9259	0.4635	1.0156	0.3688	1.8851	0.5426	0.6192	0.1576
PE(19:0/22:4(7Z,10Z,13Z,16Z))_PE	2.4884	0.0891	0.0000	0.0429	0.2589	0.0246	0.4369	0.0878	0.0000	0.0265	0.3308	0.1053	0.8411	0.2852	0.0005	0.0638	0.3373	0.1120
PE(O-20:0/22:4(7Z,10Z,13Z,16Z))	1.3702	0.1793	0.1138	0.0429	0.0879	0.0246	1.7761	0.0878	2.0159	0.0723	0.7970	0.4038	2.1052	0.1100	2.1876	0.0638	1.5992	0.3909
PC(16:0/22:4(7Z,10Z,13Z,16Z))_PC	1.8948	0.0318	1.3065	0.0668	1.8788	0.0246	1.7312	0.0263	0.9584	0.2340	0.8282	0.3336	1.3056	0.0858	1.6090	0.5426	0.6348	0.1576
PE(19:1(9Z)/22:2(13Z,16Z))_PE(20	1.3043	0.1793	0.0501	0.0429	0.0334	0.0246	2.3170	0.0685	2.7561	0.0392	0.7664	0.4635	2.4842	0.0858	2.5532	0.0638	1.8630	0.3099
PE(20:3(8Z,11Z,14Z)/22:6(4Z,7Z,10	1.8399	0.2186	369.1633	0.0429	0.7229	0.2226	2.8273	0.0499	3.3119	0.0265	0.9254	0.5951	5.3102	0.0505	5.1621	0.0638	3.2469	0.1120
PC(16:1(9Z)/22:2(13Z,16Z))_PC(18	1.4295	0.0493	1.2067	0.2029	1.7046	0.0246	1.8684	0.0263	1.5888	0.0265	1.0320	0.5017	2.1012	0.0505	2.0813	0.0638	1.0982	0.5014
PC(16:0/22:2(13Z,16Z))_PC(16:1(9	1.3592	0.0649	1.1896	0.1565	1.6692	0.0246	1.9184	0.0263	1.4547	0.0265	1.1058	0.5017	1.3961	0.0858	1.8792	0.1703	0.8194	0.4499
PC(17:2(9Z,12Z)/22:6(4Z,7Z,10Z,13	2.6348	0.0318	1.4330	0.1231	2.3954	0.0246	1.6433	0.1120	1.2882	0.0723	0.9898	0.5951	0.7463	0.2501	0.7614	0.3965	0.4741	0.2076
PE(20:1(11Z)/22:6(4Z,7Z,10Z,13Z,1	0.3838	0.0891	0.1962	0.0429	0.3554	0.0362	0.6552	0.1436	0.7010	0.1502	0.6356	0.2804	0.8333	0.2123	0.6032	0.1703	0.8573	0.5904
PC(14:0/24:1(15Z))_PC(16:0/22:1(	1.3426	0.1123	1.3469	0.0668	1.7065	0.0246	1.9835	0.0263	1.5188	0.0265	1.1697	0.4038	0.5039	0.1100	1.7533	0.5016	0.3990	0.2076
PE(O-20:0/22:0)	1.4412	0.0318	0.0000	0.0429	0.0680	0.0246	0.2452	0.0263	0.0000	0.0265	0.1159	0.1053	0.6083	0.1369	0.0000	0.0638	0.1115	0.1120
PC(17:1(9Z)/22:6(4Z,7Z,10Z,13Z,16	1.5123	0.0891	0.9778	0.3689	1.1515	0.0736	2.7381	0.0263	3.1834	0.0265	1.3458	0.4038	1.7672	0.0696	2.3572	0.1703	0.8428	0.5904
PE(20:0/22:6(4Z,7Z,10Z,13Z,16Z,1	1.6764	0.1123	0.5938	0.0429	0.4011	0.0246	1.5378	0.0375	0.3247	0.0265	0.3127	0.1053	1.9860	0.0505	0.4662	0.0939	0.5035	0.1872
PC(P-18:0/22:6(4Z,7Z,10Z,13Z,16Z	2.0142	0.0318	1.4541	0.0668	2.5666	0.0246	1.8821	0.0685	1.0417	0.2340	1.0195	0.5951	0.4336	0.1100	1.0762	0.5016	0.2705	0.2076
PC(12:0/26:0)_PC(13:0/25:0)_PC(1	1.0750	0.2896	1.5497	0.0842	1.5889	0.1645	2.4590	0.0375	1.3947	0.0392	1.5862	0.5017	1.3882	0.1674	2.3658	0.1703	0.9677	0.5904
PC(17:0/22:6(4Z,7Z,10Z,13Z,16Z,1	2.1806	0.0318	1.5737	0.1231	2.6588	0.0246	1.6208	0.0375	2.4209	0.0723	0.9198	0.4635	1.9887	0.0505	2.2537	0.3409	0.8441	0.5904
PE(20:1(11Z)/22:4(7Z,10Z,13Z,16Z	1.0715	0.2512	0.9577	0.4471	0.7626	0.2008	0.7459	0.1436	0.9539	0.2340	0.7511	0.2804	1.1810	0.2501	1.8334	0.0638	1.3426	0.2593
PC(22:4(7Z,10Z,13Z,16Z)/P-18:1(1	1.4820	0.0493	1.0575	0.4152	1.9141	0.0246	2.2813	0.0375	1.6053	0.0723	1.4088	0.4038	0.6151	0.1369	2.5604	0.5016	0.3789	0.2076

PC(16:0/23:5(8E,11E,14E,17E,20E)	1.7046	0.0891	1.4328	0.0668	2.0458	0.0246	2.1740	0.0263	1.6165	0.0520	1.3871	0.3336	0.6358	0.2123	1.7241	0.4575	0.4322	0.2593
PC(22:4(7Z,10Z,13Z,16Z)/P-18:0)_	2.5979	0.0318	1.3512	0.1565	1.5684	0.0504	2.9213	0.0263	1.4704	0.1205	1.4915	0.4038	0.4368	0.1100	0.5765	0.3409	0.3018	0.2076
PC(17:0/22:4(7Z,10Z,13Z,16Z))_PC	1.3424	0.0891	0.9415	0.4471	1.5809	0.0246	1.2997	0.1436	1.1430	0.2041	0.8285	0.5951	1.9954	0.0505	2.1356	0.2121	0.9195	0.5014
PE(20:1(11Z)/22:2(13Z,16Z))_PE(2	0.2390	0.0318	1.0176	0.4152	0.5839	0.0246	0.6544	0.1436	1.4613	0.0520	1.3679	0.2547	0.4381	0.0858	0.6868	0.5016	0.7063	0.4499
PC(22:2(13Z,16Z)/P-18:1(11Z))_PC	1.5296	0.1430	1.5667	0.0842	2.3662	0.0246	3.4751	0.0685	1.9374	0.0723	1.8907	0.2804	0.5116	0.1100	0.8471	0.5426	0.3340	0.2076
PC(18:4(6Z,9Z,12Z,15Z)/22:6(4Z,7Z	1.0842	0.2896	0.9051	0.4152	0.9953	0.3095	2.5973	0.0263	5.0487	0.0392	1.1089	0.5478	1.9373	0.1100	6.0781	0.0638	0.8094	0.3909
PC(17:1(9Z)/22:2(13Z,16Z))_PC(17	0.9753	0.2512	0.7669	0.0429	0.8318	0.0246	0.8092	0.0375	0.9084	0.1502	1.0992	0.4635	1.2602	0.1100	0.7775	0.2121	0.9940	0.5904
PE(20:0/22:2(13Z,16Z))_PE(20:1(1	1.9763	0.1793	0.4781	0.0429	2.3061	0.0736	1.6167	0.0878	0.9174	0.2340	0.7528	0.2547	1.2148	0.2852	0.7243	0.0638	1.1259	0.5014
PC(22:1(13Z)/P-18:1(11Z))_PC(22:	0.9525	0.2512	0.5648	0.0842	1.5582	0.0736	0.4692	0.0685	0.7443	0.1205	0.7349	0.1500	0.3842	0.1369	0.8717	0.4575	0.7841	0.4499
PC(18:3(6Z,9Z,12Z)/22:6(4Z,7Z,10Z	1.1257	0.2896	0.8856	0.3689	1.6568	0.0362	1.7909	0.0499	4.2721	0.0265	1.4878	0.3336	2.2068	0.0505	3.8876	0.0638	1.1299	0.5904
PC(17:0/22:2(13Z,16Z))_PC(17:1(9	0.5908	0.0493	1.0869	0.4471	0.9591	0.3095	0.8763	0.1708	0.7958	0.0926	0.6812	0.2547	0.3179	0.0858	0.6999	0.3409	0.1748	0.1576
PC(O-18:0/22:2(13Z,16Z))_PC(O-1	3.1740	0.0318	1.7504	0.2601	3.5090	0.0504	1.6020	0.1120	1.4920	0.1205	1.3755	0.4635	2.8547	0.2123	2.7457	0.1703	1.0638	0.5904
PC(18:2(9Z,12Z)/22:6(4Z,7Z,10Z,1	1.7486	0.0318	1.6476	0.0429	3.0020	0.0246	1.7947	0.0263	2.4975	0.0723	1.2054	0.5017	1.2073	0.3688	2.1022	0.0939	0.6391	0.3099
PE(20:0/22:0)_PE(21:0/21:0)_PE(2	1.4828	0.0318	1.3701	0.0429	1.2927	0.0246	1.0854	0.1708	0.6261	0.0265	0.6234	0.1053	0.5641	0.0505	0.4291	0.0638	0.5042	0.1120
PC(18:1(11Z)/22:6(4Z,7Z,10Z,13Z,	1.6542	0.0493	1.1100	0.3689	1.6137	0.0246	1.9473	0.0263	1.4423	0.0723	0.7545	0.4038	1.2498	0.2123	1.9338	0.2731	0.5874	0.2076
PE(22:6(4Z,7Z,10Z,13Z,16Z,19Z)/2	0.4563	0.0891	6.8953	0.0429	0.3473	0.0736	0.1810	0.0878	0.2330	0.0392	0.0000	0.1053	7.0196	0.2003	103.1287	0.1692	0.0000	0.4428
PC(18:0/22:6(4Z,7Z,10Z,13Z,16Z,1	1.6444	0.0318	1.2402	0.2601	1.9854	0.0246	1.5911	0.0263	1.3374	0.0520	0.9509	0.4635	2.0091	0.0505	2.1517	0.0638	1.0971	0.5604
PC(18:0/22:5(4Z,7Z,10Z,13Z,16Z))_	1.7797	0.0318	1.4736	0.0668	2.4614	0.0246	2.6235	0.0263	2.2730	0.0265	1.2373	0.4038	1.1102	0.2123	1.5715	0.3965	0.7163	0.3099
PE(21:0/22:4(7Z,10Z,13Z,16Z))_PE	0.2708	0.0318	0.6382	0.0429	0.6569	0.0972	0.5156	0.1980	3.2851	0.0392	1.7582	0.2804	2.7901	0.0696	2.2587	0.0638	2.9570	0.2076
PE(22:6(4Z,7Z,10Z,13Z,16Z,19Z)/2	0.9583	0.1793	0.0306	0.0429	2.1053	0.0736	1.6045	0.0263	0.3288	0.0265	0.6202	0.2547	1.2146	0.2123	0.2778	0.0638	0.3480	0.1872
PC(18:0/22:4(7Z,10Z,13Z,16Z))_PC	1.8626	0.0318	1.2072	0.2029	2.1538	0.0246	2.5856	0.0263	1.3321	0.0723	1.4747	0.4635	0.7559	0.1674	0.7440	0.3409	0.4500	0.1872
PC(18:0/22:3(10Z,13Z,16Z))_PC(18	1.3929	0.1430	1.2608	0.1565	1.8906	0.0246	2.0603	0.0375	1.5702	0.0265	0.9078	0.5478	0.7930	0.2123	1.2723	0.5016	0.5219	0.2076
PE(21:0/22:2(13Z,16Z))_PE(22:2(1	1.0925	0.2512	1.0737	0.0668	0.7862	0.0246	1.2886	0.1436	0.7017	0.0265	1.1510	0.4038	1.2846	0.2501	0.8087	0.2731	1.1397	0.5604
PC(16:1(9Z)/24:1(15Z))_PC(18:0/2	1.2301	0.2186	1.2454	0.1565	2.0623	0.0246	2.0627	0.0263	2.0877	0.0265	1.4310	0.1939	0.5492	0.2501	0.8484	0.5426	0.4126	0.2076
PE(22:1(11Z)/22:6(4Z,7Z,10Z,13Z,1	0.7255	0.1123	1.0175	0.4471	0.8486	0.0972	0.7846	0.0499	1.1211	0.1502	1.3542	0.1053	0.9771	0.2852	1.1313	0.2731	1.1885	0.5604
PC(16:0/24:1(15Z))_PC(18:0/22:1(	1.2305	0.2186	1.8464	0.0429	2.3613	0.0246	1.8679	0.0263	1.5663	0.0520	1.2249	0.5017	0.1413	0.1100	0.4347	0.2731	0.1592	0.2076
PC(19:1(9Z)/22:6(4Z,7Z,10Z,13Z,1	1.6452	0.1430	1.3371	0.1231	2.2138	0.0246	2.1741	0.0878	1.8460	0.0723	1.1165	0.5478	0.4922	0.1674	2.5494	0.3965	0.3304	0.3099
PC(P-20:0/22:6(4Z,7Z,10Z,13Z,16Z	1.3273	0.0891	1.0630	0.4471	1.2527	0.0736	1.8406	0.0263	1.1363	0.2041	1.0448	0.5478	1.6505	0.0858	0.8783	0.5016	0.6917	0.3099
PC(14:0/26:0)_PC(16:0/24:0)_PC(1	0.7451	0.0649	1.7534	0.1231	1.9079	0.0246	0.6999	0.1436	0.7813	0.1768	0.6318	0.3336	1.2190	0.2852	1.9823	0.0939	1.6198	0.1576
PC(19:0/22:6(4Z,7Z,10Z,13Z,16Z,1	1.1100	0.2512	0.9280	0.4471	1.1674	0.0736	1.9931	0.0375	2.2896	0.0392	0.9883	0.5951	2.9706	0.0505	2.9933	0.0638	1.1865	0.5904
PE(22:1(11Z)/22:4(7Z,10Z,13Z,16Z	1.6955	0.0891	0.6665	0.0429	0.9361	0.3095	0.9097	0.1980	0.7684	0.0265	0.7044	0.2804	1.2833	0.2501	0.9143	0.3965	0.8930	0.5604
PC(19:1(9Z)/22:4(7Z,10Z,13Z,16Z))	0.8080	0.0891	0.6093	0.2029	1.1937	0.1645	0.8597	0.2349	0.9344	0.2340	0.9420	0.5017	0.4403	0.1369	0.4889	0.1703	0.4517	0.2076
PC(20:5(5Z,8Z,11Z,14Z,17Z)/22:6(	1.8449	0.0493	1.5548	0.0668	2.2052	0.0246	2.1654	0.0263	2.9122	0.0265	1.2075	0.5478	1.3661	0.3316	1.7545	0.0939	0.6216	0.2593
PC(19:0/22:4(7Z,10Z,13Z,16Z))_PC	0.5048	0.0318	0.8651	0.3689	1.5605	0.0362	1.2540	0.2335	1.4326	0.2340	2.3469	0.2804	0.9682	0.3688	2.1318	0.3409	0.7401	0.5904
PC(O-20:0/22:4(7Z,10Z,13Z,16Z))	0.6297	0.1430	5.3519	0.0429	0.8775	0.1645	1.4822	0.0685	1.3396	0.0926	1.5949	0.3336	2.5658	0.0505	3.0134	0.0638	1.7285	0.1872
PC(20:4(5Z,8Z,11Z,14Z)/22:6(4Z,7Z	1.3186	0.0649	1.4666	0.1231	3.1247	0.0246	2.0845	0.0263	1.7861	0.0723	1.2669	0.4635	0.7979	0.1674	2.3687	0.5426	0.4085	0.1872
PE(22:0/22:2(13Z,16Z))_PE(22:1(1	2.7202	0.0318	0.6302	0.1231	1.2657	0.1645	1.3345	0.1436	1.1946	0.2340	0.7945	0.4635	3.0186	0.0505	1.7691	0.1703	1.9324	0.1872
PC(20:3(8Z,11Z,14Z)/22:6(4Z,7Z,1	1.6019	0.0318	1.3836	0.1565	2.4752	0.0246	1.9669	0.0263	1.3868	0.1502	1.1474	0.4038	0.8566	0.2501	1.1989	0.5016	0.4798	0.1872
PE(20:0/24:1(15Z))_PE(22:0/22:1(	1.2078	0.2186	0.7960	0.4471	4.2012	0.0246	0.8249	0.1708	0.6057	0.0723	1.0389	0.5478	0.9037	0.2852	0.9797	0.5426	1.0240	0.5904
PC(20:2(11Z,14Z)/22:6(4Z,7Z,10Z,	1.9587	0.0318	1.2032	0.2601	2.2354	0.0246	2.4021	0.0878	0.9267	0.2663	1.2699	0.4038	0.2951	0.0858	0.5260	0.3409	0.2391	0.1120
PC(19:0/22:1(11Z))_PC(19:1(9Z)/2	0.5067	0.1793	0.9347	0.4471	0.8087	0.1645	0.7333	0.2349	0.7799	0.1205	0.6505	0.2547	1.8792	0.1100	1.9822	0.0638	2.0249	0.1120
PC(20:1(11Z)/22:6(4Z,7Z,10Z,13Z,	1.4458	0.1123	0.7732	0.1231	1.1286	0.1286	1.0753	0.2651	0.3303	0.0392	0.8525	0.5478	0.6251	0.3316	0.7045	0.5426	0.4374	0.3909
PC(20:0/22:6(4Z,7Z,10Z,13Z,16Z,1	1.3454	0.1430	1.0076	0.4152	1.6262	0.0246	2.3190	0.0375	1.2163	0.1768	1.1558	0.5478	0.5850	0.1674	1.0309	0.5016	0.3740	0.2076
PC(20:0/22:5(7Z,10Z,13Z,16Z,19Z)	1.6218	0.0891	1.0868	0.3185	2.6878	0.0246	2.3844	0.0499	1.5411	0.0926	1.3185	0.5017	0.4006	0.0858	1.5225	0.5016	0.2648	0.2076
PC(18:3(6Z,9Z,12Z)/24:1(15Z))_PC	1.3381	0.1793	1.3534	0.4471	1.1406	0.3095	2.7394	0.0263	1.6661	0.0520	1.2816	0.5951	0.2921	0.0696	0.8635	0.5016	0.3106	0.1576
PC(20:1(11Z)/22:2(13Z,16Z))_PC(2	1.2042	0.1793	1.1725	0.1565	1.7921	0.0362	2.0010	0.0263	1.6666	0.0392	1.3388	0.4038	0.4900	0.1100	0.9195	0.5426	0.4051	0.2076
PC(16:0/26:2(5Z,9Z))_PC(18:1(11Z	1.1328	0.1123	1.0319	0.4471	1.7828	0.0362	2.0652	0.0263	1.5920	0.0520	1.2579	0.4635	0.2764	0.1100	0.4423	0.2731	0.2897	0.2076
PC(18:0/24:1(15Z))_PC(18:1(11Z)/	1.1708	0.2186	1.4190	0.0842	2.9977	0.0246	2.0129	0.0263	1.6988	0.0723	1.4250	0.3336	0.3408	0.0858	0.3190	0.0939	0.4296	0.3909
PC(21:0/22:6(4Z,7Z,10Z,13Z,16Z,1	0.6180	0.1430	15.3058	0.0429	1.2273	0.0736	0.7564	0.2651	0.8543	0.2340	0.6351	0.4635	2.6523	0.0505	3.6952	0.0638	2.0834	0.1576
PC(22:6(4Z,7Z,10Z,13Z,16Z,19Z)/2	1.1980	0.0891	1.3125	0.2029	2.7265	0.0246	1.8771	0.0263	1.7216	0.0723	1.1104	0.4635	0.7175	0.2123	4.9951	0.5016	0.4506	0.2076
PC(O-22:2(13Z,16Z)/22:3(10Z,13Z,	0.6633	0.1793	32.0999	0.0429	1.1352	0.1645	0.8378	0.1980	1.0202	0.2340	0.8517	0.5017	2.5010	0.0505	3.4344	0.0638	2.0191	0.1576
PS(22:6(4Z,7Z,10Z,13Z,16Z,19Z)/2	0.6787	0.0318	0.8860	0.3185	1.0043	0.2378	2.8367	0.0685	2.8099	0.0520	1.7376	0.5478	0.2357	0.1100	1.4737	0.3409	0.1118	0.1120
PC(O-22:1(13Z)/22:3(10Z,13Z,16Z)	1.3706	0.1793	3.7789	0.0429	4.8201	0.0246	0.5514	0.0375	1.1409	0.2041	0.9367	0.4038	0.7396	0.1674	1.5652	0.0638	1.5869	0.1120
PC(22:4(7Z,10Z,13Z,16Z)/22:6(4Z,	1.7134	0.0649	0.4906	0.0429	2.9059	0.0246	2.7412	0.0263	0.7725	0.2041	1.6451	0.2804	4.2803	0.0505	1.2131	0.3409	1.3574	0.1872
PS(22:4(7Z,10Z,13Z,16Z)/22:6(4Z,	1.0804	0.3269	0.1348	0.0429	1.0910	0.3095	1.6447	0.1120	0.7609	0.1768	1.2756	0.5017	0.4460	0.1100	5.9911	0.5426	0.2520	0.2076
PC(22:2(13Z,16Z)/22:6(4Z,7Z,10Z,	0.2613	0.0493	1.5674	0.2601	3.3571	0.0362	3.1750	0.1120	0.8510	0.2340	2.2700	0.4038	0.1166	0.0505	0.6763	0.4575	0.1275	0.1120
PC(22:1(11Z)/22:6(4Z,7Z,10Z,13Z,	1.1926	0.2512	1.1378	0.4471	1.5513	0.0362	2.2165	0.1708	0.5433	0.0723	1.4898	0.5478	0.1967	0.0696	1.4316	0.5426	0.2232	0.1576
PC(22:0/22:6(4Z,7Z,10Z,13Z,16Z,1	1.2910	0.1430	4.5016	0.0429	2.2400	0.0246	3.6905	0.0878	1.9989	0.0392	1.6735	0.4635	0.3636	0.1100	1.8174	0.5016	0.2742	0.2076
PC(22:1(11Z)/22:4(7Z,10Z,13Z,16Z	1.1650	0.1793	2.9994	0.0429	1.9372	0.0362	1.9486	0.0685	1.2361	0.1205	1.3114	0.4635	0.6324	0.0858	1.4552	0.5016	0.6347	0.2593
PC(22:0/22:4(7Z,10Z,13Z,16Z))_PC	0.7474	0.1430	1.3844	0.4471	1.3918	0.0246	0.9513	0.2651	0.5403	0.0265	0.3123	0.1053	0.9417	0.1674	1.0756	0.5016	0.7402	0.2593
PC(22:1(11Z)/22:2(13Z,16Z))_PC(2	0.6330	0.0891	1.7660	0.1231	0.6504	0.0246	0.7349	0.1120	0.5686	0.0392	0.4875	0.1053	0.9942	0.3688	1.4427	0.2121	0.9349	0.5904
PC(22:0/22:2(13Z,16Z))_PC(22:1(1	0.8027	0.2896	0.0181	0.0429	0.5951	0.2378	0.5816	0.0685	0.3560	0.0265	0.4162	0.1053	0.7521	0.2852	1.0699	0.4575	0.8456	0.2076
PC(20:0/24:1(15Z))_PC(22:0/22:1(	0.8257	0.2512	0.0036	0.0429	0.6785	0.0736	0.6990	0.1120	0.6592	0.0265	0.5651	0.1053	0.6948	0.1674	1.2271	0.3409	0.8892	0.4499
PC(23:1(9Z)/23:1(9Z))	0.7119	0.1430	0.3804	0.0429	0.5320	0.0246	0.5220	0.0375	0.5658	0.0392	0.5477	0.1500	0.6511	0.1674	1.3141	0.1703	1.0839	0.3099
PC(22:0/24:1(15Z))	0.8104	0.1430	2.5039	0.0668	0.8218	0.1286	0.5722	0.0375	0.7475	0.1205	0.7663	0.2547	0.6277	0.1674	1.6651	0.1282	1.5380	0.1120
PC(24:1(15Z)/24:1(15Z))	0.9692	0.3269	0.7848	0.2601	0.9766	0.2742	0.4464	0.0375	0.6067	0.0392	0.7830	0.2804	0.6431	0.1369	1.2799	0.3409	1.2182	0.2076

SFA saturated fatty acid
UFA unsaturated fatty acid
